# Inhibition of *Escherichia coli* glycosyltransferase MurG and *Mycobacterium tuberculosis* Gal transferase by uridine-linked transition state mimics

**DOI:** 10.1016/j.bmc.2010.02.026

**Published:** 2010-04-01

**Authors:** Amy E. Trunkfield, Sudagar S. Gurcha, Gurdyal S. Besra, Timothy D.H. Bugg

**Affiliations:** a Department of Chemistry, University of Warwick, Coventry CV4 7AL, United Kingdom; b Department of Biosciences, University of Birmingham, Edgbaston, Birmingham B15 2TT, United Kingdom

**Keywords:** Glycosyltransferase, Inhibition, Peptidoglycan biosynthesis, MurG, *Mycobacterium tuberculosis*

## Abstract

Glycosyltransferase MurG catalyses the transfer of *N*-acetyl-d-glucosamine to lipid intermediate I on the bacterial peptidoglycan biosynthesis pathway, and is a target for development of new antibacterial agents. A transition state mimic was designed for MurG, containing a functionalised proline, linked through the α-carboxylic acid, via a spacer, to a uridine nucleoside. A set of 15 functionalised prolines were synthesised, using a convergent dipolar cycloaddition reaction, which were coupled via either a glycine, proline, sarcosine, or diester linkage to the 5′-position of uridine. The library of 18 final compounds were tested as inhibitors of *Escherichia coli* glycosyltransferase MurG. Ten compounds showed inhibition of MurG at 1 mM concentration, the most active with IC_50_ 400 μM. The library was also tested against *Mycobacterium tuberculosis* galactosyltransferase GlfT2, and one compound showed effective inhibition at 1 mM concentration.

## Graphical abstract

**Figure:**
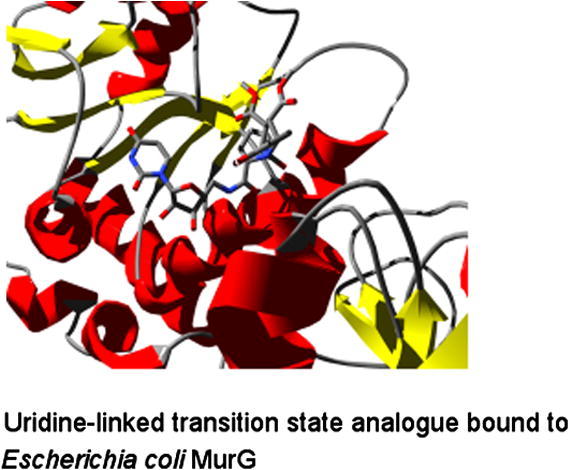

## 1 Introduction

**Figure 1:**
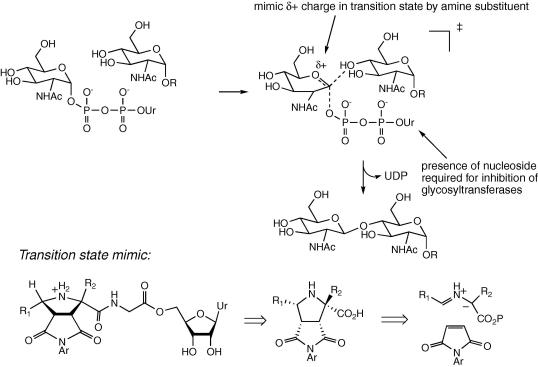
Glycosyl transfer reaction, showing presumed transition state, transition state mimic, and retrosynthetic disconnection.

Glycosyltransferase enzymes are involved in a wide range of biosynthetic pathways responsible for the formation of polysaccharides, glycoproteins, glycolipids and glycosylated natural products.^1^ The glycosyl transfer reaction involves transfer of a monosaccharide from a nucleotide mono/diphosphate donor to an acceptor alcohol, usually with inversion of stereochemistry at the anomeric centre, and the transition state for the reaction is thought to possess oxonium ion character,^1^ as shown in Figure 1.

Although a number of glycosyltransferases are potentially interesting targets for chemotherapeutic intervention, there are relatively few documented inhibitors for glycosyltransferases,^2–5^ compared with many examples of glycosidase enzyme inhibitors.^6^ Known inhibitors of glycosyltransferases in general contain the nucleoside found in the donor substrate: synthetic inhibitors include analogues of UDP-galactose in which the diphosphate linkage has been replaced by methylenediphosphate,^2^ or the ring oxygen replaced by a methylene group,^3^ or the glycosidic linkage replaced by a hydroxymethylene linkage.^4^ C-Glycosides have also been prepared, containing a linker to a uridine nucleoside, as inhibitors of chitin synthase.^5^

**Figure 2:**
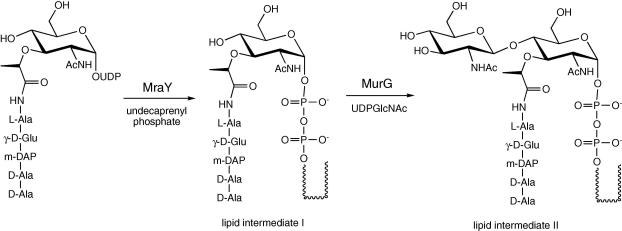
Reactions of the lipid-linked cycle of bacterial peptidoglycan biosynthesis catalysed by MraY and MurG.

Enzymes of the bacterial peptidoglycan biosynthetic pathway are well-established targets for antibacterial action. A lipid-linked cycle of reactions is responsible for transformation of cytoplasmic precursor UDPMurNAc-l-Ala-γ-d-Glu-*m*-DAP-d-Ala-d-Ala into peptidoglycan, via a series of lipid-linked reactions,^7^ shown in Figure 2. The first step, catalysed by translocase MraY, is the reaction of UDPMurNAc-pentapeptide with undecaprenyl phosphate to form lipid intermediate I: this reaction is inhibited by several nucleoside-containing natural products, upon which some structure–activity studies have been undertaken.^8^ The second step, catalysed by glycosyltransferase MurG, is the reaction of lipid intermediate I with UDPGlcNAc to form lipid intermediate II. *Escherichia coli* MurG is an extrinsic membrane protein,^9^ for which a crystal structure has been solved, in complex with UDPMurNAc.^10^ The *E. coli* enzyme has been overexpressed,^11^ and is able to accept synthetic lipid I analogues containing shortened prenyl chains.^12,13^ Using a fluorescent binding assay, several small molecule inhibitors of MurG have been identified by screening of combinatorial libraries.^14^ The active compounds are structurally unrelated to the enzyme substrates, and it is not known exactly how they bind to the enzyme.

**Figure 3:**
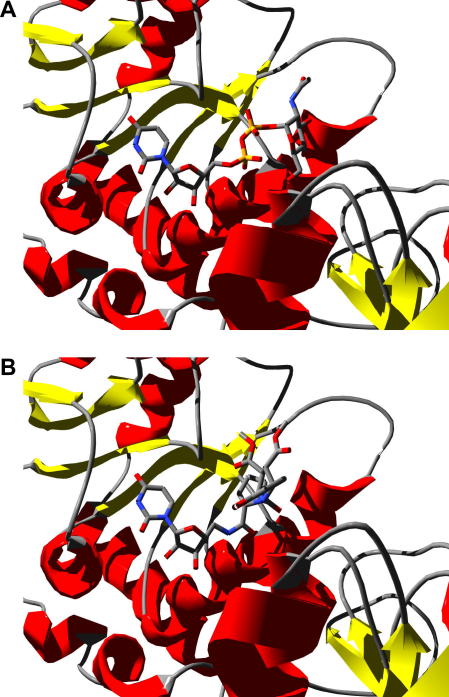
(A) Binding of UDPGlcNAc to *E. coli* MurG, showing twisted substrate conformation. (B) Example of docked inhibitor structure, showing positioning of proline substituent

Examination of the MurG structure reveals that there is are a series of specific interactions with the uracil base, and hydrogen-bonding interactions with the GlcNAc C-3 and C-4 hydroxyl groups, but no electrostatic interactions with the diphosphate bridge (see Fig. 3A).^10^ In order to prepare a substrate-based inhibitor for MurG, we have therefore designed a cyclic mimic for the oxonium ion transition state, linked via an uncharged spacer to a uridine nucleoside, shown in Figure 1. Adjacent to the GlcNAc binding site is a large cavity, lined with hydrophobic residues, therefore some members of the inhibitor set included an aromatic substituent able to bind to this site.

In this paper, we report the synthesis and screening of a set of transition state analogues using this design. The functionalised proline transition state mimic is readily assembled using a 1,3-dipolar cycloaddition sequence developed by Grigg and co-workers,^15^ which allows the convergent assembly of analogues containing a range of R_1_ groups (see Fig. 1). We have included a range of hydrogen-bond acceptor groups in the analogue set (methoxy-aryl substituents, ethane-1,2-diol substituent), in order to interact with the MurG active site. This approach could be used to inhibit other UDP-sugar glycosyltransferases, therefore we also report the screening of the inhibitor set against *Mycobacterium tuberculosis* galactosyl transferase.^16,17^

## 2 Results

### 2.1 Docking of transition state analogues into *E. coli* MurG active site

Several of the transition state analogue structures were energy minimised, and docked into the *E. coli* MurG active site (PDB file 1NLM) using eHiTS software.^18^ Analogues containing a glycine linker were found to be a suitable length to fit into the MurG active site, as shown in Figure 3B. Binding of the substrate UDPGlcNAc requires a twisted conformation at the GlcNAc-phosphate glycosidic linkage, in order to access the GlcNAc binding site, as shown in Figure 3A, and in several cases the docked proline substituent was found not to lie in the GlcNAc binding site. Therefore, conformationally flexible linkers were also included in the inhibitor collection, containing *N*-methyl glycine, proline amide linkers, or ester linkages, as described below.

### 2.2 Preparation of substituted prolines via 1,3-dipolar cycloaddition

Grigg and co-workers have previously demonstrated that imines formed between amino acid methyl esters and a range of aryl and alkyl aldehydes can undergo a 1,3-dipolar cycloaddition with *N*-phenyl maleimide,^15^ or with dimethyl maleate or dimethyl fumarate,^19^ to give a single major diastereoisomer product in each case. Using the imine formed between benzaldehyde and l-alanine methyl ester, cycloaddition reactions with *N*-phenyl maleimide were found to proceed with variable yield and product purity using the thermal cyclisation method,^19^ or using a one-pot acid-catalysed procedure also reported.^19^

**Scheme 1:**
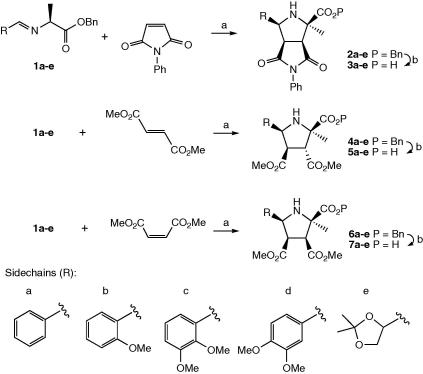
Preparation of substituted prolines via 1,3-dipolar cycloaddition. Reagents and conditions: (a) AgOAc, KOH, toluene, rt, 24 h; (b) H_2_, Pd/C.

A procedure involving 0.1 equiv of silver(I) acetate, carried out using the benzaldehyde imine of l-alanine isopropyl ester,^20^ was found to proceed in 40–88% yield and with high product purity after chromatography, however, subsequent hydrolysis of the isopropyl ester was found in our hands to be problematic. This latter procedure was found to work well using the benzaldehyde imine of l-alanine benzyl ester, to give cycloadduct **2a** with *N*-phenyl maleimide in 59% yield after flash chromatography. Benzyl ester **2a** was debenzylated in quantitative yield by hydrogenation using 10% palladium on carbon to give free acid **3a**, as shown in Scheme 1. Dipolar cycloadditions under the same reaction conditions with dimethyl fumarate and dimethyl maleate gave the benzyl esters **4a** and **6a** in 69% and 73%, respectively, which were debenzylated in 94% and 96% yield, respectively, to give free acids **5a**, and **7a**, as shown in Scheme 1.

In order to prepare enzyme inhibitors containing hydrogen bond acceptors in the sidechain R, imines of l-alanine benzyl ester were prepared from d-glyceraldehyde acetonide, *o*-anisaldehyde, 2,3-dimethoxybenzaldehyde, and 3,4-dimethoxybenzaldehyde. Dipolar cycloadditions with l-alanine benzyl ester, using the above conditions, proceeded well in each case to give a single major diastereoisomer, with yields of cycloadducts **2a**–**e**, **4a**–**e**, and **6a**–**e** in most cases in the range 50–75%, the highest yield being 96% for cycloadduct **2b**. Debenzylation proceeded smoothly, in most cases in 90–100% yield, to give the free acids **3a**–**e**, **5a**–**e**, and **7a**–**e**, as shown in Scheme 1.

In the case of diethyl fumarate cycloadducts **4a**–**e**, the major diastereomer obtained was the *endo*-isomer, as found by Casas et al.,^20^ with smaller amounts of the *exo*-diastereoisomer (epimer at C-3′′/C-4′′). Casas et al. reported a 94:6 ratio of *endo*:*exo* products, using the benzaldehyde imine of alanine *iso*-propyl ester;^20^ using the corresponding benzyl ester, we observed a 97:3 ratio of *endo*:*exo* products. For compounds with aryl R groups, only small amounts of the *exo*-diastereoisomer were formed (**4a** 3%; **4b** not detected; **4c** 16%; **4d** 13%); in the case of **4e**, the *exo*-isomer was formed as 40% of the isolated product. With the exception of **4e**, the minor *exo*-diastereoisomer was not observed in subsequent coupled products, following purification.

### 2.3 Coupling of glycine linker

**Scheme 2:**
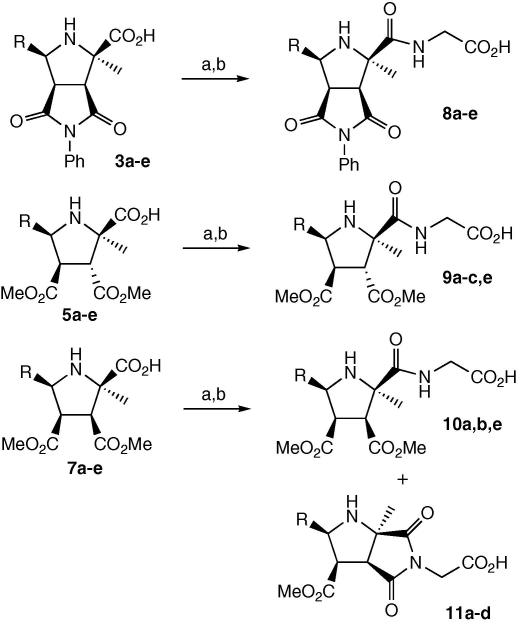
Coupling of glycine linker. Reagents and conditions: (a) H_2_NCH_2_CO_2_Bn, HATU, DIPEA, THF, 0 °C; (b) H_2_, Pd/C. R = Ph (a), 2-MeOPh (b), 2,3-(MeO)2Ph (c), 3,4-(MeO)_2_Ph (d), (isopropylidene)ethane-1,2-diol (e).

Since docking studies had indicated that a glycine linker was a suitable length for binding to MurG, each of the acids **3a**–**e**, **5a**–**e**, and **7a**–**e** was coupled with glycine benzyl ester, using either carbodi-imide EDCI in the presence of hydroxybenzotriazole (HOBt), or uronium coupling agent HATU, in the presence of HOBt, which in most cases proceeded in 60–90% yield. Debenzylation was achieved by hydrogenation using 10% palladium on carbon, in high yield, as illustrated in Scheme 2.

In the case of the dimethyl maleate cycloadducts **7a**–**e**, the coupled products were found to contain variable amounts of a bicyclic product, in which the nitrogen atom of the glycine linker had cyclised onto the *cis*-substituted methyl ester to form a cyclic imide. No cyclisation was observed in compound **10e** containing an aliphatic sidechain R, whereas compounds containing aromatic sidechains had cyclised to the extent of 50% for **10a/11a**, 67% for **10b/11b**, and 100% for **10c/11c** and **10d/11d**. The bicyclic compounds were not separable by silica chromatography from the uncyclised material, so compounds **10a/11a** and **10b/11b** were taken forward as mixtures.

### 2.4 Synthesis of uridine-containing analogues

**Scheme 3:**
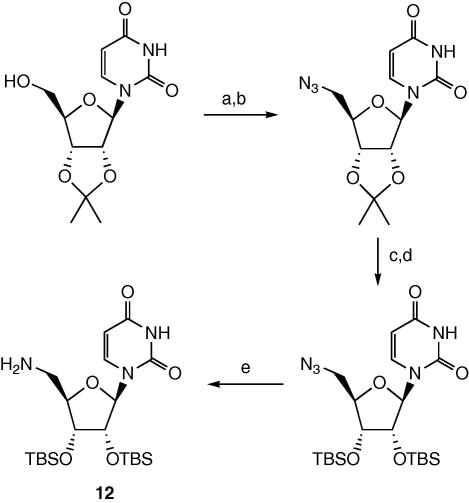
Preparation of 5′-amino,2′,3′-OTBDMS uridine. Reagents and yields: (a) TsCl, pyr, 49%; (b) NaN_3_, DMF, 50 °C, 100%; (c) CF_3_COOH, 95%; (d) TBSCl, imidazole, DMF, 81%; (e) H_2_, Pd/C, 100%.

Attempts to couple acid **8a** to the 5′-hydroxyl group of 2′,3′-isopropylidene-uridine, using a range of coupling methods, gave none of the desired ester-linked product after chromatography. In several reactions a new product was observed in the reaction mixture, using thin-layer chromatography, which was not observed after work-up and chromatography, suggesting that an ester linkage in this series of compounds may be unstable. Therefore, a 5′-amino, 5′-deoxyuridine derivative **12** containing TBDMS 2′-*O* and 3′-*O* protecting groups was prepared, as shown in Scheme 3. As observed previously,^21^ we found that in the presence of the 2′,3′-isopropylidene protecting group, a 5′-amino substituent was prone to intramolecular cyclisation onto the uracil base, but when re-protected as the 2′,3′-OTDBMS derivative, was quite stable.

**Scheme 4:**
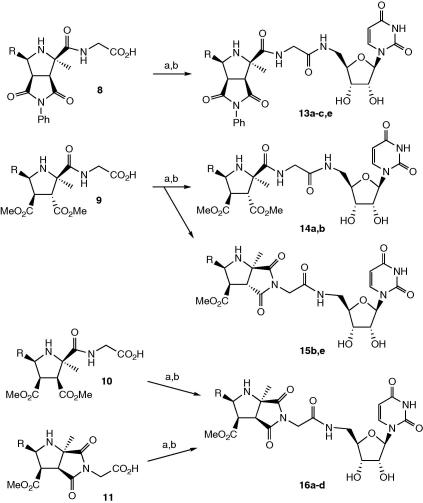
Coupling to 5′-amino, 5′-deoxyuridine. Reagents and conditions: (a) **9**, HATU, HOAt, DIPEA, THF, 0 °C; (b) CF_3_COOH, H_2_O/CH_2_Cl_2_. R = Ph (a), 2-MeOPh (b), 2,3-(MeO)_2_Ph (c), 3,4-(MeO)_2_Ph (d), ethane,1-2-diol (e).

Coupling of acid **8a** with **12** was found to proceed in 56% yield using HATU, in the presence of HOAt, to give the amide product, which was deprotected to give **10a**. The same procedure was used to couple the other analogues (see Scheme 4). After deprotection, the final products were purified by semi-preparative reverse phase HPLC. In the case of **13b**, two diastereoisomers were separated by HPLC.

The cyclisation of the nitrogen atom of the glycine linker to form a bicyclic imide was once again observed in the reaction products after coupling: for the dimethyl maleate adducts **10**, all of the reaction products **16a**–**d** contained only a bicyclic imide. Cyclisation was also observed to varying extents for the dimethyl fumarate adducts **9b** (partial cyclisation, products **14b** and **15b** separated by HPLC) and **9e** (product **15e** wholly cyclised), but not in the case of **9a** (product **14a**). The slower cyclisation of the dimethyl fumarate adducts can be explained by formation of the less favourable *trans*-fused bicyclic imide in these cases. Compounds **9e** and **15e** contained 40% of the C-3″/C-4″ epimer arising from the earlier cycloaddition reaction, whereas compounds **9a** and **9b** (and hence **14a**, **14b**, and **15b**) contained only a single diastereoisomer.

### 2.5 Synthesis of compounds containing conformationally flexible linkers

**Scheme 5:**
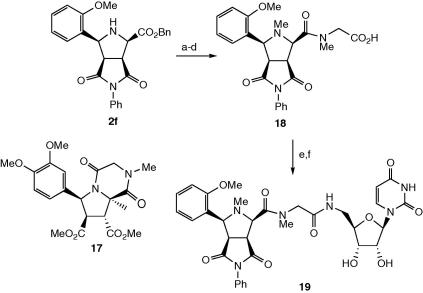
Synthesis of analogues containing N-substituted linkers. Reagents and conditions: (a) Me_2_SO_4_, NaH, THF/H_2_O, 35%; (b) H_2_, Pd/C, 100%; (c) H_2_NCH_2_CO_2_Bn, HATU, HOBt, DIPEA, THF, 0 °C, 49%; (d) H_2_, Pd/C, 100%; (e) **9**, HATU, HOAt, DIPEA, THF, 0 °C; (f) CF_3_COOH, H_2_O/CH_2_Cl_2_, 31% over two steps.

Since the earlier docking studies had indicated that the conformation of the bound UDPGlcNAc substrate contained a twisted conformation at the GlcNAc-phosphate glycosidic linkage, several analogues were synthesised containing conformationally flexible linkers. Coupling of acid **5c** with sarcosine (*N*-methyl glycine) benzyl ester, using the HATU/HOBt coupling used above, gave the unexpected reaction product **17**, in which the benzyl ester had undergone an intramolecular reaction with the endocyclic nitrogen atom. This reaction was unexpected, since earlier studies had found that the endocyclic nitrogen of derivatives **2a**–**e** was extremely unreactive towards acylation or alkylation reactions, however, such a cyclisation would be assisted by the presence of an *N*-methyl amide group. Similar intramolecular reactions were observed using acids **3c**, **7c**, and **5b**,

Therefore, methylation of the endocyclic nitrogen of derivative **2b** was undertaken, in order to prevent intramolecular cyclisation. Derivative **2b** was methylated in 38% yield using dimethyl sulphate using the method of Prashad et al.,^22^ however, after debenzylation, coupling of the free acid with sarcosine benzyl ester was unsuccessful. Therefore, compound **2f** lacking the α-methyl substituent was prepared by dipolar cycloaddition of the *o*-anisaldehyde imine of glycine benzyl ester with *N*-phenyl maleimide in 86% yield, followed by debenzylation. N*-*Methylation of **2f** using the method of Prashad et al.^22^ proceeded in 35% yield, and subsequent debenzylation proceeded in quantitative yield. Coupling with sarcosine benzyl ester, using HATU/HOBt, gave the desired amide product in 49% yield, which was debenzylated to give **18** (see Scheme 5). Coupling with 5′-amino, 5′-deoxy, 2′,3′-OTBDMS uridine **12** was carried out on a small scale, using the above method, and after deprotection and HPLC purification gave the sarcosine-linked analogue **19**.

**Scheme 6:**
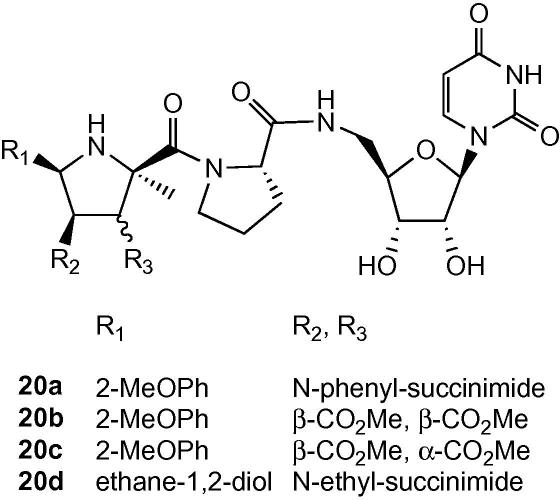
Analogues containing proline linker. Compound **20a**, R_1_ 2-MeOPh, R_2_/R_3_*N*-phenylsuccinimide; **20b**, R_1_ 2-MeOPh, R_2_ β-CO_2_Me, R_3_ β-CO_2_Me; **20c**, R_1_ 2-MeOPh, R_2_ β-CO_2_Me, R_3_ α-CO_2_Me; **20d**, R_1_ ethane-1,2-diol, R_2_/R_3_*N*-ethylsuccinimide.

Several compounds containing an l-proline linker were also prepared, which would possess a low energy barrier for *cis/trans*-amide bond interconversion. Acid **3b** was coupled to l-proline benzyl ester using HATU/HOBt in 91% yield, and debenzylated in 92% yield. Coupling with uridine derivative **12**, followed by deprotection, gave analogue **20a**. Using the same procedure, proline-containing analogues **20b**–**d** were prepared. Mixtures of *cis*- and *trans*-amide rotamers were observed for **20a**–**d** by NMR spectroscopy (Scheme 6).

**Scheme 7:**
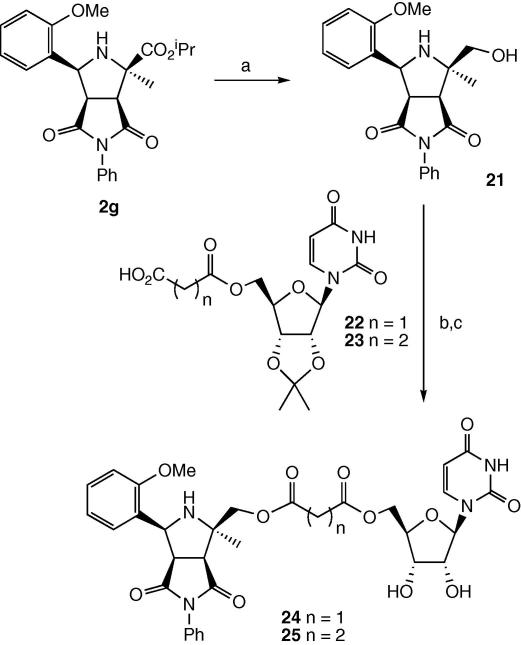
Synthesis of analogues containing ester linkages. Reagents and conditions: (a) DIBAL, THF, −78 °C, 26%; (b) EDCI/DMAP or HATU, THF, 0 °C; (c) CF_3_COOH/H_2_O, 16–24% over two steps.

Two compounds containing a diester linker were also prepared. Alcohol **21** was prepared by reduction of the isopropyl ester **2g** with DIBAL at −78 °C, in 26% yield. The 5′-*O-*succinyl uridine derivative **22** was prepared by treatment of 2′,3′-isopropylidene-uridine with succinic anhydride, while the 5′-*O-*malonyl derivative **23** was prepared by HATU/HOBt coupling of benzyl malonate with 2′,3′-isopropylidene-uridine, followed by debenzylation. Coupling of alcohol **21** with acid **22** was achieved using carbodi-imide EDCI, in the presence of DMAP, in 42% yield, and deprotected using aqueous trifluoroacetic acid, to give the succinyl diester **24**. Coupling of alcohol **21** with acid **23** using the same methods gave the malonyl diester **25**, as shown in Scheme 7.

### 2.6 Assay of compounds as inhibitors of *E. coli* MurG

Recombinant *E. coli* MurG-C-His_6_ was purified from *E. coli* strain C43 transformed with a pET3a vector containing the *murG* gene.^12^ From 2 l of cell culture, enzyme was purified by Ni^2+^ affinity HisTrap FPLC, yielding 19 mg of homogeneous protein. UDPMurNAc-pentapeptide was prepared from *Bacillus subtilis* W23, as previously described.^23^ MurG was assayed using a coupled radiochemical assay, as described by Zawadzke et al.,^24^ in which lipid intermediate I is generated in situ by membranes containing translocase MraY, and is then converted in the presence of [^3^H]-UDPGlcNAc and MurG to give radiolabelled lipid intermediate II, which is extracted into *n*-butanol and quantitated by scintillation counting. A time-course in the absence of inhibitor revealed that the product release was linear for 15–20 min, therefore, assays were recorded over 15 min. Treatment with 20 μM ramoplanin resulted in >90% inhibition, consistent with literature data.^25^

Eighteen compounds were tested as inhibitors of MurG, each at 1 mM final concentration. The results are shown in Table 1. Ten of the 18 compounds showed inhibition of MurG, the highest levels of inhibition by compounds **13b** (85% inhibition) and **14b** (32% inhibition). Selected compounds were then assayed at variable concentrations, and in each case, concentration-dependent inhibition was observed. Compounds **14b**, **15e**, and **20** each gave IC_50_ values of >5 mM, while compound **16d** showed IC_50_ 4 mM, and compound **13b** gave an IC_50_ value of 400 μM.

These compounds were tested for antibacterial activity against Gram-negative strains *E. coli* and *Pseudomonas putida*, and Gram-positive strains *Micrococcus luteus* and *B. subtilis*. Compound **13b** showed 50% growth inhibition of *M. luteus* at 100 μg/ml, but no effects upon the other strains.

### 2.7 Assay of compounds against *M. tuberculosis* galactosyltransferase

**Figure 4:**
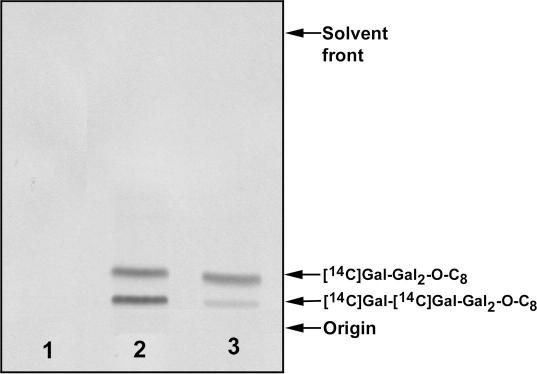
(A) TLC/autoradiogram of reactions products produced through the inclusion of acceptor Gal*f*(β1-6)Gal*f*-*O-*C_8_, mycobacterial membranes and UDP[^14^C]Galf. Lane 1, no acceptor; lane 2, 0.4 mM acceptor; and lane 3, 0.4 mM acceptor and 1 mM **16a**. Assay carried out as described in Section 4.

The arabinogalactan cell wall polymer of *M. tuberculosis* is biosynthesised through the action of a series of glycosyltransferases,^16^ several of which use UDP-sugar substrates, and therefore might be inhibited by this class of compound. We have characterised a UDP-galactose-dependent galactosyltransferase activity GlfT2, which can be assayed using specific neoglycolipid acceptors in the presence of membranes and Gal*f*(β1→6)Gal*f*-*O*-C_8_ resulting in excellent [^14^C]Gal*f* incorporation from UDP-[^14^C]Gal*p*, following endogenous conversion to UDP-[^14^C]Gal*f* and transferase activity (Fig. 4).^17^ TLC/autoradiography (Fig. 4) demonstrated the enzymatic conversion of the acceptor to both a trisaccharide product [^14^C]Gal to the 5′-OH of Gal*f*(β1→6)Gal*f*-*O*-C_8_ and a second, slower migrating band (Fig. 4) which based on relative migration profiles would be anticipated to be a tetrasaccharide product resulting from further elongation of the above trisaccharide precursor at the 6′OH consistent with the alternating linkage pattern of arabinogalactan. The complete chemical characterisation of the enzymatically synthesised products along with the identity of the galactosyltransferase gene product were previously reported.^17^

Assays were carried out in the presence of 14 of the compounds, at 0.5–1 mM concentration, as described in Table 1. Compound **16a** showed effective inhibition galactosyltransferase activity in the assay (Fig. 4).

## 3 Discussion

In this paper, we describe the synthesis and assay of a new family of glycosyltransferase transition state analogues, in which a uridine nucleoside is attached, via a variable length spacer, to a substituted proline analogue, which acts as an oxonium ion mimic. Using the 1,3-dipolar cycloaddition synthetic methodology developed by Grigg and co-workers,^19,20^ followed by a range of coupling methods, we have prepared a library of 19 analogues. One unexpected side-reaction was the intramolecular cyclisation of **10** to **11**, resulting in a number of bicyclic analogues.

Assay of the compound library as inhibitors of recombinant *E. coli* glycosyltransferase MurG revealed that 10 compounds showed some level of inhibition at 1 mM concentration, the highest activity being shown by compound **13b** (IC_50_ 400 μM). Most compounds showing inhibition of MurG contained a 2-methoxyphenyl R_1_ substituent, whilst no compound containing a phenyl R_1_ substituent inhibited MurG, consistent with a favourable hydrogen-bonding interaction between the 2-methoxy substituent and the MurG active site. Although the compounds were able to bind to MurG, the potency of inhibition was not as high as expected for binding of a transition state mimic, therefore it is suspected that the compounds do not adopt the desired conformation in the active site.

The crystal structure of *E. coli* MurG in complex with UDPGlcNAc shows that there is a significant twist in the conformation of the bound substrate between the diphosphate bridge and the anomeric centre. In order to encourage potential inhibitors to bind in such a conformation, a number of compounds containing conformationally flexible linkers were synthesised. Although compound **31** containing a sarcosine linker did bind to MurG, it bound no more effectively than the glycine analogue **13b**, whilst compounds **20a**–**d** containing a proline linker did not bind at all, implying that the MurG active site is unable to accommodate a more bulky substituent at this position. Compounds **24** and **25** containing a diester linker showed weak activity, but no improvement upon **13b**.

This set of compounds represents a new set of ligands for MurG, and the first inhibitors related in structure to the natural substrates. This strategy could in principle be used to design inhibitors for other glycosyltransferase enzymes that use a UDP-sugar substrate. Screening of this set of compounds against *M. tuberculosis* galactosyltransferase GlfT2 revealed that compound **16a** (which did not inhibit MurG) acted as an inhibitor for this enzyme. This is the first inhibitor that has been identified for this enzyme, which is a potential target for the development of new anti-TB drugs. Therefore, this strategy could be used to design inhibitors for other glycosyltransferase enzymes of therapeutic importance.

## 4 Experimental

### 4.1 Materials

d-Glyceraldehyde acetonide was prepared from d-mannitol using literature procedures,^26,27^ via bis-acetonide protection with SnCl_2_ and 2,2-dimethoxypropane, followed by oxidative cleavage by NaIO_4_. Di-*tert*-butyldimethylsilyl-5′-amino-5′-deoxyuridine (**12**) was prepared (see Scheme 3) by sodium azide displacement of 5′-tosyl-2′,3′-isopropylidene uridine, as described by Wang et al.,^28^ followed by deprotection of the acetonide as described by Winans and Bertozzi,^29^ followed by TBDMS protection and hydrogenation, as described by Dempcy et al.^30^ UDPMurNAc-l-Ala-γ-d-Glu-m-DAP-d-Ala-d-Ala was either prepared by the procedure previously described,^23^ or was purchased from the BACWAN synthesis facility (University of Warwick).

### 4.2 General procedure for 1,3-dipolar cycloaddition reaction to form **2a**–**g**, **4a**–**e**, **6a**–**e**, hydrogenation to form **3a**–**g**, **5a**–**e**, **7a**–**e**

Imines **1a**–**e** were formed using the method of Casas et al.^20^ l-Alanine benzyl ester *p*-tosylate (0.50 g, 1.48 mmol), aldehyde (1.48 mmol), sodium carbonate (0.16 g, 1.48 mmol) were suspended in water (30 ml) and stirred vigorously at room temperature for 20 h. The mixture was extracted with ethyl acetate (3 × 30 ml), dried (Na_2_SO_4_) and then concentrated in vacuo to yield the crude imino acyl ester, which was used immediately. The 1,3-dipolar cycloaddition was carried out using the method of Casas et al.^20^: The imino ester **1a** (est 0.26 g, 0.97 mmol), *N*-phenyl maleimide (0.19 g, 1.07 mmol), and silver(I) acetate (16 mg, 0.09 mmol) were suspended in toluene (15 ml). Potassium hydroxide (5 mg, 0.09 mmol) was then added and the mixture was stirred vigorously at room temperature for 24 h. The solvent was removed in vacuo and the residue was dissolved in ethyl acetate and percolated through a plug of silica gel, eluting with ethyl acetate. The filtrate was concentrated and the resulting material was purified by flash chromatography (eluent 3:2, hexane/ethyl acetate) to yield a white solid, **2a** (0.25 g, 0.57 mmol, 59%).

*Data for **2a***: Melting point 183–184 °C. ^1^H NMR (CD_3_CN, 400 MHz) *δ* 7.49–7.09 (15H, m, Ar-H), 5.27 (1H, d, *J* = 12.0 Hz, C*H*HPh), 5.18 (1H, d, *J* = 12.0 Hz, CH*H*Ph), 5.02–4.98 (1H, m, H5′′), 3.86 (1H, dd, *J* = 9.5, 9.5 Hz, H4′′), 3.65 (1H, d, *J* = 9.5 Hz, H3′′), 2.84–2.83 (1H, m, NH1′′), 1.69 (3H, s, 2′′-C*H*_3_); ^13^C NMR (CDCl_3_, 100 MHz) *δ* (not all quaternary C’s seen) 138.7, 136.2, 128.8, 128.3, 128.1, 127.9, 127.9, 127.6, 127.3, 126.6, 66.9, 66.5, 61.1, 55.2, 49.4, 22.7; *m*/*z* (ESI, +ve ion) 479.0 (MK)^+^, 463.1 (MNa)^+^, 441.2 (MH)^+^; *ν*_max_ 3335, 1726, 1702 cm^−1^; HRMS calcd for C_27_H_24_N_2_O_4_ (M+H^+^) 441.1814; found 441.1818. *Data for **4a***: colourless oil (0.34 g, 0.82 mmol, 69%). ^1^H NMR (CDCl_3_, 400 MHz) *δ* 7.43–7.23 (10H, m, Ar-H), 5.33 (1H, d, *J* = 12.5 Hz, C*H*HPh), 5.25 (1H, d, *J* = 12.5 Hz, CH*H*Ph), 4.83 (1H, d, *J* = 9.5 Hz, H5′′), 4.02 (1H, d, *J* = 9.5 Hz, H3′′), 3.88 (1H, dd, *J* = 9.5, 9.5 Hz, H4′′), 3.61 (3H, s, CO_2_C*H*_3_), 3.17 (3H, s, CO_2_C*H*_3_), 1.42 (3H, s, 2′′-C*H*_3_); ^13^C NMR (CDCl_3_, 100 MHz) *δ* 172.4, 171.9, 171.4, 139.2, 135.5, 128.6, 128.4, 128.3, 127.9, 127.7, 127.3, 67.6, 67.2, 62.9, 53.7, 52.5, 52.1, 51.5, 21.3; *m*/*z* (ESI, +ve ion) 434.1 (MNa)^+^, 412.2 (MH)^+^; *ν*_max_ 2950, 1728 cm^−1^; HRMS calcd for C_23_H_25_NO_6_ (M+H)^+^ 412.1760; found 412.1777. *Data for **6a***: colourless oil (0.26 g, 0.63 mmol, 73%). ^1^H NMR (CDCl_3_, 400 MHz) *δ* 7.38–7.25 (10H, m, Ar-H), 5.26 (1H, d, *J* = 12.0 Hz, CHHPh), 5.15 (1H, d, *J* = 12.0 Hz, CHHPh), 4.63 (1H, d, *J* = 6.5 Hz, H5′′), 3.46–3.42 (4H, m, H4′′ and CO2CH3), 3.28 (1H, d, *J* = 7.0 Hz, H3′′), 3.25 (3H, s, CO2CH3), 1.71 (3H, s, 2′′-CH3); ^13^C NMR (CDCl3, 100 MHz) *δ* 174.1, 171.1, 170.7, 137.4, 135.4, 128.8, 128.5, 128.4, 128.3, 127.8, 126.8, 68.4, 67.6, 63.7, 57.7, 53.1, 51.8, 51.3, 28.6; *m*/*z* (ESI, +ve ion) 450.1 (MK)^+^, 434.1 (MNa)^+^, 412.2 (MH)^+^; *ν*_max_ 3032, 2949, 1729 cm^−1^; HRMS calcd for C_23_H_25_NO_6_ (M+H)^+^ 412.1760; found 412.1768. Data for **2b**–**g**, **4b**–**e**, **6b**–**e** in Supplementary data.

### 4.3 Method for hydrogenation

The benzyl ester (0.20 g, 0.45 mmol) was dissolved in THF (20 ml), and 10 mol % of 10% palladium on carbon was added. The solution was stirred under a hydrogen atmosphere at room temperature for 3 h. The solution was then filtered through Celite and concentrated in vacuo to yield the deprotected acid.

*Data for **3a***^16^: White solid (0.16 g, 0.45 mmol, 100%). ^1^H NMR (CD_3_OD, 400 MHz) *δ* 7.46–7.14 (10H, m, Ar-H), 4.97 (1H, d, *J* = 9.5 Hz, H5′′), 3.84 (1H, dd, *J* = 7.5, 9.5 Hz, H4′′), 3.56 (1H, d, *J* = 7.5 Hz, H3′′), 1.68 (3H, s, Me); ^13^C NMR (CD_3_OD, 100 MHz), 175.2, 172.1, 172.0, 138.1, 133.5, 129.9, 129.6, 129.5, 129.2, 128.5, 127.9, 66.9, 63.6, 57.2, 51.7, 23.8; *m*/*z* (ESI, +ve ion) 395.2 (MNa_2_)^+^, 389.0 (MK)^+^, 373.1 (MNa)^+^, 351.1 (MH)^+^, 305.2 (MH−CO_2_H)^+^; *ν*_max_ 2964, 1707 cm^−1^. *Data for **5a***: white solid (0.21 g, 0.65 mmol, 96%). Melting point 198–200 °C (dec). ^1^H NMR (CD_3_CN, 400 MHz) *δ* 7.32–7.16 (5H, m, Ar-H), 4.87 (1H, d, *J* = 9.0 Hz, H5′′), 3.72 (1H, d, *J* = 7.5 Hz, H3′′), 3.65 (1H, dd, *J* = 7.5, 9.0 Hz, H4′′), 3.61 (3H, s, CO_2_C*H*_3_), 3.08 (3H, s, CO_2_C*H*_3_), 1.30 (3H, s, 2′′-CH_3_); ^13^C NMR (CD_3_CN, 100 MHz) *δ* (quaternary C’s not seen) 127.9, 127.5, 127.1, 62.3, 52.8, 51.9, 51.6, 50.9, 20.1; *m*/*z* (ESI, +ve ion) 344.1 (MNa)^+^, 322.1 (MH)^+^. *Data for **7a***: white solid (0.18 g, 0.57 mmol, 94%). Melting point 200–202 °C (dec). ^1^H NMR (CD_3_OD, 400 MHz) *δ* 7.44–7.39 (5H, m, Ar-H), 5.10 (1H, d, *J* = 7.5 Hz, H5′′), 3.83 (1H, dd, *J* = 7.5, 7.5 Hz, H4′′), 3.74 (3H, s, CO_2_C*H*_3_), 3.55 (1H, d, *J* = 7.5 Hz, H3′′), 3.32 (3H, s, CO_2_C*H*_3_), 1.81 (3H, s, 2′′-C*H*_3_); ^13^C NMR (CD_3_OD, 100 MHz) *δ* (not all quaternary C’s seen) 171.6, 134.1, 130.2, 130.0, 128.0, 72.4, 62.7, 56.0, 52.9, 52.0, 51.9, 25.1; *m*/*z* (ESI, +ve ion) 344.1 (MNa)^+^, 322.1 (MH)^+^, 276.2 (MH−CO_2_H)^+^; *ν*_max_ 3006, 2951, 1743, 1724, 1655 cm^−1^; HRMS calcd for C_16_H_19_NO_6_ (M+H)^+^ 322.1291; found 322.1280. Data for **3b**–**g**, **5b**–**e**, **7b**–**e** given in Supplementary data.

### 4.4 General procedure for coupling of glycine linker to form **8a**–**c**,**e**, **9a**–**e**, **10a**,**b**,**e**, **11a**–**d**

#### 4.4.1 Method for EDCI coupling

The cycloadduct **3a** (0.10 g, 0.29 mmol) was dissolved in dry DCM (5 ml) and cooled to 0 °C. EDCI·HCl (60 mg, 0.32 mmol) was then added followed by HOBt (49 mg, 0.32 mmol). The mixture was stirred for 10 min at 0 °C and then a solution of glycine benzyl ester tosylate (0.11 g, 0.32 mmol) and triethylamine (44 μl, 0.32 mmol) in dry DCM was added. The mixture was stirred at 0 °C for 2 h and was then allowed to warm to room temperature and stirred for a further 24 h. The solvent was removed in vacuo and the residue was dissolved in ethyl acetate (10 ml) and water (10 ml). The organic phase was washed with brine (3 × 10 ml), dried (MgSO_4_) and concentrated in vacuo to yield the crude product. The product was purified by flash chromatography (1:1, hexane/ethyl acetate) to yield the coupled benzyl ester as a white solid (0.11 g, 0.23 mmol, 79%). The benzyl ester (0.10 g, 0.20 mmol) was dissolved in methanol (10 ml) and 10 mol % of 10% palladium on carbon was added. The solution was stirred under a hydrogen atmosphere at room temperature for 3 h. The solution was then filtered through Celite and concentrated in vacuo to yield **8a** as a white solid (80 mg, 0.2 mmol, 100%). Melting point 149–153 °C. ^1^H NMR (CD_3_OD, 400 MHz) *δ* 7.29 (2H, d, *J* = 7.5 Hz, Ar-H), 7.18–7.15 (2H, m, Ar-H), 7.09–7.07 (1H, m, Ar-H), 7.05–7.00 (2H, m, Ar-H), 6.91–6.87 (3H, m, Ar-H), 4.87 (1H, d, *J* = 6.0 Hz, H5′′), 4.14 (1H, d, *J* = 17.5 Hz, NHC*H*H of glycine linker), 4.08 (1H, d, *J* = 17.5 Hz, NHCH*H* of glycine linker), 3.64 (1H, dd, *J* = 6.0, 8.5 Hz, H4′′), 3.52 (1H, d, *J* = 8.5 Hz, H3′′), 1.59 (C*H*_3_); ^13^C NMR (CD_3_OD, 100 MHz) *δ* 179.1, 176.6, 170.7, 170.7, 138.5, 137.5, 129.5, 129.4, 129.1, 127.9, 125.7, 122.2, 68.8, 68.7, 56.8, 55.2, 40.7, 23.1; *m*/*z* (ESI, +ve ion) 430.1 (MNa)^+^, 408.1 (MH)^+^, 392.2 (MH–Me)^+^; *v*_max_ 3301, 1701, 1697 cm^−1^; HRMS (LSIMS, +ve ion) calcd for C_22_H_21_N_3_O_5_ (M+H)^+^ 408.1559; found 408.1564.

#### 4.4.2 Method for HATU coupling

The cycloadduct **3b** (0.11 g, 0.29 mmol) was dissolved in dry THF (10 ml) and cooled to 0 °C. HATU (0.14 g, 0.38 mmol) was then added followed by HOAt (52 mg, 0.38 mmol) or HOBt. The mixture was stirred for 10 min at 0 °C and then DIPEA (75 mg, 0.58 mmol) was added, followed by glycine benzyl ester tosylate (0.11 g, 0.32 mmol) and dry THF (10 ml). The mixture was stirred at 0 °C for 2 h and then allowed to warm to room temperature and stirred for a further 24 h. The solvent was removed in vacuo and the residue was dissolved in ethyl acetate (10 ml) and water (10 ml). The organic phase was washed with brine (3 × 10 ml), dried (MgSO_4_) and concentrated in vacuo to yield the crude product. The product was purified by flash chromatography (4:1, ethyl acetate/hexane) to yield the coupled benzyl ester as a white solid (0.15 g, 0.29 mmol, 99%). The benzyl ester (0.13 g, 0.25 mmol) was dissolved in methanol (10 ml) and 10 mol % of 10% palladium on carbon was added. The solution was stirred under a hydrogen atmosphere at room temperature for 3 h. The solution was then filtered through Celite and concentrated in vacuo, to give **8b** as a white amorphous solid (0.10 g, 0.23 mmol, 93%). ^1^H NMR (CD_3_OD, 400 MHz) *δ* 7.68–7.66 (0.6H, d, *J* = 7.5 Hz, Ar-H), 7.36–6.81 (8.4H, m, Ar-H), 5.15–5.13 (1H, m, H5′′), 4.20 (0.4H, d, *J* = 17.0 Hz, NHC*H*_2_ of glycine linker), 4.14 (0.4H, d, *J* = 17.0 Hz, NHC*H*_2_ of glycine linker), 4.03–3.95 (1H, m, NHC*H*_2_ of glycine linker, H4′′), 3.91–3.83 (4.2H, m, OC*H*_3_, NHC*H*_2_ of glycine linker, H4′′), 3.66 (0.4H, d, *J* = 9.0 Hz, H3′′), 3.43 (0.6H, d, *J* = 8.0 Hz, H3′′), 1.69 (1.2H, s, 2′′-C*H*_3_), 1.65 (1.8H, s, 2′′-C*H*_3_); ^13^C NMR (CD_3_OD, 100 MHz) *δ* 176.9, 176.0, 159.0, 158.0, 138.3, 133.5, 130.4, 129.9, 129.6, 129.6, 128.0, 127.6, 125.9, 122.5, 121.5, 121.4, 111.3, 68.4, 63.9, 57.9, 56.6, 56.3, 56.2, 56.1, 52.7, 48.5, 42.5, 23.0, 22.8; *m*/*z* (ESI, +ve ion) 482.1 (MNa_2_)^+^, 460.1 (MNa)^+^, 438.2 (MH)^+^, 395.2 (MH−CO_2_H)^+^; *v*_max_ 3374, 3303, 2944, 1701, 1685, 1654 cm^−1^; HRMS (LSIMS, +ve ion) calcd for C_23_H_23_N_3_O_6_ (M+H)^+^ 438.1665; found 438.1680.

*Data for **9a***: white solid, 56 mg (EDCI coupling 57%, deprotection 71%). Melting point 144–146 °C. ^1^H NMR (CD_3_OD, 400 MHz) *δ* 7.30 (2H, d, *J* = 8.5 Hz, Ar-H), 7.20–7.13 (3H, m, Ar-H), 4.87 (peak hidden behind D_2_O, H5′′), 3.88 (2H, s, NHC*H*_2_ of glycine linker), 3.73 (1H, dd, *J* = 8.5, 8.5 Hz, H4′′), 3.62–3.61 (4H, m, H3′′ and CO_2_C*H*_3_), 3.04 (3H, s, CO_2_C*H*_3_), 1.30 (3H, s, 2′′-CH3); ^13^C NMR (CD3OD, 100 MHz) *δ* 177.2, 173.2, 173.2, 173.0, 141.9, 129.1, 128.8, 128.7, 67.8, 63.4, 54.3, 53.8, 52.7, 52.0, 42.2, 21.3; *m*/*z* (ESI, +ve ion) 417.1 (MK)^+^, 401.1 (MNa)^+^, 379.1 (MH)^+^; *v*_max_ 3345, 3302, 1719, 1658 cm^−1^; HRMS calcd for C_18_H_22_N_2_O_7_ (M+H)^+^ 379.1505; found 379.1516.

*Data for **10a**/**11a***: white solid, 0.16 g (EDCI coupling 32%, deprotection 97%), containing **10a** and bicyclic **11a** in a 1:1 molar ratio. ^1^H NMR (CD_3_OD, 400 MHz) *δ* 7.48 (2H, d, *J* = 7.5 Hz, Ar-H) 7.22 (3H + 5H, m, Ar-H), 5.01 (1H, d, *J* = 6.5 Hz, H5′′ **11a**), 4.95 (1H, d, *J* = 7.0 Hz, H5′′ **10a**), 4.26 (1H, d, *J* = 17.5 Hz, NHC*H*H of glycine linker **11a**), 4.18 (1H, d, *J* = 17.5 Hz, NHCH*H* of glycine linker **11a**), 4.02 (1H, d, *J* = 18.0 Hz, NHC*H*H of glycine linker **10a**), 3.97 (1H, d, *J* = 18.0 Hz, NHCH*H* of glycine linker **10a**), 3.75 (1H, dd, *J* = 6.5, 8.0 Hz, H4′′ **11a**), 3.70 (3H, s, CO_2_C*H*_3_ **11a**), 3.56 (2H, m, H3′′ **11a** and H4′′ **10a**), 3.46 (1H, d, *J* = 7.0 Hz, H3′′ **10a**), 3.18 (3H, s, CO_2_C*H*_3_ **10a**), 3.13 (3H, s, CO_2_C*H*_3_ **11a**), 1.73 (3H, s, 2′′-C*H*_3_ **11a**), 1.65 (3H, s, 2′′-C*H*_3_ **11a**); ^13^C NMR (CD_3_OD, 100 MHz) *δ* 178.7, 176.4, 172.9, 172.6, 172.5, 170.5, 138.5, 137.4, 129.4 129.4, 128.5, 127.8, 68.0, 67.9, 63.3, 56.8, 55.7, 53.7, 53.2, 52.8, 52.2, 51.9, 50.0, 42.4, 40.7, 27.2, 22.42; *m*/*z* (ESI, +ve ion) 401.1 (M(**10a**)Na)^+^, 379.1 (M(**10a**)H)^+^, 347.1 (M(**11a**)H)^+^; *v*_max_ 3328, 2989, 1746, 1711 cm^−1^; HRMS (LSIMS, +ve ion) calcd for C_18_H_22_N_2_O_7_ (M**_a_**+H)^+^ 379.1497; found 379.1505. Data for **8c**, **8e**, **9b**–**e**, **10b/11b**, **10e**, **11c**–**d** given in Supplementary data.

### 4.5 General procedure for uridine coupling and deprotection

The cycloadduct-linker (**8b**, 70 mg, 0.16 mmol) was dissolved in dry THF (10 ml) and cooled to 0 °C. HATU (80 mg, 0.21 mmol) was then added followed by HOAt (28 mg, 0.21 mmol). The mixture was stirred for 10 min at 0 °C and then DIPEA (40 mg, 0.32 mmol) was added followed by 5′-amino, 5′-deoxy-2′, 3′-*O*-bis(*tert-*butyldimethylsilyl) uridine (**12**, 80 g, 0.18 mmol). The mixture was stirred at 0 °C for 2 h and then allowed to warm to room temperature and stirred for a further 48 h. The solvent was removed in vacuo and the residue was dissolved in ethyl acetate (10 ml) and water (10 ml). The organic phase was washed with brine (3 × 10 ml), dried (MgSO_4_) and concentrated in vacuo to yield the crude product. The product was purified by flash chromatography (1:1 hexane/ethyl acetate, 100% ethyl acetate) to yield the coupled product as a pure white solid, (50 mg, 0.06 mmol, 36%) (1:1 ratio of diastereomers **a** and **b**). Melting point 174–179 °C. ^1^H NMR (CDCl_3_, 400 MHz) *δ* 8.10 (1H, br t, *J* = 11.4 Hz, N*H*CH_2_ **a** of glycine linker), 7.99–7.95 (1H, m, N*H*CH_2_ **b** of glycine linker), 7.46 (1H, br d, *J* = 8.5 Hz, H6 **b**), 7.31 (1H, d, *J* = 9.0 Hz, H6 **a**), 7.29–6.71 (9H + 9H, m, Ar-H **a** and **b**), 5.74–5.49 (2H + 2H, m, H5 **a** and **b**, H1′ **a** and **b**), 5.11–5.08 (1H, m, H5′′ **a**), 5.00–4.99 (1H, m, H5′′ **b**), 4.23–3.04 (10H + 10H, m, H2′ **a** and **b**, H3′ **a** and **b**, H4′ **a** and **b**, C*H*_2_ 5′ **a** and **b**, OC*H*_3_ **a** and **b**, H4′′ **a** and **b**, H3′′ **a** and **b**), 1.64 (3H, s, 2′′-C*H*_3_ **a**), 1.59 (3H, s, 2′′-C*H*_3_ **b**), 0.83, 0.81 (2 × 9H, s, C(*C*H_3_)_3_ **a**), 0.77, 0.73 (2 × 9H, s, C(*C*H_3_)_3_ **b**), 0.04, 0.03, 0.02, −0.02, −0.04, −0.06, −0.07, −0.11 (8 × 3H, s, Si-C*H*_3_ **a** and **b**); ^13^C NMR (CDCl_3_, 100 MHz) *δ* 177.6, 177.5, 175.3, 173.4, 172.7, 168.5, 167.9, 167.5, 157.9, 156.0, 142.4, 141.4, 136.1, 131.3, 129.2, 128.9, 128.6, 128.5, 126.6, 126.4, 126.2, 125.9, 125.1, 124.6, 124.3, 121.0, 120.9, 120.5, 120.1, 110.2, 109.9, 102.7, 102.3, 90.5, 89.8, 86.8, 85.5, 84.7, 73.9, 73.7, 73.6, 72.9, 72.6, 66.4, 62.5, 62.4, 55.6, 55.4, 54.5, 42.2, 41.9, 41.6, 41.5, 25.8, 25.8, 0.97, −4.5, −4.7, −4.8; *m*/*z* (ESI, +ve ion) 913.4 (MNa)^+^, 891.4 (MH)^+^, 779.4 (M−Ur)^+^, 647.3 (M−2 × TBS–Me)^+^, 474.2 (MNa−uracil–ribose–2 × TBS), 452.2 (MH^+^−uracil–ribose–2 × TBDMS); *v*_max_ 3313, 2931, 2856, 1707 cm^−1^; HRMS (LSIMS, +ve ion) calcd for C_44_H_62_N_6_O_10_Si_2_ (M+H)^+^ 891.4144; found 891.4137.

The 5′-cycloadduct-aminoacyl-2′,3′-*O*-bis(*tert-*butyldimethylsilyl) uridine compound (80 mg) was dissolved in a mixture of DCM, water and TFA in a 4:2:3 ratio, respectively. The mixture was stirred at room temperature for 24 h and was then diluted with water (10 ml) and DCM (10 ml). The aqueous portion was extracted with DCM (3 × 10 ml) and then concentrated in vacuo to yield the crude product. The product was purified by reverse phase HPLC on a Phenomenex Synergi 4u fusion-RP 80A column (250 × 10.0 mm, 4 μ) using a water/ethanol gradient (0–100% ethanol over 35 min, 3.5 ml/min). Isomer **13b**.**1** (32 mg, 0.04 mmol, 46%) was eluted at 19.45 min and **13b**.**2** (29 mg, 0.04 mmol, 42%) was eluted at 21.17 min.

*Compound **13b.1***: Melting point 197–200 °C.^1^H NMR (CD_3_OD, 500 MHz) *δ* 7.61 (1H, d, *J* = 8.0 Hz, H6), 7.55 (1H, d, *J* = 7.5 Hz, Ar-H), 7.53–7.36 (4H, m, Ar-H), 7.23–7.17 (2H, m, Ar-H), 7.12–7.06 (2H, m, Ar-H), 5.75 (1H, d, *J* = 4.5 Hz, H1′), 5.71 (1H, d, *J* = 8.0 Hz, H5), 5.46 (1H, d, *J* = 10.0 Hz, H5′′), 4.38–3.97 (7H, m, H2′, H3′, H4′, H4′′, H3′′, NHC*H*_2_ of glycine linker), 3.83 (3H, s, OC*H*_3_), 3.57 (2H, d, *J* = 5.0 Hz, C*H*_2_ 5′), 1.97 (3H, s, 2′′-C*H*_3_); ^13^C NMR (CD_3_OD, 125 MHz) *δ* 174.3, 173.1, 172.4, 170.0, 169.0, 167.3, 164.7, 157.5, 156.4, 150.8, 141.6, 141.3, 136.5, 131.7, 130.9, 130.7, 128.6, 128.4, 126.2, 126.1, 124.9, 121.2, 120.7, 120.3, 118.3, 110.9, 110.6, 101.7, 101.5, 90.5, 90.1, 82.4, 82.3, 73.4, 70.8, 70.5, 69.4, 67.4, 62.8, 55.1, 55.0, 54.3, 48.2, 48.1, 43.2, 41.4, 40.7, 40.4, 24.9, 19.7; *m*/*z* (ESI, +ve ion) 701.2 (MK)^+^, 685.2 (MNa)^+^, 663.2 (MH)^+^; *v*_max_ 3345, 1668, 1179, 1130 cm^−1^; HRMS (micrOTOF, +ve ion) calcd for C_32_H_35_N_6_O_10_ (M+H)^+^ 663.2409; found 663.2424.

*Compound **13b.2**:* Melting point 160–164 °C. ^1^H NMR (CD_3_OD, 500 MHz) *δ* 7.60 (0.5H, d, *J* = 8.0 Hz, H6), 7.56–7.51 (1H, m, Ar-H), 7.46 (0.5H, d, *J* = 8.0 Hz, H6), 7.41–7.36 (1H, m, Ar-H), 7.23–7.12 (2H, m, Ar-H), 7.12–7.05 (4H, m, Ar-H), 6.99–6.93 (1H, m, Ar-H), 5.78–5.77 (1H, m, H5, H1′), 5.75 (0.5H, d, *J* = 4.5 Hz, H1′), 5.68–5.67 (1.5H, m, H5, H5′′), 4.36 (0.5H, d, *J* = 16.5 Hz, NHC*H*H of glycine linker), 4.25 (1.5H, m, NHC*HH* of glycine linker), 4.20–4.17 (1H, m, H4′′), 4.14 (0.5H, dd, *J* = 4.5, 5.5 Hz, H2′), 4.11 (0.5H, dd, *J* = 4.5, 5.5 Hz, H2′), 4.07–4.05 (1H, m, H3′′), 4.02–3.95 (5H, m, H3′, H4′, OC*H*_3_), 3.53 (2H, m, C*H*_2_ 5′), 1.97 (3H, s, 2′′-C*H*_3_); ^13^C NMR (CD_3_OD, 125 MHz) *δ* 173.1, 172.8, 172.4, 169.1, 168.9, 167.3, 167.1, 164.6, 164.4, 156.4, 150.9, 150.8, 141.7, 141.3, 136.5, 136.5, 130.7, 128.4, 128.3, 126.3, 126.2, 125.0, 124.9, 120.9, 120.7, 120.3, 118.3, 110.6, 101.7, 101.7, 90.7, 90.2, 82.4, 82.1, 73.4, 73.3, 70.9, 70.6, 67.4, 67.4, 62.9, 62.8, 55.0, 52.9, 49.4, 49.2, 41.4, 41.1, 40.5, 40.4, 19.7, 19.6; *m*/*z* (ESI, +ve ion) 701.2 (MK)^+^, 685.3 (MNa)^+^, 663.2 (MH)^+^; *v*_max_ 3309, 1663, 1176, 1139 cm^−1^; HRMS (mircrOTOF, +ve ion) calcd for C_32_H_35_N_6_O_10_ (M+H)^+^ 663.2421; found 663.2409.

*Data for **14a***: White solid (16 mg, coupling 31%, deprotection 74%) isolated as a 1:1 ratio of diastereoisomers **a** and **b**). HPLC retention time 19.77 min. ^1^H NMR (D_2_O, 400 MHz) *δ* 7.70 (1H + 1H, d, *J* = 8.0 Hz, H6 **a** and **b**), 7.55–7.53 (3H + 3H, m, Ar-H **a** and **b**), 7.47–7.45 (2H + 2H, m, Ar-H **a** and **b**), 5.91 (1H + 1H, d, *J* = 8.0 Hz, H5 **a** and **b**), 5.82 (1H + 1H, d, *J* = 4.0 Hz, H1′ **a** and **b**), 5.48 (1H + 1H, d, *J* = 10.0 Hz, H5′′ **a** and **b**), 4.46 (1H + 1H, dd, *J* = 10.0, 10.0 Hz, H4′′ **a** and **b**), 4.39 (1H + 1H, dd, *J* = 4.0, 4.0 Hz, H2′ **a** and **b**), 4.27 (1H + 1H, d, *J* = 10.0 Hz, H3′′ **a** and **b**), 4.21–4.16 (3H + 3H, m, NHC*H*H of glycine linker **a** and **b**, H3′ **a** and **b**, H4′ **a** and **b**), 4.09 (1H + 1H, d, *J* = 17.0 Hz, NHCH*H* of glycine linker **a** and **b**), 3.92 (3.5H + 3.5H, s, CO_2_C*H*_3_ **a** and **b**, C*H*H 5′ **a** and **b**), 3.88–3.87 (0.5H + 0.5H, m, C*H*H 5′ **a** and **b**), 3.56 (1H + 1H, dd, *J* = 7.5, 14.5 Hz, CH*H* 5′ **a** and **b**), 3.35 (3H + 3H, s, CO_2_C*H*_3_ **a** and **b**), 1.84 (3H + 3H, s, 2′′-C*H*_3_ **a** and **b**); ^13^C NMR (D_2_O, 100 MHz) *δ* 170.7, 170.7, 169.7, 166.2, 166.2, 151.3, 141.9, 132.1, 129.9, 129.2, 127.2, 101.8, 90.6, 81.9, 73.4, 70.4, 68.8, 61.3, 53.7, 52.6, 52.5, 50.0, 43.4, 40.9, 17.9; *m*/*z* 642.1 (MK)^+^, 626.2 (MNa)^+^, 604.3 (MH)^+^; *v*_max_ 3293, 2980, 1662 cm^−1^; HRMS calcd for C_27_H_33_N_5_O_11_ (M+H)^+^ 604.2255; found 604.2242.

*Data for **16a***: White solid (62 mg, coupling 33%, deprotection 75%) isolated as 1:1 ratio of diastereoisomers **a** and **b**. HPLC retention time 20.21 min. ^1^H NMR (CD_3_OD, 500 MHz) *δ* 7.65–7.63 (1H + 1H, 2 × overlapping d, *J* = 7.5 and 7.5 Hz, H6 **c** and **d**), 7.42–7.36 (5H + 5H, m, Ar-H **c** and **d**), 5.79 (1H + 1H, d, *J* = 4.5 Hz, H1′ **c** and **d**), 5.77 (1H, d, *J* = 7.5 Hz, H5 **c** or **d**), 5.73 (1H, d, *J* = 8.0 Hz, H5 **c** or **d**), 5.32–5.29 (1H + 1H, 2 × overlapping d, *J* = 7.5 and 7.5 Hz, H5′′ **c** and **d**), 4.33–4.17 (3H + 3H, m, NC*H*_2_ **c** and **d**, H2′ **c** and **d**), 4.05–3.99 (2H + 2H, m, H3′ **c** and **d**, H4′ **c** and **d**), 3.94–3.90 (1H + 1H, m, H4′′ **c** and **d**), 3.81 (1H, d, *J* = 9.5 Hz, H3′′ **c** or **d**), 3.79 (1H, d, *J* = 9.5 Hz, H3′′ **c** or **d**), 3.64–3.56 (2H + 2H, m, C*H*_2_ 5′ **c** and **d**), 3.25 (3H, s, CO_2_CH_3_ **c** or **d**), 3.24 (3H, s, CO_2_C*H*_3_ **c** or **d**), 1.80 (3H + 3H, s, 2′′-C*H*_3_ **c** and **d**); ^13^C NMR (CD_3_OD, 125 MHz) *δ* 176.2, 176.0, 175.4, 175.2, 172.7, 172.6, 168.8, 168.8, 166.2, 152.4, 143.6, 143.3, 134.9, 134.6, 130.2, 130.1, 129.9, 129.8, 128.6, 127.8, 103.2, 103.1, 92.2, 92.1, 83.9, 83.9, 74.7, 74.7, 72.2, 72.1, 68.3, 68.3, 68.0, 54.8, 52.8, 52.7, 49.5, 49.3, 42.5, 42.4, 42.1, 41.9, 21.4, 21.3; *m*/*z* (ESI, +ve ion) 610.1 (MK)^+^, 594.2 (MNa)^+^, 572.2 (MH)^+^; *v*_max_ 3294, 2956, 1665, 1560 cm^−1^; HRMS (LSIMS, +ve ion) calcd for C_26_H_29_N_5_O_10_ (M+H)^+^ 572.1993; found 572.1997. Data for ^13^C, **13e**, **14b**, **15e**, **16b**–**d** given in Supplementary data.

### 4.6 Preparation of N-methylated sarcosine-linked compound **19**

A solution of **2f** (0.43 g, 0.91 mmol) and water (3.40 μl, 0.21 mmol) in dry THF (4 ml) was added dropwise over a period of 20 min to a suspension of sodium hydride (82 mg, 3.40 mmol) in dry THF (6 ml), whilst a temperature of 0 °C was maintained. The mixture was stirred for 10 min and then dimethyl sulfate (0.29 ml, 3.06 mmol) was added dropwise at 0 °C. The mixture was stirred for 3 h and then allowed to warm to room temperature and stirred for a further 21 h. The reaction mixture was quenched by the addition of 30% ammonium hydroxide (5 ml) over a period of 10 min, maintaining a temperature of below 20 °C, and stirring was continued for a period of 1 h. The mixture was diluted with toluene (20 ml) and water (10 ml). The organic phase was separated and washed with water (10 ml) and concentrated in vacuo. The product was purified by flash chromatography (4:1, hexane/ethyl acetate) to yield the N-methylated product as a pure white solid (0.31 g, 0.31 mmol, 38%). Melting point 108–111 °C. ^1^H NMR (CDCl_3_, 400 MHz) *δ* 7.46 (1H, dd, *J* = 1.5, 7.5 Hz, Ar-H), 7.27–7.15 (9H, m, Ar-H), 6.88–6.81 (4H, m, Ar-H), 5.21 (1H, d, *J* = 13.0 Hz, C*H*HPh), 5.17 (1H, d, *J* = 13.0 Hz, CH*H*Ph), 4.46 (1H, d, *J* = 10.0 Hz, H5′′), 3.82 (3H, s, OC*H*_3_), 3.66 (1H, dd, *J* = 8.0, 10.0 Hz, H4′′), 3.36 (1H, d, *J* = 8.0 Hz, H3′′), 2.21 (3H, s, N1′′-C*H*_3_), 1.45 (3H, s, 2′′-C*H*_3_); ^13^C NMR (CDCl_3_, 100 MHz) *δ* 174.9, 173.9, 171.7, 158.1, 136.1, 131.7, 129.0, 128.9, 128.5, 128.4, 128.3, 128.1, 127.1, 126.2, 125.4, 120.6, 110.4, 70.1, 67.1, 62.3, 55.6, 54.9, 46.5, 34.4, 15.6; *m*/*z* (ESI, +ve ion) 523.1 (MK)^+^, 507.2 (MNa)^+^, 485.2 (MH)^+^; *v*_max_ 2958, 2834, 1738, 1712 cm^−1^; HRMS (LSIMS, +ve ion) calcd for C_29_H_28_N_2_O_5_ (M+H)^+^ 485.2076; found 485.2057. This benzyl ester was deprotected by hydrogenation (100% yield), coupled with glycine benzyl ester (49% yield), and further deprotected by hydrogenation (100% yield), as described above for compounds **8a**–**e**, to give compound **18** (32 mg). ^1^H NMR (CD_3_OD, 400 MHz) *δ* 7.61–6.91 (9H, m, Ar-H), 4.69–4.61 (1H, m, H5′′), 4.24–4.06 (1H, m, H2′′), 3.88–3.67 (7H, m, OC*H*_3_, H4′′, H3′′, C*H*_2_ of sarcosine linker), 3.34 (3H, s, NC*H*_3_ of sarcosine linker), 2.18 (3H, s, N1′′-C*H*_3_); ^13^C NMR (CD_3_OD, 100 MHz) *δ* not all quaternary C’s were seen) 177.1, 176.4, 159.3, 133.6, 130.3, 130.1, 129.9, 129.6, 129.6, 129.5, 127.5, 111.4, 69.4, 66.6, 56.0, 51.1, 46.9, 40.3, 36.6; *m*/*z* (ESI, +ve ion) 496.1 (MNa_2_–H)^+^, 490.1 (MK)^+^, 474.2 (MNa)^+^, 452.2 (MH)^+^. Coupling to uridine derivative **12** and deprotection was carried out as described above, to give compound **19** as a white solid (9 mg, 9%). HPLC retention time 20.43 min. A mixture of *cis* and *trans* *N*-methyl amide rotamers was observed by NMR spectroscopy. ^1^H NMR (CD_3_OD, 500 MHz) *δ*7.72–7.30 (7.5H, m, Ar-H, H6), 7.21–7.13 (2H, m, Ar-H), 7.00 (0.5H, m, Ar-H), 5.83–5.73 (1.5H, m, H5, H1′), 5.41–5.38 (0.5H, m, H1′, H5′′), 5.27–5.19 (1H, m, H5′′), 4.66–3.49 (13H, m, H2′, H3′, H4′, C*H*_2_ 5′, H2′′, H5′′, H3′′, OC*H*_3_, N(CH_3_)C*H*_2_), 3.40–3.38 (2H, m, NC*H*_3_ of sarcosine linker), 3.15 (1H, s, NC*H*_3_ of sarcosine linker), 2.94–2.93 (2H, m, N1′′-C*H*_3_), 2.27–2.26 (1H, m, N1′′-C*H*_3_); ^13^C NMR (CD_3_OD, 125 MHz) *δ* 171.3, 165.4, 164.7, 157.9, 157.1, 150.8, 132.4, 131.6, 128.8, 128.7, 128.3, 128.0, 127.5, 126.6, 126.1, 125.9, 122.1, 111.5, 110.0, 101.9, 101.6, 101.5, 91.6, 90.8, 90.4, 89.5, 82.9, 82.6, 82.5, 82.1, 81.9, 73.6, 73.3, 72.9, 72.1, 71.6, 71.1, 70.9, 70.8, 70.5, 68.1, 65.1, 55.6, 55.4, 54.6, 51.6, 51.5, 51.3, 45.7, 44.6, 41.2, 40.7, 40.4, 40.3, 38.9, 38.6, 35.6, 35.3, 34.9, 34.6; *m*/*z* (ESI, +ve ion) 715.1 (MK)^+^, 699.2 (MNa)^+^, 677.3 (MH)^+^; *v*_max_ 3350, 1713, 1661, 1167, 1130 cm^−1^. HRMS (MicrOTOF, +ve ion) calcd for C_33_H_37_N_6_O_10_ (M+H)^+^ 677.2566; found 677.2580.

### 4.7 Preparation of proline-linked compounds

Cycloadducts **3b**, **5b**, **and** **5b** were coupled with l-proline benzyl ester, and deprotected by hydrogenation, using the methods described above for compound **8**, to give the l-prolyl adducts. These compound were coupled with 5′-amino-uridine derivative **12**, and deprotected as described above for compounds **13**–**16**.

*Data for **20a***: The product was purified by HPLC using a water/methanol gradient and **20a** was eluted at 17.89 min and concentrated to yield a white solid (25 mg; l-Pro coupling 91%, deprotection 100%, uridine coupling/deprotection 15%) isolated as a 1:1 ratio of diastereoisomers **a** and **b**. ^1^H NMR (CD_3_OD, 500 MHz) *δ* 7.66 (1H + 1H, d, *J* = 8.0 Hz, H6 **a** and **b**), 7.50–7.39 (5H + 5H, m, Ar-H **a** and **b**), 7.25–7.23 (2H + 2H, m, Ar-H **a** and **b**), 7.13–7.07 (2H + 2H, m, Ar-H **a** and **b**), 5.76–5.70 (2H + 2H, m, H1′ **a** and **b**, H5 **a** and **b**), 5.53 (1H + 1H, d, *J* = 10.0 Hz, H5′′ **a** and **b**), 4.44 (1H + 1H, dd, *J* = 7.5, 9.5 Hz, NC*H* of proline linker **a** and **b**), 4.29–4.16 (3H + 3H, m, H2′ **a** and **b**, H3′ **a** and **b**, H4′′ **a** and **b**), 4.15–3.95 (3H + 3H, m, NC*H*_2_ of proline linker **a** and **b**, H4′ **a** and **b**), 3.77 (1H + 1H, m, H3′′ **a** and **b**), 3.74 (3H + 3H, s, OC*H*_3_ **a** and **b**), 3.60–3.50 (2H + 2H, m, C*H*_2_ 5′ **a** and **b**), 2.43–2.38 (1H + 1H, m, C*H*_2_ of proline linker **a** and **b**), 2.17–2.12 (2H + 2H, m, C*H*_2_ of proline linker **a** and **b**), 2.10 (3H + 3H, s, 2′′-C*H*_3_ **a** and **b**), 1.94–1.86 (1H + 1H, m, C*H*_2_ of proline linker **a** and **b**); ^13^C NMR (CD_3_OD, 125 MHz) *δ*174.4, 174.3, 173.3, 173.1, 168.7, 166.1, 158.8, 152.4, 152.3, 143.6, 143.4, 133.2, 133.0, 132.7, 130.1, 129.9, 127.3, 123.1, 117.9, 112.8, 103.1, 103.0, 92.8, 83.9, 74.6, 72.4, 72.3, 64.7, 62.0, 56.9, 53.6, 51.6, 42.4, 30.7, 26.9, 21.3; *m*/*z* (ESI, +ve ion) 725.3 (MNa)^+^, 703.3 (MH)^+^; HRMS (ESI, +ve ion) calcd for C_35_H_38_N_6_O_10_ (M+H)^+^ 703.2722; found 703.2741. Data for **20b**–**d** given in Supplementary data.

### 4.8 Preparation of ester-linked compounds **25** and **26**

Cycloadduct isopropyl ester **2g** (0.20 g, 0.47 mmol) was dissolved in dry THF (5 ml) and cooled to −78 °C under a nitrogen atmosphere. A 1 M solution of DIBAL-H in hexane (1.00 ml, 0.99 mmol) was added dropwise. The mixture was stirred at −78 °C for 1 h and was then quenched by the cautious addition of methanol (1 ml). The THF was removed in vacuo and the residue was dissolved in diethyl ether (10 ml). A saturated solution of potassium sodium tartrate (10 ml) was then added and the mixture was left to stir overnight until two layers had separated. The organic portion was washed with brine (2 × 10 ml), dried (MgSO_4_) and concentrated in vacuo. The product was purified by flash chromatography (3:1, hexane/ethyl acetate) to yield alcohol **22** as a white crystalline solid (44 mg, 0.12 mmol, 26%). Melting point 104–107 °C. ^1^H NMR (CDCl_3_, 300 MHz) *δ* 7.78 (1H, d, *J* = 7.5 Hz, Ar-H), 7.53–7.50 (2H, m, Ar-H), 7.33–7.23 (3H, m, Ar-H), 7.16–7.11 (1H, m, Ar-H), 7.06–7.01 (1H, m, Ar-H), 6.86 (1H, d, *J* = 8.5 Hz, Ar-H), 5.04 (1H, dd, *J* = 2.0, 3.5 Hz), 4.90 (1H, d, *J* = 12.0 Hz), 4.67 (1H, br d, *J* = 9.5 Hz), 4.49 (1H, br d, *J* = 12.0 Hz), 3.81 (3H, s, OC*H*_3_), 3.72 (1H, dd, *J* = 3.5, 5.5 Hz), 3.01 (1H, dd, *J* = 2.0, 5.5 Hz), 1.92 (2H, br s, N*H*_2_), 1.39 (3H, s, 2′′-C*H*_3_); ^13^C NMR (CDCl_3_, 75 MHz) *δ* 171.9, 156.2, 137.2, 128.9, 128.7, 127.9, 127.0, 125.4, 120.9, 120.5, 109.2, 95.1, 88.1, 65.1, 57.1, 56.5, 55.2, 50.4, 19.5; *m*/*z* (ESI, +ve ion) 405.1 (MK)^+^, 389.1(MNa)^+^, 367.1 (MH)^+^, 349.1 (M–OH)^+^; *v*_max_ 3343, 2924, 1708, 1045, 753 cm^−1^. HRMS (LSIMS, +ve ion) calcd for C_21_H_22_N_2_O_4_ (M+H)^+^ 367.1645; found 367.1658.

### 4.9 Malonyl diester **25**

5′-Malonyl 2′,3′-isopropylidene uridine **23** was prepared by EDCI coupling of monobenzyl malonate with 2′,3′-isopropylidene uridine (23% yield), followed by hydrogenation (100% yield). Compound **23** (87 mg, 0.23 mmol) was dissolved in dry THF (5 ml) and cooled to 0 °C. HATU (128 mg, 0.34 mmol) was added and the mixture was stirred at 0 °C for 10 min. DIPEA (60 mg, 0.46 mmol) was added followed by alcohol **22** (100 mg, 0.26 mmol). The mixture was stirred at 0 °C for 4 h and was then allowed to warm to room temperature and stirred for 24 h. The solvent was removed in vacuo and the residue was partitioned between ethyl acetate and water. The organic portion was washed with saturated sodium bicarbonate (10 ml), brine (2 × 10 ml) and then dried (MgSO_4_) and concentrated in vacuo. The crude yellow oil (90 mg) was dissolved in DCM (2 ml). Sixty-six percentage of TFA (3 ml) was then added and the mixture was stirred at room temperature for 24 h. DCM (5 ml) and water (5 ml) was then added and the mixture was allowed to separate. The aqueous phase was extracted with DCM (2 × 5 ml) and then concentrated in vacuo. The residue was then purified by HPLC using a water/methanol gradient and **25** was eluted at 24.80 min and concentrated to yield a white solid (29 mg, 0.11 mmol, 48%), isolated as a 1:1 ratio of diastereoisomers **a** and **b**. ^1^H NMR (CD_3_OD, 400 MHz) *δ* 9.97 (1H + 1H, s, NH3 **a** and **b**), 7.70 (1H, d, *J* = 8.0 Hz, H6 **a** or **b**), 7.60 (1H, d, *J* = 8.0 Hz, H6 **a** or **b**), 7.40–7.34 (2H + 2H, m, Ar-H **a** and **b**), 7.22–7.12 (5H + 5H, m, Ar-H **a** and **b**), 6.99–6.94 (1H + 1H, m, Ar-H **a** and **b**), 6.72–6.69 (1H + 1H, m, Ar-H **a** and **b**), 5.98 (1H, d, *J* = 10.5 Hz, H5′′ **a** or **b**), 5.96 (1H, d, *J* = 10.5 Hz, H5′′ **a** or **b**), 5.89–5.87 (1H + 1H, m, H1′ **a** and **b**), 5.76 (1H, d, *J* = 8.0 Hz, H5 **a** or **b**), 5.66 (1H, d, *J* = 8.0 Hz, H5 **a** or **b**), 4.78 (1H + 1H, d, *J* = 4.0 Hz), 4.36–4.06 (5H + 5H, m, H2′ **a** and **b**, H3′ **a** and **b**, H4′ **a** and **b**, CH_2_ 5′ **a** and **b**), 4.02 (3H, s, OC*H*_3_ **a** or **b**), 4.00 (3H, s, OC*H*_3_ **a** or **b**), 3.70 (1H + 1H, d, *J* = *7.5* Hz, H3′′ **a** and **b**), 3.65 (1H + 1H, ddd, *J* = 3.0, 7.5, 10.5 Hz, H4′′ **a** and **b**), 3.25 (1H + 1H, dd, *J* = 3.0, 16.0 Hz, C*H*HOCO **a** and **b**), 3.02 (1H + 1H, dd, *J* = 3.0, 16.0 Hz, CH*H*OCO **a** and **b**), 1.71 (3H, s, 2′′-C*H*_3_ **a** or **b**), 1.69 (3H, s, 2′′-C*H*_3_ **a** or **b**); ^13^C NMR (CD_3_OD, 125 MHz) *δ* 199.3, 199.2, 171.5, 169.0, 167.7, 166.0, 157.8, 152.3, 142.6, 142.3, 137.4, 131.1, 129.6, 128.5, 128.1, 126.1, 125.8, 124.7, 122.7, 112.3, 111.7, 103.2, 91.2, 91.1, 86.7, 86.7, 83.0, 82.9, 75.1, 75.0, 74.9, 72.1, 71.1, 65.6, 65.5, 59.2, 59.0, 57.3, 57.3, 56.5, 56.3, 48.9, 20.0; *m*/*z* (micrOTOF, +ve ion) 701.2 (MNa)^+^; *v*_max_ 3392, 1679, 1399 cm^−1^; HRMS (micrOTOF, +ve ion) calcd for C_33_H_34_N_4_O_12_Na, 701.2065; found 701.2069.

### 4.10 Succinyl diester **26**

5′-Succinyl 2′,3′-isopropylidene uridine **24** was prepared by reaction of succinic anhydride with 2′,3′-isopropylidene uridine (40% yield). Compound **24** (35 mg, 0.09 mmol) was dissolved in dry THF (5 ml) and cooled to 0 °C. HATU (45 mg, 0.12 mmol) was then added. The mixture was stirred for 10 min at 0 °C and then DIPEA (23 mg, 0.18 mmol) was added followed by alcohol **19** (40 mg, 0.10 mmol). The mixture was stirred at 0 °C for 2 h and then allowed to warm to room temperature and stirred for a further 48 h. The solvent was removed in vacuo and the residue was dissolved in ethyl acetate (10 ml) and water (10 ml). The organic phase was washed with brine (3 × 10 ml), dried (MgSO_4_) and concentrated in vacuo to yield the crude product. The crude product was dissolved in a mixture of DCM, water and TFA in a 4:2:3 ratio, respectively. The mixture was stirred at room temperature for 24 h and was then diluted with water (10 ml) and DCM (10 ml). The aqueous portion was extracted with DCM (3 × 10 ml) and then concentrated in vacuo to yield the crude product. The product was purified by HPLC using a water/methanol gradient. Compound **26** was eluted at 22.49 min and concentrated to yield a white solid (8 mg, 0.01 mmol, 11%) as a 1:1 ratio of diastereoisomers **a** and **b**. ^1^H NMR (CD_3_OD, 500 MHz) *δ* 7.72–7.66 (3H + 3H, m, Ar-H **a** and **b**, H6 **a** and **b**), 7.53–7.43 (4H + 4H, m, Ar-H **a** and **b**), 7.31 (1H + 1H, dt, *J* = 1.5, 7.5 Hz, Ar-H **a** and **b**), 7.16 (1H + 1H, d, *J* = 7.5 Hz, Ar-H **a** and **b**), 7.12 (1H + 1H, d, *J* = 8.5 Hz, Ar-H **a** and **b**), 6.42 (1H + 1H, s), 6.00 (1H + 1H, dd, *J* = 6.5, 6.5 Hz), 5.86–5.85 (1H + 1H, m, H1′ **a** and **b**), 5.79–5.77 (1H + 1H, m, H5 **a** and **b**), 5.53 (1H + 1H, d, *J* = 6.5 Hz, H5′′ **a** and **b**), 4.50–4.43 (1H + 1H, m, C*H*H 5′ **a** and **b**), 4.36–4.31 (1H + 1H, m, CH*H* 5′ **a** and **b**), 4.24 (1H + 1H, dd, *J* = 4.5, 4.5 Hz, H2′ **a** and **b**), 4.18–4.14 (2H + 2H, m, H3′ **a** and **b**, H4′ **a** and **b**), 3.87–3.80 (2H + 2H, m, H4′′ **a** and **b**, H3′′ **a** and **b**), 3.77 (3H + 3H, s, OC*H*_3_ **a** and **b**), 2.87–2.80 (4H + 4H, m, C*H*_2_ 1′′′ **a** and **b**, C*H*_2_ 2′′′ **a** and **b**), 1.86 (3H + 3H, s, 2′′-C*H*_3_ **a** and **b**); ^13^C NMR (CD_3_OD, 125 MHz) *δ* 172.2, 170.4, 170.4, 170.1, 164.7, 157.9, 150.8, 141.1, 137.2, 131.5, 130.0, 128.7, 126.4, 122.2, 122.1, 121.5, 116.7, 111.5, 101.5, 98.3, 90.8, 90.4, 90.3, 81.5, 73.6, 73.6, 69.8, 64.7, 63.6, 55.3, 50.5, 49.2, 28.4, 28.1, 17.2; *m*/*z* (ESI, +ve ion) 731.1 (MK)^+^, 715.2 (MNa)^+^, 693.2 (MH)^+^; *v*_max_ 2972, 1674, 1130 cm^−1^; HRMS (ESI, +ve ion) calcd for C_34_H_36_N_4_O_12_ (M+H)^+^ 693.2402; found 693.2412.

### 4.11 Purification of recombinant *E. coli* MurG

Freshly transformed *E. coli* C43 expressing *E. coli* *murG* from a pET3a vector were grown in 6L of LB medium supplemented with 100 μg/ml ampicillin, and induced with IPTG (1 mM) at OD_600_ 0.5. The induced cell culture was grown for another 3 h and then the cells were centrifuged at 10,000 g for 10 min. The cell pellets were resuspended in 50 mM Tris–HCl pH 7.9 (50 ml) containing 2.5 mg/ml lysozyme, PMSF (0.2 mM), leupepsin (0.2 mM) and pepstatin (0.02 mM) were added and the suspension was sonicated. The suspension was centrifuged at 10,000*g* for 20 min and then the supernatant was transferred to fresh tubes and centrifuged at 50,000*g* for 1 h. The pellet was resuspended in 50 mM HEPES pH 7.6 (15 ml) containing 2 mM MgCl_2_, 0.5% CHAPS, 10% glycerol, 0.5 M NaCl and 5 mM imidazole and stirred at 4 °C for 1 h. The suspension was then centrifuged again at 50,000*g* for 1 h, and the supernatant was retained. The pellet was suspended again in 15 ml of 50 mM HEPES pH 7.6, 2 mM MgCl_2_, 0.5% CHAPS, 10% glycerol, 0.5 M NaCl and 5 mM imidazole and stirred at 4 °C for 1 h. The suspension was then centrifuged at 50,000*g* for 1 h and the supernatant was pooled with the supernatant from the previous centrifugation. The supernatant was loaded onto a Ni affinity HisTrap fast flow column (5 ml) equilibrated with 50 mM HEPES pH 7.6, 2 mM MgCl_2_, 0.5% CHAPS, 10% glycerol, 0.5 M NaCl and 5 mM imidazole (Buffer A). The column was washed with 30 ml of Buffer A. The column was eluted with buffer solutions based upon Buffer A containing 50 mM imidazole (30 ml) and 250 mM imidazole (50 ml). MurG eluted with the 250 mM wash. The material was then loaded onto a Superdex 200 HR 16/60 column (Pharmacia Biotech) at a flow rate of 2 ml/min of 20 mM Tris–HCl pH 7.9, 150 mM NaCl, 50 mM EDTA, 4 mM DTT and 0.5% CHAPS. The protein eluted as a symmetrical peak at 180–250 ml. Fractions containing MurG were pooled and concentrated using Centricon tubes to a final volume of 4.5 ml. The protein concentration was estimated to be 4.15 mg/ml and the protein was seen to be homogeneous by a Coomassie blue-stained SDS–polyacrylamide gel. The purified enzyme was stored at −20 °C and was stable for at least three months.

### 4.12 Kinetic assays of *E. coli* MurG

A preparation of solubilised translocase MraY was prepared from *Micrococcus flavus* membranes (100 μl of 19 mg protein/ml stock) added to 150 μl of solubilisation buffer (50 mM Tris–HCl pH 7.5, 1 mM MgCl_2_, 2 mM 2-mercaptoethanol, 0.5% CHAPS). The mixture was shaken at 4 °C for 30 min and was then centrifuged at 13,000 rpm for 30 min. The protein concentration of the supernatant was estimated by Biorad assay to be 1.5 mg/ml and was used directly in the radiochemical assay. Freshly solubilised MraY (12.5 μl) was added directly to undecaprenyl phosphate (0.25 μg). 12.5 μl of buffer (400 mM Tris–HCl pH 7.5, 100 mM MgCl_2_) was added, followed by water (9 μl), UDP-MurNAc-pentapeptide solution (5 μl, 1 mM), MurG (1 μl, 110 μg protein/ml) and 5 μl of aqueous inhibitor solution. The mixture was incubated at 35 °C for 15 min, and then 5 μl of UDP-[^3^H]GlcNAc (10 μM, 500 mCi/mmol) was added, and the mixture was incubated for a further 15 min. The reaction was stopped by the addition of 50 μl of 6 M pyridinium acetate pH 4.6. Hundred microlitres of *n*-butanol were added, and the layers were mixed and separated by centrifugation. Hundred microlitres of the top *n*-butanol phase were removed and counted for radioactivity.

### 4.13 Antibacterial testing

The following procedure was performed using *P. putida* (ATCC 33015), *M. luteus* (ATCC 13513), *E. coli* BL21 and *B. subtilis* W23. A single colony of the bacterial strain was picked from an LB agar plate and grown in 10 ml of sterile LB medium overnight at 37 °C. 1 ml of this culture was then used to inoculate 50 ml of sterile LB medium at 2%. Hundred microlitres of this 2% culture was pipetted into each well of a 96-well plate. Hundred microlitres of sterile water or sterile aqueous inhibitor solution (200 μg/ml or 1 mg/ml) were then pipetted into each well and the 96-well plates were incubated at 37 °C. Bacterial growth was monitored by measuring the OD at 595 nm at 3, 6 and 24 h.

### 4.14 Preparation of *M. smegmatis* membrane and cell wall enzyme fractions

Liquid cultures of *Mycobacterium smegmatis* mc^2^155 were grown at 37 °C in Luria Bertoni (LB) broth medium (Difco) supplemented with 0.05% Tween 80, biomass harvested, washed with phosphate buffered saline (PBS) and stored at −20 °C until further use. *M. smegmatis* cells (10 g wet weight) were washed and re-suspended in 30 ml of buffer A, containing 50 mM MOPS (adjusted to pH 8.0 with KOH), 5 mM β-mercaptoethanol and 10 mM MgCl_2_ at 4 °C and subjected to probe sonication (Soniprep 150, MSE Sanyo Gallenkamp, Crawley, Sussex, UK; 1 cm probe) for a total time of 10 min in 60 s pulses and 90 s cooling intervals between pulses. The sonicate centrifuged at 27,000*g* for 60 min at 4 °C. The resulting mycobacterial cell wall pellets were re-suspended in buffer A. Percoll (Pharmacia, Sweden) was added to yield a 60% suspension and centrifuged at 27,000*g* for 1 h at 4 °C. The upper, particulate diffuse cell wall enzymatically active (P60) band was collected and washed three times with buffer A and re-suspended in buffer A at a final protein concentration of 10 mg/ml. Membrane fractions were obtained by centrifugation of the 27,000*g* supernatant at 100,000*g* for 1 h at 4 °C. The supernatant was carefully removed and the membranes gently re-suspended in buffer A at a protein concentration of 20 mg/ml. Protein concentrations were determined using the BCA Protein Assay Reagent kit (Pierce Europe, Oud-Beijerland, Netherlands).

### 4.15 Galactosyltransferase assay

The reaction mixtures for assessing [^14^C]Gal incorporation consisted of UDP-[U-^14^C]Gal (Amersham Pharmacia Biotech, 327 mCi/mmol, 0.25 μCi, 10 μl), Gal*f*(β1→6)Gal*f*-*O*-C_8_ acceptor (0.4 mM), ATP (1 mM, 5 μl), NADH (100 mM, 8 μl), membranes (250 μg, 12.5 μl) and the cell wall fraction (250 μg, 25 μl) in a final reaction volume of 80 μl. The reaction mixtures were then incubated at 37 °C for 1 h. A CHCl_3_/CH_3_OH (1:1, 533 μl) solution was then added to the incubation tubes and the entire contents centrifuged at 18,000*g*. The supernatant was recovered and dried under a stream of argon and re-suspended in C_2_H_5_OH/H_2_O (1:1, 1 ml) and loaded onto a pre-equilibrated (C_2_H_5_OH/H_2_O [1:1]) 1 ml Whatmann strong anion exchange (SAX) cartridge which was washed with 3 ml of ethanol. The eluate was dried and the resulting products partitioned between the two phases arising from a mixture of *n*-butanol (3 ml) and H_2_0 (3 ml). The resulting organic phase was recovered following centrifugation at 3,500*g* and the aqueous phase was again extracted twice with 3 ml of *n*-butanol saturated water, the pooled extracts were back-washed twice with water saturated with *n*-butanol (3 ml). The *n*-butanol–saturated water fraction was dried and re-suspended in 200 μl of *n*-butanol. The total cpm of radiolabeled material extractable into the *n*-butanol phase was measured by scintillation counting using 10% of the labelled material and 10 ml of EcoScintA (National Diagnostics, Atlanta). The incorporation of [^14^C]Gal was determined by subtracting counts present in control assays (incubation of the reaction components in the absence of the compounds). Another 10% of the labelled material was subjected to thin-layer chromatography (TLC) in CHCl_3_/CH_3_OH/NH_4_OH/H_2_O (65:25:0.5:3.6) on aluminium backed Silica Gel 60 F_254_ plates (E. Merck, Darmstadt, Germany). Autoradiograms were obtained by exposing TLCs to X-ray film (Kodak X-Omat) for 4–5 days. Competition based experiments were performed by mixing compounds together at various concentrations (acceptor, 0.4 mM with inhibitors at 0.5–1.0 mM) followed by thin-layer chromatography/autoradiography as described earlier to determine the extent of product formation.

## Figures and Tables

**Table 1:** Percentage inhibition of *Escherichia coli* MurG and *Mycobacterium tuberculosis* galactosyl transferase by compounds **13**–**25**, at 1 mM concentration

**Figure:**
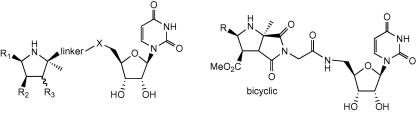

	R_1_	R_2_	R_3_	linker	*E. coli* MurG	*Mt* GTase
**13a**	Ph	*N*-Phenyl succinimide		Gly	NT	NT
**13b**	2-MeOPh	*N*-Phenyl succinimide		Gly	85	0
**13c**	2,3-(MeO)_2_Ph	*N*-Phenyl succinimide		Gly	0	0
**13e**	Ethane-1,2-diol	*N*-Phenyl succinimide		Gly	0	0
**14a**	Ph	-CO_2_Me	α–CO_2_Me	Gly	0	NT
**14b**	2-MeOPh	-CO_2_Me	α–CO_2_Me	Gly	32	0
**15b**	2-MeOPh	-CO_2_Me	Bicyclic	Gly	22	0
**15e**	Ethane-1,2-diol	-CO_2_Me	Bicyclic	Gly	27	NT
**16a**	Ph	-CO_2_Me	Bicyclic	Gly	0	80
**16b**	2-MeOPh	-CO_2_Me	Bicyclic	Gly	0	0
**16c**	2,3-(MeO)_2_Ph	-CO_2_Me	Bicyclic	Gly	0	NT
**16d**	3,4-(MeO)_2_Ph	-CO_2_Me	Bicyclic	Gly	26	0
**19**	2-MeOPh	*N*-Phenyl succinimide		Sar	31	NT
**20a**	2-MeOPh	*N*-Phenyl succinimide		Pro	0	0
**20b**	2-MeOPh	-CO_2_Me	β–CO_2_Me	Pro	0	0
**20c**	2-MeOPh	-CO_2_Me	α–CO_2_Me	Pro	22	0
**20d**	Ethane-1,2-diol	*N*-Ethyl succinimide		Pro	0	0
**24**	2-MeOPh	*N*-Phenyl succinimide		C3	17	0
**25**	2-MeOPh	*N*-Phenyl succinimide		C4	15	0

Linkers: glycine (Gly), sarcosine (Sar), proline (Pro), C-3 diester (C3), C-4 diester (C4). NT, not tested. Assays carried out as described in Section 4.

## Supplementary data

Supplementary data Spectroscopic data for synthetic compounds.
