# Supplementary material for: Inhibition of Escherichia coli glycosyltransferase MurG and Mycobacterium tuberculosis Gal transferase by uridine-linked transition state mimics
Source: Bioorg Med Chem. 2010 Apr 1;18(7):2651–63. doi: 10.1016/j.bmc.2010.02.026 (PMC3034214; doi:10.1016/j.bmc.2010.02.026)
Supplement: Supplementary data — Spectroscopic data for synthetic compounds. [file mmc1.doc]

**Inhibition of *Escherichia coli* Glycosyltransferase MurG**

**and *Mycobacterium tuberculosis* Gal transferase by**

Uridine-linked Transition State Mimics

Amy E. Trunkfielda, Sudagar S. Gurchab, Gurdyal S. Besrab, and Timothy D.H. Bugga*

# Supplementary Data.

*Spectroscopic data for synthetic compounds*.

Data for **2a**: Melting point 183 – 184 ˚C. 1H NMR (CD3CN, 400 MHz)  7.49 – 7.09 (15H, m, Ar-H), 5.27 (1H, d, *J = 12.0 Hz*, C*H*HPh), 5.18 (1H, d, *J = 12.0 Hz*, CH*H*Ph), 5.02 – 4.98 (1H, m, H5’’), 3.86 (1H, dd, *J = 9.5, 9.5 Hz*, H4’’), 3.65 (1H, d, *J = 9.5 Hz*, H3’’), 2.84 - 2.83 (1H, m, NH1’’), 1.69 (3H, s, 2’’-C*H*3); 13C NMR(CDCl3­, 100MHz)  (not all quarternary C’s seen) 138.7, 136.2, 128.8, 128.3, 128.1, 127.9, 127.9, 127.6, 127.3, 126.6, 66.9, 66.5, 61.1, 55.2, 49.4, 22.7; m/z(ESI, +ve ion) 479.0 (MK)+, 463.1 (MNa)+, 441.2 (MH)+; max 3335, 1726, 1702 cm-1; HRMS calculated for C27H24N2O4 (M+H+) 441.1814; Found, 441.1818.

Data for **2b**: white solid(0.24 g, 0.52 mmol, 62%). Melting point 153 – 155 ˚C. 1H NMR(CDCl3, 400 MHz)  7.48 – 7.25 (10H, m, Ar-H), 7.08 (2H, d, *J = 8.5 Hz*, Ar-H), 6.95 (1H, dd, *J = 7.5, 7.5 Hz*, Ar-H), 6.85 (1H, d, *J = 8.5 Hz*, Ar-H), 5.33 (1H, d, *J = 12.0 Hz*, C*H*HPh), 5.22 (1H, d, *J = 12.0 Hz*, CH*H*Ph), 4.92 (1H, br dd, *J = 8.5, 9.5 Hz*, H5’’), 3.80 (1H, dd, *J = 8.5, 8.5 Hz*, H4’’), 3.69 (3H, s, CO2C*H*3), 3.51 (1H, br s, NH1’’), 3.46 (1H, d, *J = 8.5 Hz*, H3’’), 1.68 (3H, s, 2’’-C*H*3); 13C NMR (CDCl3, 100 MHz)  175.1, 172.2, 157.6, 135.5, 131.8, 129.4, 128.9, 128.6, 128.6, 128.4, 128.3, 127.5, 126.1, 124.3, 120.7, 110.5, 68.6, 67.9, 56.5, 55.3, 55.3, 49.6, 24.1; m/z(ESI, +ve ion) 509.1 (MK)+, 493.1 (MNa)+, 471.2 (MH)+; *vmax* 3344, 2991, 2972, 2943, 1737, 1704 cm-1; HRMS (LSIMS, +ve ion) Calculated for C28H26N2O5 (M+H)+ 471.1920. Found 471.1926.

Data for **2c**: white solid (0.44 g, 0.87 mmol, 45%). Melting point 87 – 90 ˚C. 1H NMR (CDCl3, 400 MHz)  7.43 – 7.29 (8H, m, Ar-H), 7.11 – 7.08 (2H, m, Ar-H), 7.04 – 6.99 (2H, m, Ar-H), 6.99 – 6.86 (1H, m, Ar-H), 5.33 (1H, d, *J = 12.0 Hz*, C*H*HPh), 5.21 (1H, d, *J = 12.0 Hz*, CH*H*Ph), 5.08 (1H, dd, *J = 9.0, 9.5 Hz*, H5’’), 3.97 (3H, s, OC­*H*3), 3.85 (3H, s, OC­*H*3), 3.75 (1H, dd, *J = 9.0, 8.0 Hz*, H4’’), 3.48 (1H, d, *J = 8.0 Hz*, H3’’), 2.96 (1H, d, *J = 9.5 Hz*, NH1’’), 1.69 (3H, s, 2’’-C*H*3); 13C NMR (CDCl3, 100 MHz)  174.9, 173.1, 172.2, 152.3, 146.9, 135.6, 131.8, 129.9, 129.0, 128.5, 128.4, 128.4, 128.3, 126.3, 123.8, 118.4, 112.7, 68.1, 67.8, 60.7, 58.2, 56.1, 55.7, 49.6, 23.9; m/z (ESI, +ve ion) 539.1 (MK)+, 523.1 (MNa)+, 501.2 (MH)+; *vmax*3332, 2939, 2836, 1708 cm-1; HRMS (LSIMS, +ve ion) Calculated for C29H28N2O6 (M+H)+ 501.2026. Found 501.2035.

Data for **2d**: white amorphous solid (0.22 g, 0.44 mmol, 44%). 1H NMR (CDCl3, 400 MHz)  7.44 – 7.29 (8H, m, Ar-H), 7.09 (2H, d, *J = 8.5 Hz*, Ar-H), 6.98 – 6.92 (2H, m, Ar-H), 6.83 (1H, d, *J = 8.5 Hz*, Ar-H), 5.35 (1H, d, *J = 12.5 Hz*, C*H*HPh), 5.21 (1H, d, *J = 12.5 Hz*, CH*H*Ph), 4.85 (1H, dd, *J = 7.0, 9.0 Hz*, H5’’), 3.85 (3H, s, OC*H*3), 3.74 (3H, s, OC*H*3), 3.65 (1H, dd, *J = 7.5, 9.0 Hz*, H4’’), 3.47 (1H, d, *J = 7.5 Hz*, H3’’), 2.56 (1H, d, *J = 7.0 Hz*, NH1’’), 1.66 (3H, s, 2’’-C*H*3); 13C NMR (CDCl3, 100 MHz)  174.5, 173.6, 172.2, 149.0, 148.9, 135.8, 131.6, 129.5, 129.0, 128.5, 128.5, 128.4, 128.3, 125.9, 119.5, 110.9, 110.1, 67.7, 67.4, 62.2, 55.8, 55.7, 55.5, 49.9, 23.9; m/z (ESI, +ve ion) 539.1 (MK)+, 523.1 (MNa)+, 501.2 (MH)+, 273.1 (MH - Ph(OMe)2 – CH2Ph)+; *vmax*3337, 2936, 2835, 1708 cm-1. HRMS (LSIMS, +ve ion) Calculated for C29H28N2O6 (M+H)+ 501.2026. Found 501.2039.

Data for **2e**: white solid(0.44 g, 0.88 mmol, 96 %). 1H NMR (CDCl3, 400 MHz)  7.47 – 7.24 (10H, m, Ar-H), 5.26 (1H, d, *J = 12.0 Hz, CH*HPh), 5.11 (1H, d, *J = 12.0 Hz*, CH*H*Ph), 4.67 (1H, dt, *J = 3.0, 7.0 Hz*, H1’’’), 4.19 (1H, dd, *J = 7.0, 7.0 Hz*, C*H*H2’’’), 3.97 (1H, dd, *J = 7.0, 7.0* *Hz*, CH*H*2’’’), 3.76 – 3.67 (1H, m, H5’’), 3.46 – 3.44 (2H, m, H4’’ and H3’’), 3.24 (1H, d, *J = 11.5 Hz*, NH1’’), 1.54 (3H, s, 2’’-C*H*3), 1.29 and 1.28 (3H + 3H, 2 x s, C(C*H*3)2); 13C NMR (CDCl3, 100MHz)  175.0, 174.5, 171.4, 135.2, 131.9, 129.0, 128.8, 128.7, 128.5, 128.4, 126.3, 110.0, 73.3, 68.7, 68.0, 67.5, 61.8, 57.7, 48.2, 26.1, 25.0, 24.2; m/z(ESI, +ve ion) 503.1 (MK)+, 487.2 (MNa)+, 465.2 (MH)+, 436.2 (MH2 - 2 x CH3)+, 407.2 (MH - OC(CH3)2)+; max 2986, 1736, 1707 cm-1. HRMS(LSIMS, +ve ion) Calculated for C26H28N2O6 (M+H)+ 464.1947; found 464.1936.

Data for **2f**: yellow gum (1.08 g, 2.38 mmol, 86%). Melting point 96 – 99 ˚C. 1H NMR (CDCl3, 400 MHz)  7.28 – 7.14 (10H, m, Ar-H), 7.02 (2H, d, *J = 7.5 Hz*, Ar-H), 6.84 (1H, dd, *J = 7.5, 7.5 Hz*, Ar-H), 6.76 (1H, d, *J = 8.5 Hz*, Ar-H), 5.23 (1H, d, *J = 12.0 Hz*, C*H*HPh), 5.15 (1H, d, *J = 12.0 Hz*, CH*H*Ph), 4.56 (1H, d, *J = 7.0 Hz*, H5’’), 4.02 (1H, d, *J = 7.0 Hz*, H2’’), 3.66 (3H, s, OC*H*3), 3.60 – 3.55 (2H, m, H3’’, H4’’), 2.75 (1H, br s, NH1’’); 13C NMR (CDCl3, 100 MHz)  175.3, 173.9, 169.9, 157.4, 135.5, 131.9, 129.3, 129.1, 128.9, 128.8, 128.6, 128.5, 127.1, 126.1, 124.7, 120.7, 110.4, 67.5, 62.7, 61.0, 55.5, 49.0, 48.4; m/z (ESI, +ve ion) 495.0 (MK)+, 479.1 (MNa)+, 457.2 (MH)+; *vmax* 3065, 3020, 2928, 2838, 1742, 1707 cm-1; HRMS (microTOF, +ve ion) Calculated for C27H24N2O5 (M+H)+ 457.1779. Found 457.1761.

Data for **2g**: yellow gum, (0.36 g, 0.89 mmol, 32%). 1H NMR (CDCl3, 400 MHz)  7.42 – 7.30 (5H, m, Ar-H), 5.29 (1H, d, *J = 12.0 Hz*, CH*H*Ph), 5.17 (1H, d, *J = 12.0 Hz*, C*H*HPh), 4.56 (1H, dt, *J = 3.5, 7.0 Hz*, H1’’’), 4.19 (1H, dd, *J = 7.0, 8.5 Hz*, C*H*H 2’’’), 3.91 (1H, dd, *J = 7.0, 8.5 Hz*, CH*H* 2’’’), 3.56 (1H, br s, H5’’), 3.48 (2H, q, *J = 7.5 Hz*, C*H*2CH3), 3.27 – 3.23 (2H, m, H4’’ + H3’’), 3.05 (1H, br s, NH1’’), 1.49 (3H, s, 2’’-C*H*3), 1.30 (3H, s, C(C*H*3)2), 1.29 (3H, s, C(C*H*3)2), 1.09 (3H, t, *J = 7.5 Hz*, CH2C*H*3); 13CNMR (CDCl3, 100 MHz)  175.6, 175.3, 171.5, 135.3, 128.7, 128.5, 128.4, 109.8, 73.5, 68.1, 67.8, 67.5, 61.2, 57.3, 47.9, 34.0, 26.1, 24.9, 24.1; m/z(ESI, +ve ion) 455.1 (MK)+, 439.2 (MNa)+, 417.3 (MH)+, 359.2 (MH – OC(CH3)2)+; *vmax* 3323, 2984, 2938, 1774, 1737, 1694 cm-1; HRMS (LSIMS, +ve ion) Calculated for C22H28N2O6 (M+H)+ 417.2026. Found 417.2041.

Data for **3a**:15 white solid (0.16 g, 0.45 mmol, 100%). 1H NMR (CD3OD, 400 MHz)  7.46 – 7.14 (10H, m, Ar-H), 4.97 (1H, d, *J = 9.5 Hz*, H5’’), 3.84 (1H, dd, *J = 7.5, 9.5 Hz*, H4’’), 3.56 (1H, d, *J = 7.5* *Hz*, H3’’), 1.68 (3H, s, Me); 13C NMR(CD3OD, 100 MHz), 175.2, 172.1, 172.0, 138.1, 133.5, 129.9, 129.6, 129.5, 129.2, 128.5, 127.9, 66.9, 63.6, 57.2, 51.7, 23.8; m/z(ESI, +ve ion) 395.2 (MNa2)+, 389.0 (MK)+, 373.1 (MNa)+, 351.1 (MH)+, 305.2 (MH - CO2H)+; max2964, 1707 cm-1.

Data for **3b**: white solid (0.14 g, 0.38 mmol, 88%). 1H NMR (CD3OD, 400 MHz)  7.37 – 7.19 (7H, m, Ar-H), 6.98 - 6.90 (2H, m, Ar-H), 5.12 (1H, d, *J = 9.5 Hz*, H5’’), 3.87 (1H, dd, *J = 9.0, 9.5 Hz*, H4’’), 3.61 (3H, s, OC*H*3), 3.51 (1H, d, *J = 9.0 Hz*, H3’’), 1.68 (3H, s, 2’’-C*H*3); 13C NMR (CD3OD, 100 MHz)  (not all quarternary C’s seen) 159.3, 133.6, 132.4, 131.7, 129.8, 129.5, 127.7, 122.6, 112.5, 63.2, 56.4, 55.8, 49.7, 22.5; m/z(ESI, +ve ion) 442.2 (MKNa)+, 419.1 (MK)+, 403.1 (MNa)+, 381.2 (MH)+, 335.2 (MH - CO2H)+; *vmax* 2944, 1712, 1634, 1604 cm-1. HRMS(LSIMS, +ve ion) Calculated for C21H20N2O­5 (M+H)+ 381.1451. Found 381.1440.

Data for **3c**: white solid (0.33 g, 0.80 mmol, 100 %). 1H NMR (CD3OD, 300 MHz)  7.40 – 7.33 (3H, m, Ar-H), 7.27 – 7.25 (2H, m, Ar-H), 7.08 – 6.92 (3H, m, Ar-H), 5.16 (1H, d, *J = 9.5 Hz*, H5’’), 3.97 – 3.94 (4H, m, OC*H*3, H4’’), 3.84 (3H, s OC*H*3), 3.59 (1H, d, *J = 8.5 Hz*, H3’’), 1.74 (3H, s, 2’’-C*H*3); 13C NMR (CD3OD, 75 MHz)  (quarternary C’s not seen) 130.3, 128.5, 126.5, 125.4, 122.6, 115.8, 62.5, 62.2, 56.8, 56.8, 50.9, 23.2; m/z (ESI, +ve ion) 449.2 (MK)+, 433.1 (MNa)+, 411.2 (MH)+; *vmax* 2960, 1712 cm-1; HRMS (LSIMS, +ve ion) Calculated for C22H22N2O6 (M+H)+ 411.1556. Found 411.1558.

Data for **3d**: a white amorphous solid (0.15 g, 0.37 mmol, 98 %). 1H NMR (DMSO, 400 MHz)  7.51 – 7.47 (2H, m, Ar-H), 7.44 – 7.40 (1H, m, Ar-H), 7.14 – 7.12 (2H, m, Ar-H), 7.04 (1H, d, *J = 2.0 Hz*, Ar-H), 7.00 – 6.95 (2H, m, Ar-H), 4.79 – 4.77 (1H, d, *J = 9.0 Hz*, H5’’), 3.79 (3H, s, OC*H*3), 3.75 (1H, dd, *J = 7.5, 9.0 Hz*, H4’’), 3.73 (3H, s, OC*H*3), 3.50 (1H, d, *J = 7.5 Hz*, H3’’), 1.56 (3H, s, 2’’-C*H*3); 13C NMR(DMSO, 100 MHz)  176.2, 175.1, 174.4, 148.1, 139.2, 139.1, 132.3, 131.3, 128.7, 126.5, 124.9, 119.3, 111.6, 111.2, 66.3, 60.9, 55.4, 55.4, 55.0, 49.7, 23.3; m/z (ESI, +ve ion) 449.0 (MK)+, 433.1 (MNa)+, 411.2 (MH)+, 365.2 (MH – OMe – Me)+; *vmax* 3334, 2958, 1709 cm-1; HRMS (LSIMS, +ve ion) Calculated for C22H22N2O6 411.1556. Found 411.1563.

Data for **3e**: a white amorphous solid (0.28 g, 0.70 mmol, 84 %). 1H NMR (CD3CN/D2O, 400 MHz)  7.95 – 7.91 (3H, m, Ar-H), 7.69 – 7.67 (2H, m, Ar-H), 5.20 – 5.16 (1H, m, H1’’’), 4.71 (1H, dd, *J = 4.0, 9.5 Hz*, C*H*H 2’’’), 4.64 (1H, dd, *J = 5.0, 10.0 Hz*, H5’’), 4.43 – 4.36 (2H, m, CH*H* 2’’’ and H4’’), 4.09 (1H, d, *J = 9.0 Hz*, H3’’), 1.91 (3H, s, 2’’-C*H*3), 1.82 and 1.73 (2 x 3H, s, C(C*H*3)2); 13C NMR (CD3CN/D2O, 100 MHz)  174.4, 174.4, 174.0, 131.2, 129.3, 129.2, 126.5, 110.6, 71.4, 71.1, 66.1, 60.1, 53.9, 45.2, 25.2, 23.3, 21.3; m/z (ESI, +ve ion) 458.2 (MKNa2)+, 436.2 (MKNa)+, 413.1 (MK)+, 397.1 (MNa)+, 375.2 (MH)+, 317.1 (MH - OC(CH3)2)+; *vmax* 2958, 1716 cm-1; HRMS(LSIMS, +ve ion) Calculated for C19H22N2O6 (M+H)+ 375.1556. Found 375.1565.

Data for **3g**: white solid (0.11 g, 0.35 mmol, 95 %). 1H NMR(CD3OD, 400 MHz)  4.50 (1H, dt, *J = 6.0 Hz*, H1’’’), 4.17 (1H, dd, *J = 6.0, 9.0 Hz*, C*H*H 2’’’), 3.84 (1H, dd, *J = 6.0, 9.0 Hz*, CH*H* 2’’’), 3.79 (1H, dd, *J = 6.0, 9.5 Hz*, H5’’), 3.50 (1H, dd, *J = 8.5, 9.5 Hz*, H4’’), 3.35 (1H, q, *J = 7.0 Hz*, C*H*2­CH3), 3.28 (1H, d, *J = 8.5 Hz*, H3’’), 1.47 (3H, s, 2’’-C*H*3), 1.32 and 1.21 (2 x 3H, s, C(C*H*3)2), 1.00 (3H, t, *J = 7.0 Hz*, CH2C*H*3); 13CNMR (CD3CN, 100 MHz)  173.4, 172.9, 170.3, 108.6, 71.4, 68.4, 65.5, 59.2, 53.5, 44.7, 32.3, 24.0, 22.2, 20.5, 10.4; m/z (ESI, +ve ion) 371.1 (MNa2 – H)+, 349.2 (MNa)+, 327.2 (MH)+, 269.2 (MH – OC(CH3)2)+; *vmax* 2986, 1704, 1644, 1610 cm-1; HRMS (LSIMS, +ve ion) Calculated for C15H22N2O6 (M+H)+ 327.1556. Found 327.1571.

Data for **4a**: colourless oil(0.34 g, 0.82 mmol, 69 %). 1H NMR (CDCl3, 400 MHz)  7.43 – 7.23 (10H, m, Ar-H), 5.33 (1H, d, *J = 12.5 Hz*, C*H*HPh), 5.25 (1H, d, *J = 12.5 Hz*, CH*H*Ph), 4.83 (1H, d, *J = 9.5 Hz*, H5’’), 4.02 (1H, d, *J = 9.5 Hz*, H3’’), 3.88 (1H, dd, *J = 9.5, 9.5 Hz*, H4’’), 3.61 (3H, s, CO2C*H*3), 3.17 (3H, s, CO2C*H*3), 1.42 (3H, s, 2’’-C*H*3); 13C NMR (CDCl3, 100 MHz)  172.4, 171.9, 171.4, 139.2, 135.5, 128.6, 128.4, 128.3, 127.9, 127.7, 127.3, 67.6, 67.2, 62.9, 53.7, 52.5, 52.1, 51.5, 21.3; m/z(ESI, +ve ion) 434.1 (MNa)+, 412.2 (MH)+; max2950, 1728 cm-1; HRMS calculated for C23H25NO6 (M+H)+ 412.1760; Found 412.1777.

Data for **4b**: colourless oil (0.27 g, 0.61 mmol, 68%). 1H NMR (CDCl3, 400 MHz)  7.42 – 7.30 (5H, m, Ar-H), 7.26 – 7.20 (2H, m, Ar-H), 6.89 (1H, dd, *J = 7.5, 7.5 Hz*, Ar-H), 6.81 (1H, d *J = 8.0 Hz*, Ar-H), 5.29 (2H, d, *J = 12.5 Hz*, C*H*HPh), 5.23 (1H, d, *J = 12.5 Hz*, CH*H*Ph), 5.02 (1H, d, *J = 9.0 Hz*, H5’’), 3.98 (1H, d, *J = 9.0 Hz*, H3’’), 3.91 (1H, dd, *J = 9.0, 9.0 Hz*, H4’’), 3.75 (3H, s, OC*H*3), 3.62 (3H, s, CO2C*H*3), 3.13 (3H, s, CO2C*H*3), 1.41 (3H, s, 2’’-C*H*3); 13C NMR(CDCl3, 100 MHz)  173.5, 172.1, 171.4, 157.0, 135.6, 128.9, 128.6, 128.3, 128.2, 128.1, 126.7, 120.4, 110.2, 67.5, 67.4, 59.1, 55.2, 55.1, 52.0, 51.7, 51.3, 20.1; m/z(ESI, +ve ion)480.1 (MK)+, 464.1 (MNa)+, 442.2 (MH)+; *vmax* 2950 and 1728 cm-1; HRMS (LSIMS, +ve ion) Calculated for C24H27NO7 (M+H)+ 442.1866. Found 442.1862.

Data for **4c**: a white wax, mixture of diastereomers **a** and **b** in a 4.8:1 ratio(0.57 g, 1.20 mmol, 63%). 1H NMR (CDCl3, 400 MHz)  7.42 – 7.33 (5H + 5H, m, Ar-H (**a**) and (**b**)), 7.04 – 6.96 (1H + 2H, m, Ar-H (**a**) and (**b**)), 6.88 – 6.81 (2H + 1H, m, Ar-H (**a**) and (**b**)), 5.31 (1H, d, *J = 12.5 Hz*, C*H*HPh (**a**)), 5.23 (1H, d, *J = 12.5 Hz*, CH*H*Ph (**a**)), 5.18 (1H, br d, *J = 9.0 Hz*, H5’’ (**a**)), 5.17 (1H, d, *J = 12.0 Hz*, C*H*HPh (**b**)), 5.09 (1H, d, *J = 12.0 Hz*, CH*H*Ph (**b**)), 4.78 (1H, d, *J = 9.0 Hz*, H5’’ (**b**)), 4.00 (1H, d, *J = 8.0 Hz*, H3’’ (**a**)), 3.91- 3.87 (4H, m, H4’’ (**a**) and OC*H*3 (**a**)), 3.84 (3H + 3H, s, CO2C*H*3 (**a**) and OC*H*3 (**b**)), 3.77 ( 3H, s, OC*H*3 (**b**)), 3.63 (3H, s, CO2C*H*3 (**a**)), 3.60 (3H, s, CO2C*H*3 (**b**)), 3.58 – 3.45 (5H, m, CO2C*H*3 (**b**), H4’’ (**b**) and H3’’ (**b**)), 1.61 (3H, s, 2’’-C*H*3 (**b**)), 1.43 (3H, s, 2’’-C*H*3 (**a**)); 13C NMR (CDCl3, 400 MHz)  172.4, 172.0, 171.4, 152.3, 147.0, 135.6, 132.4, 128.6, 128.4, 128.3, 128.1, 124.2, 123.7, 119.4, 118.9, 112.0, 67.5, 67.4, 67.3, 60.8, 59.1, 58.5, 57.1, 55.8, 54.9, 54.7, 52.1, 52.0, 51.5, 26.1, 20.7; m/z (ESI, +ve ion) 510.1 (MK)+, 494.1 (MNa)+, 472.2 (MH)+; *vmax* 3339, 2949, 2836, 1728 cm-1; HRMS (LSIMS, +ve ion) Calculated for C25H29NO8 (M+H)+ 472.1971. Found 472.1968.

Data for **4d**: colourless gum, mixture of diastereoisomers **a** and **b** in a ratio of 6:1 (0.24 g, 0.51 mmol, 51%); 1H NMR(CDCl3, 400 MHz)  7.42 – 7.32 (5H + 5H, m, Ar-H (**a**) and (**b**)), 6.88 – 6.74 (3H + 3H, m, Ar-H (**a**) and (**b**)), 5.31 (1H, d, *J = 12.5 Hz*, C*H*HPh (**a**)), 5.25 (1H, d, *J = 12.5 Hz*, CH*H*Ph (**a**)), 5.17 (1H, d, *J = 12.5 Hz*, C*H*HPh (**b**)), 5.08 (1H, d, *J = 12.5 Hz*, CH*H*Ph (**b**)), 4.77 (1H, d, *J = 9.5 Hz*, H5’’ (**a**)), 4.38 (1H, d, *J = 9.0 Hz*, H5’’ (**b**)), 4.03 (1H, d, *J = 9.5 Hz*, H3’’ (**a**)), 3.88 – 3.83 (4H + 6H, m, H4’’ (**a**), OC*H*3 (**a**), 2 x OC*H*3 (**b**)), 3.79 (3H, s, OC*H*3 (**a**)), 3.61 (3H + 3H, s, CO2C*H*3 (**a**) and (**b**)), 3.49 – 3.43 (5H, m, CO2C*H*3 (**b**), H4’’ (**b**), H3’’ (**b**)), 3.25 (3H, s, CO2C*H*3 (**a**)), 2.77 (1H, br s, N*H* (**a**)), 1.71 (3H, s, 2’’-C*H*3 (**b**)), 1.41 (3H, s, 2’’-C*H*3 (**a**)); 13C NMR (CDCl3, 100 MHz)  Isomer **a**: 173.8, 171.4, 171.1, 148.7, 148.5 135.6, 132.6, 128.6, 128.4, 128.1, 119.5, 110.7, 110.5, 67.5, 67.0, 62.6, 55.8, 55.8, 53.5, 52.4, 52.1, 51.7, 51.7, 21.4; m/z (ESI, +ve ion) 510.1 (MK)+, 494.1 (MNa)+, 472.2 (MH)+; *vmax* 3361, 2995, 2950, 2836, 1728 cm-1; HRMS (LSIMS, +ve ion) Calculated for C25H29NO8 (M+H)+ 472.1971. Found 472.1976.

Data for **4e**: yellow oil containing diastereoisomers **a** and **b** (0.28 g, 0.65 mmol, 94%) in a 1.2:1 ratio. 1H NMR (CDCl3, 400 MHz)  7.43 – 7.29 (5H + 5H, m, Ar-H **a** and **b**), 5.26 (1H, d, *J = 13.0 Hz*, C*H*HPh **a**), 5.21 (1H, d, *J = 13.0 Hz*, CH*H*Ph **a**), 5.14 (1H, d, *J = 12.0 Hz*, C*H*HPh **b**), 5.06 (1H, d, *J = 12.0 Hz*, CH*H*Ph **b**), 4.29 – 4.24 (1H, m, H1’’’ **a**), 4.13 (1H, q, *J = 6.5 Hz*, H1’’’ **b**), 4.02 – 3.95 (2H, m, C*H*H 2’’’, **a** and **b**), 3.83 (1H, d, *J = 8.5 Hz*, H4’’ **a**), 3.78 – 3.74 (2H, m, CH*H* 2’’’ **a** and **b**), 3.72 (3H, s, CO2C*H*3 **b**), 3.67 (3H, s, CO2C*H*3 **a**), 3.65 – 3.64 (1H, m, H5’’ **b**), 3.62 (3H, s, CO2C*H*3), 3.61 – 3.57 (1H, m, H5’’ **a**), 3.49 (3H, s, CO2C*H*3 **b**), 3.41 (1H, dd, *J = 6.0, 8.5 Hz*, H4’’ **b**), 3.36 – 3.24 (2H, m, H3’’ **a** and **b**), 1.63 (3H, s, 2’’-C*H*3 **b**), 1.39 (3H, s, 2’’-C*H*3 **a**), 1.37, 1.36, 1.31, 1.28 (4 x 3H, s, C(C*H*3)2 **a** and **b**); 13C NMR (CDCl3, 100 MHz)  73.9, 171.6, 171.0, 135.5, 128.6, 128.5, 128.4, 128.2, 109.4, 74.4, 67.5, 67.5, 66.9, 66.9, 66.6, 62.7, 60.7, 57.7, 54.2, 52.4, 52.2, 52.1, 49.6, 49.2, 26.4, 26.7, 25.7, 25.2, 25.1, 21.4; m/z(ESI, +ve ion) 474.1 (MK+), 458.1 (MNa+), 436.2 (MH+), 378.1 (MH+ - OC(CH3)2); *vmax* 2987, 2952, 1729 cm-1. HRMS(LSIMS, +ve ion) Calculated for C22H29NO8 (M+H)+ 436.1971. Found 436.1980.

Data for **5a**: white solid (0.21 g, 0.65 mmol, 96 %). Decomposes at 198 – 200 ˚C. 1HNMR (CD3CN, 400 MHz)  7.32 – 7.16 (5H, m, Ar-H), 4.87 (1H, d, *J = 9.0 Hz*, H5’’), 3.72 (1H, d, *J = 7.5 Hz*, H3’’), 3.65 (1H, dd, *J = 7.5, 9.0 Hz*, H4’’), 3.61 (3H, s, CO2C*H*3), 3.08 (3H, s, CO2C*H*3), 1.30 (3H, s, 2’’-CH3); 13C NMR (CD3CN, 100 MHz)  (quarternary C’s not seen) 127.9, 127.5, 127.1, 62.3, 52.8, 51.9, 51.6, 50.9, 20.1; m/z(ESI, +ve ion) 344.1 (MNa)+, 322.1 (MH)+.

Data for **5b**: white solid (0.17 g, 0.48 mmol, 89 %). 1H NMR(CD3OD, 400 MHz)  7.50 – 7.45 (1H, m, Ar-Hc), 7.38 (1H, dd, *J = 2.0, 7.5 Hz*, Ar-Ha), 7.13 (1H, d, *J = 8.5 Hz*, Ar-Hd), 7.07 (1H, ddd, *J = 1.0, 7.5, 7.5 Hz*, Ar-Hb), 5.35 (1H, d, *J = 9.5 Hz*, H5’’), 4.15 – 4.09 (2H, m, H4’’ and H3’’), 3.94 (3H, s, OC*H*3), 3.81 (3H, s, CO2C*H*3), 3.27 (3H, s, CO2C*H*3), 1.59 (3H, s, 2’’-C*H*3); 13C NMR (CD3OD, 100 MHz)  (not all quarternary C’s were seen) 172.9, 171.8, 158.4, 132.5, 131.7, 122.2, 121.4, 112.4, 61.4, 56.0, 54.7, 53.0, 52.4, 51.2, 17.9; m/z(ESI, +ve ion) 390.0 (MK)+, 374.1 (MNa)+, 352.1 (MH)+, 320.1 (MH - OMe)+; *vmax*2951, 1732 cm-1.

Data for **5c**: a white amorphous solid containing diastereoisomers **a** and **b** in a 4.9:1 ratio (0.32 g, 0.85 mmol, 89 %). 1H NMR (CDCl3, 400 MHz)  7.04 – 6.96 (1H and 2H, m, Ar-H (**a**) and (**b**)), 6.91 – 6.85 (2H and 1H, m, Ar-H (**a**) and (**b**)), 6.30 (2H + 2H, br s, N*H*2 (**a**)and (**b**)), 5.43 (1H, d, *J = 9.5 Hz*, H5’’ (**a**)), 5.11 (1H, d, *J = 11.5 Hz*, H5’’ (**b**)), 3.94 (4H + 3H, br s, OC*H*3 (**a**) and (**b**), H4’’ (**a**)), 3.88 (1H, d, *J = 9.0 Hz*, H3’’ (**a**)), 3.84 (3H + 3H, s, OC*H*3 (**a**) and (**b**)), 3.73 (3H, s, CO2C*H*3 (**a**)), 3.62 (3H, s, CO2C*H*3 (**b**)), 3.55 (3H, s, CO2C*H*3 **b**), 3.53 – 3.42 (2H, m, H4’’ (**b**), H3’’ (**b**)), 3.22 (3H, s, CO2C*H*3 (**a**)), 1.76 (3H, s, 2’’-C*H*3 (**b**)), 1.48 (3H, s, 2’’-C*H*3 (**a**)); 13C NMR (CDCl3, 100 MHz)  (quarternary C’s for **b** not seen)174.3, 170.9, 170.8, 152.2, 146.9, 128.9, 124.5, 123.8, 120.1, 119.4, 113.4, 113.0, 68.3, 60.9, 57.4, 56.6, 55.9, 53.3, 52.4, 51.8, 51.3, 51.0, 18.4; m/z (ESI, +ve ion) 426.1 (MNa2 – H)+, 404.1 (MNa)+, 482.1 (MH)+, 350.1 (M – OMe)+; *vmax* 2951, 2840, 1731 cm-1; HRMS (LSIMS, +ve ion) Calculated for C18H23NO8 (M+H) 382.1501. Found 382.1497.

Data for **5d**: off-white solid containing diastereoisomers **a** and **b** in a 6:1 ratio (0.15 g, 0.39 mmol, 93 %). 1H NMR (DMSO/CD3OD, 400 MHz)  7.00 – 6.81 (3H + 3H, m, Ar-H (**a**) and (**b**)), 4.80 (1H, d, *J = 9.5 Hz*, H5’’ (**a**)), 4.57 (1H, d *J = 11.0 Hz*, H5’’ (**b**)), 3.99 (1H, d, *J = 10.5 Hz*, H3’’(**a**)), 3.82 (1H, dd, *J = 9.5, 10.5 Hz*, H4’’ (**a**)), 3.78 – 3.76 (6H + 6H, s, OC*H*3 (**a**), OC*H*3 (**a**), OC*H*3 (**b**), OC*H*3 (**b**)), 3.68 (3H, s, CO2C*H*3 (**b**)), 3.66 (3H, s, CO2C*H*3 (**a**)), 3.56 (3H, s, CO2C*H*3 (**b**)), 3.48 – 3.37 (2H, m, H4’’ (**b**), H3’’ (**b**)) 3.27 (3H, s, CO2C*H*3 (**a**)), 1.38 (3H, s, 2’’-C*H*3 (**b**)), 1.31 (3H, s, 2’’-C*H*3 (**a**)); 13C NMR (DMSO/CD3OD, 100 MHz)  signals for isomer **a**) 177.1, 172.8, 172.7, 150.3, 150.2, 134.6, 121.2, 113.2, 112.8, 68.3, 63.1, 56.9, 56.9, 54.1, 52.8, 52.2, 52.2, 22.1; m/z (ESI, +ve ion) 426.1 (MNa2 – H)+, 420.1 (MK)+, 404.1 (MNa)+, 382.1 (MH)+, 350.2 (M – OMe)+; *vmax* 2955, 1734, 1616, 1147, 1021 cm-1; HRMS (LSIMS, +ve ion) Calculated for C18H23NO8 382.1502. Found. 382.1499.

Data for **5e**: a white amorphous solid containing a mixture of diastereomers **a** and **b** in a 1.2:1 molar ratio (0.24 g, 0.64 mmol, 100 %). 1H NMR(CD3OD, 400 MHz)  4.61 – 4.55 (1H + 1H, m, H1’’’ (**a**) and (**b**)), 4.21 – 4.14 (2H + 2H, m, H5’’ (**a**) and (**b**) + C*H*H 2’’’ (**a**) and (**b**)), 3.92 – 3.84 (2H + 2H, m, CH*H* 2’’’ (**a**) and (**b**), H4’’ (**a**) and (**b**)), 3.78 - 3.71 (3H + 3H, m, 2 x CO2C*H*3 (**a**) and (**b**)), 3.46 – 3.35 (1H + 1H, m, H3’’ (**a**) and (**b**)), 1.75, 1.54, 1.51, 1.47, 1.38, 1.33 (9H + 9H, 6 x s, 2’’-C*H*3 (**a**) and (**b**), C(C*H*3)2 (**a**) and (**b**)); 13C NMR (CD3OD, 100 MHz)  172.1, 171.4, 171.2, 170.9, 111.6, 76.5, 74.8, 74.2, 71.7, 70.8, 67.7, 67.5, 67.3, 62.2, 56.9, 54.2, 53.4, 53.1, 52.9, 49.7, 48.3, 26.8, 24.9, 24.8, 23.3, 19.4; m/z(ESI, +ve ion) 368.1 (MNa)+, 346.1 (MH)+, 288.1 (MH - OC(CH3)2)+, 256.1 (M - CO2CH3 – 2 x Me)+; vmax 2985, 2953, 1734, 1637 cm-1; HRMS (LSIMS, +ve ion) Calculated for C15H23NO8 (M+H)+ 346.1505. Found 346.1491.

Data for **6a**: colourless oil (0.26 g, 0.63 mmol, 73%). 1H NMR (CDCl3, 400 MHz)  7.38 – 7.25 (10H, m, Ar-H), 5.26 (1H, d, J = 12.0 Hz, CHHPh), 5.15 (1H, d, J = 12.0 Hz, CHHPh), 4.63 (1H, d, J = 6.5 Hz, H5’’), 3.46 – 3.42 (4H, m, H4’’ and CO2CH3), 3.28 (1H, d, J = 7.0 Hz, H3’’), 3.25 (3H, s, CO2CH3), 1.71 (3H, s, 2’’-CH3); 13C NMR (CDCl3, 100 MHz)  174.1, 171.1, 170.7, 137.4, 135.4, 128.8, 128.5, 128.4, 128.3, 127.8, 126.8, 68.4, 67.6, 63.7, 57.7, 53.1, 51.8, 51.3, 28.6; m/z(ESI, +ve ion) 450.1 (MK)+, 434.1 (MNa)+, 412.2 (MH)+; max3032, 2949, 1729 cm-1; HRMS calculated for C23H25NO6 (M+H)+ 412.1760; Found 412.1768.

Data for **6b**: colourless oil (0.39 g, 0.88 mmol, 95 %). 1H NMR (CDCl3, 400 MHz)  7.34 – 7.29 (6H, m, Ar-H), 7.22 (1H, dd, *J = 8.5 Hz*, Ar-H), 6.90 (1H, dd, *J = 7.5, 7.5 Hz*, Ar-H), 6.83 (1H, d, *J = 8.0 Hz*, Ar-H), 5.29 (1H, d, *J = 11.0 Hz*, CH*H*Ph), 5.13 (1H, d, *J = 11.0 Hz*, C*H*HPh), 4.85 (1H, d, *J = 6.0 Hz*, H5’’), 3.82 (3H, s, OC*H*3), 3.72 (1H, br s, NH1’’), 3.62 (1H, dd, *J = 6.0, 7.5 Hz*, H4’’), 3.43 (3H, s, CO2C*H*3), 3.30 (1H, d, *J = 7.5 Hz*, H3’’), 3.21 (3H, s, CO2C*H*3), 1.71 (3H, s, 2’’-C*H*3); 13C NMR (CDCl3, 100 MHz)  174.3, 171.3, 170.7, 156.8, 135.6, 128.8, 128.6, 128.5, 128.3, 126.0, 125.5, 120.4, 110.0, 67.5, 67.4, 58.4, 57.5, 55.4, 51.7, 51.3, 51.0, 28.9; m/z(ESI, +ve ion)480.1 (MK)+, 464.1 (MNa)+, 442.2 (MH)+; *vmax*2950, 1730 cm-1; HRMS(LSIMS, +ve ion) Calculated for C24H27NO7 (M+H)+ 442.1866. Found 442.1878.

Data for **6c**: colourless oil(0.46 g, 0.97 mmol, 50%); 1H NMR (CDCl3, 400 MHz)  7.41 – 7.32 (5H, m, Ar-H), 7.02 – 6.96 (2H, m, Ar-H), 6.83 (1H, dd, *J = 3.0, 6.5 Hz*, Ar-H), 5.28 (1H, d, *J = 12.0 Hz*, C*H*HPh), 5.14 (1H, d, *J = 12.0 Hz*, CH*H*Ph), 4.91 (1H, d, *J = 6.5 Hz*, H5’’), 3.87 (3H, s, OC*H*3), 3.85 (3H, s, OC*H*3), 3.50 (1H, dd, *J = 6.5, 6.5 Hz*, H4’’), 3.42 (3H, s, CO2C*H*3), 3.32 (1H, d, *J = 6.5 Hz*, H3’’), 3.24 (3H, s, CO2C*H*3), 1.71 (3H, s, 2’’-C*H*3); 13C NMR (CDCl3, 100 MHz)  174.4, 171.2, 170.6, 152.1, 146.8, 135.5, 130.8, 128.8, 128.5, 128.4, 123.7, 118.0, 111.9, 67.6, 67.6, 60.7, 58.1, 57.5, 55.7, 52.1, 51.7, 51.1, 28.9; m/z (ESI, +ve ion) 510.1 (MK+), 494.1 (MNa)+, 472.2 (MH)+, 336.2 (M - CO2CH2Ph)+; *vmax* 3367, 2948, 2836, 1729 cm-1; HRMS (LSIMS, +ve ion) Calculated for C25H29NO8 (M+H)+ 472.1971. Found 472.1987.

Data for **6d**: a white amorphous solid (0.23 g, 0.48 mmol, 48%); 1H NMR (CDCl3, 400 MHz)  7.39 – 7.32 (5H, m, Ar-H), 6.92 – 6.87 (2H, m, Ar-H), 6.78 (1H, d, *J = 8.5 Hz*, Ar-H), 5.24 (1H, d, *J = 12.0 Hz*, C*H*HPh), 5.14 (1H, d, *J = 12.0 Hz*, CH*H*Ph), 4.57 (1H, br d, *J = 6.5 Hz*, H5’’), 3.85 (3H, s, OC*H*3), 3.84 (3H, s, OC*H*3), 3.61 (1H, br s, NH1’’), 3.49 – 3.42 (4H, m, CO­2C*H*3 and H4’’), 3.31 (3H, s, CO2C*H*3), 3.26 (1H, d, *J = 7.0 Hz*, H3’’), 1.68 (3H, s, 2’’-C*H*3); 13C NMR(CDCl3, 100 MHz)  171.2, 170.9, 148.7, 148.5, 140.6, 135.4, 130.2, 128.7, 128.5, 128.4, 119.3, 110.8, 110.2, 68.4, 67.6, 63.5, 57.5, 55.8, 55.8, 53.0, 51.8, 51.4, 28.5; m/z (ESI, +ve ion) 510.1 (MK)+, 494.1 (MNa)+, 472.2 (MH)+; *vmax*3336, 3013, 2963, 2899, 1722 cm-1; HRMS (LSIMS, +ve ion) Calculated for C25H29NO8 (M+H)+ 472.1971. Found 472.1981.

Data for **6e**: pale yellow solid (0.13 g, 0.30 mmol, 44%). 1H NMR (CDCl3, 400 MHz)  7.38 – 7.29 (5H, m, Ar-H), 5.17 (1H, d, *J = 12.0 Hz*, C*H*HPh), 5.10 (1H, d, *J = 12.0 Hz*, CH*H*Ph), 4.20 (1H, dt, *J = 6.5, 6.5 Hz*, H1’’’), 4.14 – 4.05 (1H, m, C*H*H 2’’’), 3.98 (1H, dd, *J = 6.0, 8.5 Hz*, CH*H* 2’’’), 3.86 (3H, s, CO2C*H*3), 3.41 (3H, s, CO2C*H*3), 3.32 – 3.30 (2H, m, H5’’ and H4’’), 3.12 (1H, d, *J = 7.0 Hz*, H3’’), 2.58 (1H, br s, NH1’’), 1.54 (3H, s, 2’’-C*H*3), 1.38 (3H, s, C(C*H*3)2), 1.29 (3H, s, C(C*H*3)2); 13C NMR (CDCl3, 100 MHz)  173.6, 171.6, 170.8, 135.3, 128.8, 128.7, 128.5, 128.4, 109.4, 109.3, 76.3, 75.0, 71.3, 68.8, 68.4, 67.6, 66.8, 57.9, 51.8, 51.7, 49.2, 28.1, 26.9, 26.7, 25.4, 25.2; m/z(ESI, +ve ion) 474.1 (MK+), 458.1 (MNa+), 436.2 (MH+), 378.2 (MH+ - OC(CH3)2); *vmax* 2950, 2985, 1733 cm-1; HRMS(LSIMS, +ve ion) Calculated for C22H29NO8 (M+H)+ 436.1971. Found 436.1969.

Data for **7a**: white solid (0.18 g, 0.57 mmol, 94%). Decomposes at 200 – 202 ˚C. 1HNMR (CD3OD, 400 MHz)  7.44 – 7.39 (5H, m, Ar-H), 5.10 (1H, d, *J = 7.5 Hz*, H5’’), 3.83 (1H, dd, *J = 7.5, 7.5 Hz*, H4’’), 3.74 (3H, s, CO2C*H*3), 3.55 (1H, d, *J = 7.5 Hz*, H3’’), 3.32 (3H, s, CO2C*H*3), 1.81 (3H, s, 2’’-C*H*3); 13C NMR (CD3OD, 100 MHz)  (not all quarternary C’s seen) 171.6, 134.1, 130.2, 130.0, 128.0, 72.4, 62.7, 56.0, 52.9, 52.0, 51.9, 25.1; m/z(ESI, +ve ion) 344.1 (MNa)+, 322.1 (MH)+, 276.2 (MH - CO2H)+; max 3006, 2951, 1743, 1724, 1655 cm-1; HRMS calculated for C16H19NO6 (M+H)+ 322.1291; Found 322.1280.

Data for **7b**: white solid(0.28 g, 0.79 mmol, 100 %). 1H NMR(CD3OD, 400 MHz)  7.32 – 7.23 (2H, m, Ar-H), 6.98 – 6.91 (2H, m, Ar-H), 5.12 (1H, d, *J = 7.0 Hz*, H5’’), 3.83 (3H, s, OC*H*3), 3.69 (1H, dd, *J = 7.0, 7.0 Hz*, H4’’), 3.62 (3H, s, CO2C*H*3), 3.49 (1H, d, *J = 7.0 Hz*, H3’’), 3.35 (3H, s, CO2C*H*3) 1.73 (3H, s, 2’’-C*H*3); 13C NMR (CDCl3, 100 MHz)  173.1, 172.5, 161.4, 132.6, 128.9, 122.9, 121.2, 113.5, 72.5, 59.7, 57.3, 57.1, 53.9, 52.9, 51.9, 26.4; m/z(ESI, +ve ion) 390.0 (MK)+, 374.1 (MNa)+, 352.1 (MH)+, 320.1 (MH - OMe)+, 306.1 (MH - OMe – Me)+; *vmax* 2951, 1737 cm-1; HRMS(LSIMS, +ve ion) Calculated for C17H21NO7 (M+H)+ 352.1396. Found 352.1384.

Data for **7c**: a white amorphous solid (0.26 g, 0.69 mmol, 78 %). 1H NMR (DMSO, 400 MHz)  7.15 – 6.99 (3H, m, Ar-H), 4.89 (1H, d, *J = 6.0 Hz*, H5’’), 3.86 (3H, s, OC*H*3), 3.83 (3H, s, OC*H*3), 3.63 (3H, s, CO2C*H*3), 3.56 (1H, d, *J = 7.0 Hz*, H3’’), 3.50 (1H, dd, *J = 6.0, 7.0 Hz*, H4’’), 3.19 (3H, s, CO2C*H*3), 1.61 (3H, s, 2’’-C*H*3); m/z (ESI, +ve ion) 404.1 (MNa)+, 382.1 (MH)+; *vmax* 2946, 2838, 1736 cm-1; HRMS (LSIMS, +ve ion) Calculated for C18H23NO8 382.1502. Found 382.1497.

Data for **7d**: off-white solid (0.16 g, 0.42 mmol, 100 %). 1H NMR (CD3OD, 400 MHz)  7.09 (1H, s, Ar-Ha), 6.98 (2H, s, Ar-Hb+c), 5.07 (1H, d, *J = 8.0 Hz*, H5’’), 3.88 (1H, dd, *J = 8.0, 8.0 Hz*, H4’’), 3.84 (3H, s, OC*H*3), 3.83 (3H, s, OC*H*3), 3.75 (3H, s, CO2C*H*3), 3.52 (1H, d, *J = 8.0 Hz*, H3’’), 3.36 (3H, s, CO2C*H*3), 1.79 (3H, s, 2’’-CH3); 13C NMR (CD3OD, 100 MHz)  175.1, 172.6, 171.6, 151.3, 150.8, 126.2, 121.4, 112.8, 112.1, 72.8, 62.5, 56.6, 56.5, 55.5, 52.9, 52.3, 51.6, 24.5; m/z (ESI, +ve ion) 404.1 (MNa)+, 382.1 (MH)+; *vmax* 2952, 1738, 1650 cm-1; HRMS (LSIMS, +ve ion) Calculated for C18H23NO8 382.1502. Found 382.1496.

Data for **7e**: a white solid (0.14 g, 0.41 mmol, 90 %). 1H NMR(CD3CN, 400 MHz)  7.51 (2H, br s, N*H*2), 4.41 – 4.25 (1.5H, m, H1’’’, CH2 2’’’), 4.07 – 4.00 (1.8H, m, CH2 2’’’, H5’’), 3.91 (0.7H, m, H5’’), 3.71 – 3.62 (6H, m, CO2C*H*3 x 2), 3.57 – 3.48 (1H, m, H4’’), 3.41 – 3.36 (1H, m, H3’’), 1.80, 1.77 (3H, s, 2’’-C*H*3), 1.46, 1.38, 1.31, 1.26 (1.4H, 1.6H, 1.4H and 1.6H, s, C(C*H*3)2); 13C NMR (CD3CN, 100 MHz)  172.7, 172.6, 170.9, 169.6, 169.5, 169.5, 110.0, 109.7, 73.1, 72.3, 70.9, 69.9, 67.0, 66.6, 60.2, 59.9, 54.3, 54.1, 52.0, 51.9, 51.7, 51.6, 46.4, 46.0, 26.1, 25.8, 25.7, 24.6, 24.3, 24.0, 23.9, 23.6; m/z(ESI, +ve ion) 368.1 (MNa)+, 346.2 (MH)+, 288.2 (MH – OC(CH3)2)+; *vmax* 2984, 2950, 1739, 1634, 1205 cm-1; HRMS(LSIMS, +ve ion) Calculated for C15H23NO8­ (M+H)+ 346.1502. Found 346.1510.

*Data for*  **8a**. Melting point 149 – 153 ˚C. 1H NMR (CD3OD, 400 MHz)  7.29 (2H, d, *J = 7.5 Hz*, Ar-H), 7.18 – 7.15 (2H, m, Ar-H), 7.09 - 7.07 (1H, m, Ar-H), 7.05 – 7.00 (2H, m, Ar-H), 6.91 – 6.87 (3H, m, Ar-H), 4.87 (1H, d, *J = 6.0 Hz*, H5’’), 4.14 (1H, d, *J = 17.5 Hz*, NHC*H*H of glycine linker), 4.08 (1H, d, *J = 17.5* *Hz*, NHCH*H* of glycine linker), 3.64 (1H, dd, *J = 6.0, 8.5 Hz*, H4’’), 3.52 (1H, d, *J = 8.5 Hz*, H3’’), 1.59 (C*H*3); 13C NMR (CD3OD, 100 MHz)  179.1, 176.6, 170.7, 170.7, 138.5, 137.5, 129.5, 129.4, 129.1, 127.9, 125.7, 122.2, 68.8, 68.7, 56.8, 55.2, 40.7, 23.1; m/z(ESI, +ve ion) 430.1 (MNa)+, 408.1 (MH)+, 392.2 (MH - Me)+; *vmax* 3301, 1701, 1697 cm-1; HRMS(LSIMS, +ve ion)calculated for C22H21N3O5 (M+H)+ 408.1559; Found 408.1564.

*Data for*  **8b**. 1H NMR (CD3OD, 400 MHz)  7.68 – 7.66 (0.6H, d, *J = 7.5 Hz*, Ar-H), 7.36 – 6.81 (8.4H, m, Ar-H), 5.15 – 5.13 (1H, m, H5’’), 4.20 (0.4H, d, *J = 17.0 Hz*, NHC*H*2 of glycine linker), 4.14 (0.4H, d, *J = 17.0 Hz*, NHC*H*2 of glycine linker), 4.03 – 3.95 (1H, m, NHC*H*2 of glycine linker, H4’’), 3.91 - 3.83 (4.2H, m, OC*H*3, NHC*H*2 of glycine linker, H4’’), 3.66 (0.4H, d, *J = 9.0 Hz*, H3’’), 3.43 (0.6H, d, *J = 8.0 Hz*, H3’’), 1.69 (1.2H, s, 2’’-C*H*3), 1.65 (1.8H, s, 2’’-C*H*­3­); 13C NMR (CD3OD, 100 MHz)  176.9, 176.0, 159.0, 158.0, 138.3, 133.5, 130.4, 129.9, 129.6, 129.6, 128.0, 127.6, 125.9, 122.5, 121.5, 121.4, 111.3, 68.4, 63.9, 57.9, 56.6, 56.3, 56.2, 56.1, 52.7, 48.5, 42.5, 23.0, 22.8; m/z (ESI, +ve ion) 482.1 (MNa2)+, 460.1 (MNa)+, 438.2 (MH)+, 395.2 (MH - CO2H)+; *vmax*3374, 3303, 2944, 1701, 1685, 1654 cm-1; HRMS(LSIMS, +ve ion) Calculated for C23H23N3O6 (M+H)+ 438.1665. Found 438.1680.

Data for **8c**: off-white solid 120 mg (HATU coupling 82%, deprotection 100 %). 1H NMR (CD3OD, 400 MHz)  7.26 – 7.19 (4H, m, Ar-H), 6.98 – 6.90 (3H, m, Ar-H), 6.83 – 6.82 (1H, m, Ar-H), 5.08 (1H, d, *J = 9.0 Hz*, H5’’), 3.93 (1H, d, *J = 18.0 Hz*, NHC*H*H of glycine linker), 3.86 (3H, s, OC*H*3), 3.77 (1H, d, *J = 18.0 Hz*, NHCH*H* of glycine linker), 3.73 (3H, s, OC*H*3), 3.68 (1H, dd, *J = 8.0, 9.0 Hz*, H4’’), 3.31 (1H, d, *J = 8.0 Hz*, H3’’), 1.53 (3H, s, 2’’-*C*H3); 13C NMR (CD3OD, 100 MHz)  176.2, 175.9, 175.5, 173.6, 153.1, 148.6, 139.2, 133.5, 129.9, 128.0, 126.2, 124.9, 120.0, 113.6, 68.2, 61.2, 57.4, 56.3, 56.3, 49.1, 42.2, 22.9; m/z (ESI, +ve ion) 506.0 (MK)+, 490.1 (MNa)+, 468.2 (MH)+, 365.1 (M – CONHCH2CO2H)+; *vmax* 3620, 3367, 2959, 2871, 1710, 1663 cm-1; HRMS (LSIMS, +ve ion) Calculated for C24H25N3O7 (M+H)+ 468.1771. Found 468.1787.

Data for **8e**: white solid 0.15 g (HATU coupling 76%, deprotection 97 %). 1H NMR (CD3OD, 400 MHz)  7.43 (2H, d, *J = 8.0 Hz*, Ar-Hc), 7.30 (2H, dd, *J = 8.0, 8.0 Hz*, Ar-Hb), 7.10 (1H, d, *J = 8.0 Hz*, Ar-Ha), 4.35 – 3.34 (8H, m, CH2 2’’’, H1’’’, H5’’, H4’’, H3’’, NHC*H*2 of glycine linker), 1.63, 1.57 (2.5H + 0.5H, s, 2’’-C*H*3), 1.44, 1.38, 1.32 and 1.19 (0.5H, 2.5H, 0.5H and 2.5H, s, C(C*H*3)2); 13C NMR (CD3OD, 100 MHz)  178.7, 176.0, 176.0, 170.5, 138.9, 130.1, 129.9, 129.8, 128.2, 125.9, 121.9, 121.9, 111.1, 110.7 78.6, 76.0, 69.1, 68.4, 68.2, 67.7, 56.3, 51.1, 47.2, 41.1, 27.1, 26.9, 25.5, 23.2, 23.2; m/z(ESI, +ve ion)470.1 (MK)+, 454.2 (MNa)+, 432.2 (MH)+, 374.2 (MH - OC(CH3)2)+; *vmax* 2923, 1708 cm-1; HRMS(LSIMS, +ve ion) Calculated for C21H25N3O7 (M+H)+ 432.1770. Found 432.1780.

Data for **9a**: white solid, 56 mg (EDCI coupling 57%, deprotection 71%). Melting point 144 – 146 ˚C. 1H NMR (CD3OD, 400 MHz)  7.30 (2H, d, *J = 8.5 Hz*, Ar-H), 7.20 - 7.13 (3H, m, Ar-H), 4.87 (peak hidden behind D2O, H5’’), 3.88 (2H, s, NHC*H*2 of glycine linker), 3.73 (1H, dd, *J = 8.5, 8.5 Hz*, H4’’), 3.62 – 3.61 (4H, m, H3’’ and CO2C*H*3), 3.04 (3H, s, CO2C*H*3), 1.30 (3H, s, 2’’-C*H*3); 13C NMR (CD3OD, 100 MHz)  177.2, 173.2, 173.2, 173.0, 141.9, 129.1, 128.8, 128.7, 67.8, 63.4, 54.3, 53.8, 52.7, 52.0, 42.2, 21.3; m/z(ESI, +ve ion) 417.1 (MK)+, 401.1 (MNa)+, 379.1 (MH)+; *vmax*3345, 3302, 1719, 1658 cm-1; HRMS calculated for C18H22N2O7 (M+H)+ 379.1505; Found 379.1516.

Data for **9b**: white solid, 0.13 g (HATU coupling 89%, deprotection 100 %). 1H NMR (CD3OD, 400 MHz)  7.48 (1H, dd, *J = 1.5, 7.5 Hz*, Ar-Ha), 7.26 (1H, dt, *J = 1.5, 8.5 Hz*, Ar-Hb), 6.93 (2H, m, Ar-Hc&d), 5.21 (1H, d, *J = 9.0 Hz*, H5’’), 4.04 (1H, d, *J = 11.5 Hz*, NHC*H*H of glycine linker), 4.00 (1H, d, *J = 11.5 Hz*, NHCH*H* of glycine linker), 3.91 (1H, dd, *J = 9.0, 9.0 Hz*, H4’’), 3.84 (3H, s, OC*H*3), 3.74 (3H, s, CO2C*H*3), 3.68 (1H, d, *J = 9.0 Hz*, H3’’), 3.11 (3H, s, CO2C*H*3), 1.45 (3H, s, 2’’-C*H*3); 13C NMR(CD3OD, 100 MHz)  176.8, 173.4, 173.4, 173.1, 158.6, 130.3, 129.3, 127.8, 121.5, 111.5, 68.0, 58.9, 55.9, 55.3, 52.9, 52.1, 49.9, 42.7, 20.3; m/z(ESI, +ve ion) 431.1 (MNa)+, 409.2 (MH)+; *vmax*3370, 2952, 1718, 1700 cm-1; HRMS(LSIMS, +ve ion) Calculated for C19H24N2O8 (M+H)+ 409.1611. Found 409.1625.

Data for **9c**: white amorphous solid 91 mg (HATU coupling 67%, deprotection 100%). 1H NMR (CD3OD, 400 MHz)  6.98 – 6.91 (2H, m, Ar-H), 6.88 – 6.71 (1H, m, Ar-H), 5.21 (1H, d, *J = 8.5 Hz*, H5’’), 4.03 (1H, d, *J = 18.0 Hz*, NHC*H*H of glycine linker), 3.88 (1H, d, *J = 18.0 Hz*, NHCH*H* of glycine linker), 3.84 (3H, s, OC*H*3), 3.78 (3H, s, OC*H*3), 3.72 - 3.62 (5H, m, CO2C*H*3, H4’’, H3’’), 3.09 (3H, s, CO2C*H*3), 1.38 (3H, s, 2’’-C*H*3); 13C NMR(CD3OD, 100 MHz)  175.2, 171.3, 170.8, 151.4, 146.0, 131.8, 124.5, 122.9, 118.1, 111.2, 65.5, 59.8, 55.6, 54.8, 52.9, 51.3, 50.6, 50.6, 40.4, 20.0; m/z (ESI, +ve ion) 461.1 (MNa)+, 439.1 (MH)+; *vmax* 2951, 2840, 1731 cm-1; HRMS (LSIMS, +ve ion) Calculated for C20H26N2O9 (M+H)+ 439.1722. Found 439.1717.

Data for **9d**: white solid 12 mg (HATU coupling 34%, deprotection 65 %). **1H NMR** (CD3OD/CD3CN, 400 MHz)  7.32 – 7.28 (3H, m, Ar-H), 7.06 – 6.99 (4H, m, Ar-H), 6.81 (1H, d, *J = 8.5 Hz*, Ar-H), 4.80 (1H, d, *J = 9.0 Hz*, H5’’), 3.92 (1H, d, *J = 18.0 Hz*, NHC*H*H of glycine linker), 3.77 (1H, d, *J = 18.0 Hz*, NHCH*H* of glycine linker), 3.71 (3H, s, OC*H*3), 3.69 (3H, s, OC*H*3), 3.59 (1H, dd, *J = 8.5, 9.0 Hz*, H4’’), 3.27 (1H, d, *J = 8.5 Hz*, H3’’), 1.52 (3H, s, 2’’-C*H*3); 13C NMR (CD3OD/CD3CN, 100 MHz)  (quarternary C’s not seen) 129.1, 128.7, 125.4, 120.3, 117.5, 111.5, 66.9, 61.4, 55.6, 55.5, 55.2, 49.2, 41.1, 22.0; m/z (ESI, +ve ion) 506.1 (MK)+, 490.1 (MNa)+, 468.2 (MH)+; *vmax* 3641, 2957, 1708, 1632, 1023 cm-1; HRMS (LSIMS, +ve ion) Calculated for C24H25N3O7 468.1771. Found 468.1762.

Data for **9e**: white solid 0.14 g (HATU coupling 88%, deprotection 97%). 1H NMR (CD3CN, 400 MHz)  8.14 (1H, br s, N*H*CH2 of glycine linker), 4.18 – 2.96 (14H, m, CH2 2’’’, H1’’’, H5’’, H4’’, H3’’, NHC*H*2 of glycine linker, CO2C*H*3, CO2C*H*3), 1.55, 1.52, 1.46, 1.37, 1.35, 1.33, 1.31, 1.29, 1.26, 1.25, 1.24 (9H in total, m, C(C*H*3)2, 2’’-C*H*3); m/z (ESI, +ve ion) 425.1 (MNa)+, 403.2 (MH)+, 345.2 (MH - OC(CH3)2)+; *vmax*2984, 1718 cm-1; HRMS(LSIMS, +ve ion) Calculated for C17H26N2O9 (M+H)+ 403.1716. Found 403.1724.

Data for **10a/11a**: white solid, 0.16 g (EDCI coupling 32%, deprotection 97%), containing **10a** and bicyclic **11a** in a 1:1 molar ratio. 1H NMR (CD3OD, 400 MHz)  .48 (2H, d, *J = 7.5 Hz*, Ar-H) 7.22 (3H + 5H, m, Ar-H), 5.01 (1H, d, *J = 6.5 Hz*, H5’’ **11a**), 4.95 (1H, d, *J = 7.0 Hz*, H5’’ **10a**), 4.26 (1H, d, *J = 17.5 Hz*, NHC*H*H of glycine linker **11a**), 4.18 (1H, d, *J = 17.5 Hz*, NHCH*H* of glycine linker **11a**), 4.02 (1H, d, *J = 18.0 Hz*, NHC*H*H of glycine linker **10a**), 3.97 (1H, d, *J = 18.0 Hz*, NHCH*H* of glycine linker **10a**), 3.75 (1H, dd, *J = 6.5, 8.0 Hz*, H4’’ **11a**), 3.70 (3H, s, CO2C*H*3 **11a**), 3.56 (2H, m, H3’’ **11a** and H4’’ **10a**), 3.46 (1H, d, *J = 7.0 Hz*, H3’’ **10a**), 3.18 (3H, s, CO2C*H*3 **10a**), 3.13 (3H, s, CO2C*H*3 **11a**), 1.73 (3H, s, 2’’-C*H*3 **11a**), 1.65 (3H, s, 2’’-C*H*3 **11a**); 13C NMR (CD3OD, 100 MHz)  178.7, 176.4, 172.9, 172.6, 172.5, 170.5, 138.5, 137.4, 129.4 129.4, 128.5, 127.8, 68.0, 67.9, 63.3, 56.8, 55.7, 53.7, 53.2, 52.8, 52.2, 51.9, 50.0, 42.4, 40.7, 27.2, 22.42; m/z(ESI, +ve ion) 401.1 (M(**10a**)Na)+, 379.1 (M(**10a**)H)+, 347.1 (M(**11a**)H)+; *vmax*3328, 2989, 1746, 1711 cm-1; HRMS(LSIMS, +ve ion) Calculated for C18H22N2O7 (M**a**+H)+ 379.1497. Found 379.1505.

Data for **10b/11b**: white solid 84 mg (HATU coupling 99%, deprotection 93 %) containing **10b** and bicyclic **11b** in a 1:2 molar ratio. 1H NMR (CD3OD, 400 MHz)  7.71 – 7.69 (1H, m, Ar-H **10b**), 7.34 – 7.32 (1H, m, Ar-H **11b**), 7.27 – 7.21 (1H + 1H, m, Ar-H), 6.95 – 6.85 (2H + 2H, m, Ar-H), 5.11 – 5.07 (1H + 1H, m, H5’’), 4.25 (1H, d, *J = 17.0 Hz*, NHC*H*H **11b** of glycine linker), 4.16 (1H, d, *J = 17.0 Hz*, NHCH*H* **11b** of glycine linker), 4.04 – 3.83 (6H + 4H, m, NHC*H*2 **10b** of glycine linker, H4’’, OC*H*3), 3.68 (3H, s, CO2C*H*3 **10b**), 3.62 (1H, d, *J = 9.5 Hz*, H3’’ **11b**), 3.55 (1H, d, *J = 9.0 Hz*, H3’’ **10b**), 3.13 (3H, s, CO2C*H*3 **10b**), 3.04 (3H, s, CO2C*H*3 **11b**), 1.72 (3H, s, 2’’-C*H*3 **11b**), 1.62 (3H, s, 2’’-C*H*3 **10b**); 13C NMR(CD3OD, 100 MHz)  179.4, 177.4, 176.8, 173.0, 172.7, 172.1, 158.5, 158.3, 130.1, 129.7, 128.7, 127.5, 127.1, 126.4, 121.3, 121.2, 111.2, 111.1, 68.1, 67.5, 62.8, 62.8, 58.4, 57.6, 56.3, 56.3, 52.5, 51.9, 51.5, 51.2, 49.9, 42.4, 40.6, 28.3, 22.4; m/z (ESI-MS, +ve ion) 431.1 (M(**10b)**Na)+, 409.2 (M(**10b)**H)+, 399.1 (M(**11b)**Na)+, 377.2 (M(**11b)**H)+; *vmax*2965, 1700, 1685 cm-1; HRMS (LSIMS, +ve ion) Calculated for C19H24N2O8 (M+H)+ 409.1611. Found. 409.1607.

Data for **11c**: white solid 30 mg (HATU coupling 37%, deprotection 81 %). 1H NMR (CD3OD, 400 MHz)  6.91 – 6.84 (3H, m, Ar-H), 5.03 (1H, d, *J = 6.5 Hz*, H5’’), 4.15 (1H, d, *J = 17.0 Hz*, NC*H*H of glycine linker), 4.07 (1H, d, *J = 17.0 Hz*, NCH*H* of glycine linker), 3.81 (3H, s, OC*H*3), 3.75 (3H, s, OC*H*3), 3.69 (1H, dd, *J = 6.5 and 9.5 Hz*, H4’’), 3.50 (1H, d, *J = 9.5 Hz*, H3’’), 3.01 (3H, s, CO2C*H*3), 1.53 (3H, s, 2’’-C*H*3); 13C NMR (CD3OD, 100 MHz)  179.5, 176.8, 172.5, 170.5, 153.6, 148.0, 131.7, 124.7, 119.3, 113.8, 67.4, 62.6, 61.1, 56.4, 55.9, 52.6, 51.9, 40.6, 22.7; m/z (ESI, +ve ion) 475.1 (MNa3 – H)+, 453.1 (MNa2)+, 429.1 (MNa)+, 407.2 (MH)+; *vmax* 3335, 2949, 1705, 841 cm-1.

Data for **11d**: off-white solid 63 mg (HATU coupling 76%, deprotection 100%). 1H NMR (CD3OD, 400 MHz)  6.88 (1H, d, *J = 2.0 Hz*, Ar-Ha), 6.82 – 6.75 (2H, m, Ar-Hb and Ar-Hc), 4.79 (1H, d, *J = 6.0 Hz*, H5’’), 4.19 (1H, d, *J = 17.0 Hz*, NC*H*H of glycine linker), 4.11 (1H, d, *J = 17.0 Hz*, NCH*H* of glycine linker), 3.73 (6H, s, 2 x OC*H*3), 3.58 (1H, dd, *J = 6.0, 9.0 Hz*, H4’’), 3.40 (1H, d, *J = 9.0 Hz*, H3’’), 3.12 (3H, s, CO2C*H*3), 1.56 (3H, s, 2’’-C*H*3); 13C NMR (CD3OD, 100 MHz)  179.5, 176.7, 172.7, 170.4, 150.1, 137.91, 130.9, 126.4, 120.5, 112.4, 67.8, 67.6, 56.8, 56.7, 56.0, 54.1, 52.5, 40.8, 23.1; m/z (ESI, +ve ion) 451.1 (MNa2)+, 429.1 (MNa)+, 407.2 (MH)+; *vmax* 3651, 2962, 1703, 1014 and 796 cm-1.

Data for **10e**: white solid 62 mg (HATU coupling 69%, deprotection 100 %). 1H NMR(CD3OD, 400 MHz)  4.16 – 3.67 (12H, m, CH2 2’’’, H1’’’, H5’’, NHC*H*2 of glycine linker, CO2C*H*3, CO2C*H*3), 3.39 (1H, dd, *J = 9.5, 8.0 Hz*, H4’’), 3.13 (1H, d, *J = 8.0 Hz*, H3’’), 1.48 (1.7H, s, 2’’-C*H*3), 1.45 (1.3H, s, 2’’-C*H*3), 1.27 and 1.23 (3H, 2 x s, C(C*H*3)2), 1.17 and 1.16 (3H, 2 x s, C(C*H*3)2); m/z(ESI, +ve ion) 425.1 (MNa)+, 403.2 (MH)+, 345.2 (MH - OC(CH3)2)+; *vmax* 2962, 1710 cm-1; HRMS(LSIMS, +ve ion) Calculated for C17H26N2O9 (M+H)+ 403.1716; Found 403.1707.

**13b.1**;Melting point 197 – 200 ˚C.1H NMR(CD3OD, 500 MHz)  7.61 (1H, d, *J = 8.0 Hz*, H6), 7.55 (1H, d, *J = 7.5 Hz*, Ar-H), 7.53 – 7.36 (4H, m, Ar-H), 7.23 – 7.17 (2H, m, Ar-H), 7.12 – 7.06 (2H, m, Ar-H), 5.75 (1H, d, *J = 4.5 Hz*, H1’), 5.71 (1H, d, *J = 8.0 Hz*, H5), 5.46 (1H, d, *J = 10.0 Hz*, H5’’), 4.38 – 3.97 (7H, m, H2’, H3’, H4’, H4’’, H3’’, NHC*H*2 of glycine linker), 3.83 (3H, s, OC*H*3), 3.57 (2H, d, *J = 5.0 Hz*, C*H*2 5’), 1.97 (3H, s, 2’’-C*H*3); 13C NMR(CD3OD, 125 MHz)  174.3, 173.1, 172.4, 170.0, 169.0, 167.3, 164.7, 157.5, 156.4, 150.8, 141.6, 141.3, 136.5, 131.7, 130.9, 130.7, 128.6, 128.4, 126.2, 126.1, 124.9, 121.2, 120.7, 120.3, 118.3, 110.9, 110.6, 101.7, 101.5, 90.5, 90.1, 82.4, 82.3, 73.4, 70.8, 70.5, 69.4, 67.4, 62.8, 55.1, 55.0, 54.3, 48.2, 48.1, 43.2, 41.4, 40.7, 40.4, 24.9, 19.7; m/z (ESI, +ve ion) 701.2 (MK)+, 685.2 (MNa)+, 663.2 (MH)+; *vmax* 3345, 1668, 1179, 1130 cm-1; HRMS(micrOTOF, +ve ion) Calculated for C32H35N6O10 (M+H)+ 663.2409. Found 663.2424.

**13b.2**; Melting point 160 – 164 ˚C. 1H NMR(CD3OD, 500 MHz)  7.60 (0.5H, d, *J = 8.0 Hz*, H6), 7.56 – 7.51 (1H, m, Ar-H), 7.46 (0.5H, d, *J = 8.0 Hz*, H6), 7.41 – 7.36 (1H, m, Ar-H), 7.23 – 7.12 (2H, m, Ar-H), 7.12 – 7.05 (4H, m, Ar-H), 6.99 – 6.93 (1H, m, Ar-H), 5.78 – 5.77 (1H, m, H5, H1’), 5.75 (0.5H, d, *J = 4.5 Hz*, H1’), 5.68 – 5.67 (1.5H, m, H5, H5’’), 4.36 (0.5H, d, *J = 16.5 Hz*, NHC*H*H of glycine linker), 4.25 (1.5H, m, NHC*HH* of glycine linker), 4.20 – 4.17 (1H, m, H4’’), 4.14 (0.5H, dd, *J = 4.5, 5.5 Hz*, H2’), 4.11 (0.5H, dd, *J = 4.5, 5.5 Hz*, H2’), 4.07 – 4.05 (1H, m, H3’’), 4.02 – 3.95 (5H, m, H3’, H4’, OC*H*3), 3.53 (2H, m, C*H*2 5’), 1.97 (3H, s, 2’’-C*H*3); 13C NMR (CD3OD, 125 MHz)  173.1, 172.8, 172.4, 169.1, 168.9, 167.3, 167.1, 164.6, 164.4, 156.4, 150.9, 150.8, 141.7, 141.3, 136.5, 136.5, 130.7, 128.4, 128.3, 126.3, 126.2, 125.0, 124.9, 120.9, 120.7, 120.3, 118.3, 110.6, 101.7, 101.7, 90.7, 90.2, 82.4, 82.1, 73.4, 73.3, 70.9, 70.6, 67.4, 67.4, 62.9, 62.8, 55.0, 52.9, 49.4, 49.2, 41.4, 41.1, 40.5, 40.4, 19.7, 19.6; m/z (ESI, +ve ion) 701.2 (MK)+, 685.3 (MNa)+, 663.2 (MH)+; *vmax* 3309, 1663, 1176, 1139 cm-1; HRMS (mircrOTOF, +ve ion) Calculated for C32H35N6O10 (M+H)+ 663.2421. Found 663.2409.

Data for **13c**. White solid (12 mg, combined coupling/deprotection, 11%) isolated in a 2:1 ratio of diastereoisomers **a** and **b**). HPLC retention time 20.53 min. 1H NMR (CD3OD, 500 MHz)  7.62 (0.6H, d, *J = 8.0 Hz*, H6 **a** or **b**), 7.57 (0.4H, d, *J = 8.0 Hz*, H6 **a** or **b**), 7.47 – 7.28 (2H + 2H, m, H6 **a** or **b**, Ar-H **a** or **b**), 7.22 - 6.98 (7H + 6H, m, Ar-H **a** and **b**), 5.77 – 5.70 (2H + 1H, m, H1’ **a** and **b**, H5 **a**), 5.63 (1H, d, *J = 8.0 Hz*, H5 **b**), 5.57 - 5.56 (1H, m, H5’’ **a**), 5.27 (1H, d, *J = 9.5 Hz*, H5’’ **b**), 4.33 (1H, d, *J = 16.5 Hz*, NHC*H*H **b**), 4.24 – 3.88 (9H + 10H, m, H4’’ **a** and **b**, H3’’ **a** and **b**, NHCH*H* **b**, NHC*HH* **a**, H2’ **a** and **b**, H3’ **a** and **b**, H4’ **a** and **b**, OC*H*3 **a** and **b**), 3.85 (3H, s, OC*H*3 **b**), 3.82 (3H, s, OC*H*3 **a**), 3.62 – 3.38 (2H + 2H, m, C*H*2 5’ **a** and **b**), 1.89 (3H, s, 2’’-C*H*3 **a**), 1.78 (3H, s, 2’’-C*H*3 **b**); 13C NMR(CD3OD, 125 MHz)  174.8, 174.5, 172.0, 170.4, 168.9, 168.7, 166.2, 154.0, 153.6, 152.4, 152.2, 148.5, 147.9, 143.1, , 142.8, 138.0, 133.3, 132.3, 130.0, 129.8, 129.8, 129.7, 127.3, 126.3, 125.4, 124.9, 122.3, 122.1, 120.8, 119.4, 115.3, 114.4, 103.2, 103.1, 102.9, 92.1, 91.5, 83.9, 83.5, 74.9, 74.7, 72.3, 72.1, 71.9, 68.9, 64.0, 63.9, 61.6, 61.4, 56.4, 54.7, 51.9, 51.8, 49.7, 49.5, 49.3, 42.8, 42.6, 42.0, 41.9, 22.5, 21.4;m/z (ESI, +ve ion) 731.2 (MK)+, 715.2 (MNa)+, 693.2 (MH)+; *vma***x** 3066, 1663, 1177, 1131, 1079 cm-1; HRMS (micrOTOF, +ve ion) Calculated for C33H36N6O11 (M+H)+ 693.2515. Found 693.2528.

Data for **13e**. Yellow solid (43 mg, combined coupling/deprotection 25%) isolated as a 9:1 ratio of diastereoisomers **a** and **b**. HPLC retention time 15.02 min. Melting point 139 – 140 ˚C. 1H NMR(CD3OD, 500 MHz)  7.77 – 7.75 (1H, d, *J = 8.0 Hz*, H6 **b**) 7.63 – 7.62 (1H, d, *J = 8.0 Hz*, H6 **a**), 7.52 – 7.49 (2H + 2H, m, Ar-H **a** and **b**), 7.38 – 7.33 (2H + 2H, m, Ar-H **a** and **b**), 7.21 – 7.18 (1H + 1H, m, Ar-H **a** and **b**), 5.93 – 5.92 (1H, d, *J = 4.5 Hz*, H1’ **b**), 5.79 – 5.77 (1H + 2H, m, H1’ **a**, H5 **a** and **b**), 4.36 - 4.32 (1H, m, H5’’ **a** and **b**), 4.28 – 4.18 (3H + 3H, m, H2’ **a** and **b**, NHC*H*2 of glycine linker **a** and **b**), 4.03 – 3.97 (4H + 4H, m, H3’ **a** and **b**, H4’ **a** and **b**, H1’’’ **a** and **b**, H3’’ **a** and **b**), 3.85 (1H + 1H, dd, *J = 5.5, 9.0 Hz*, H4’’ **a** and **b**), 3.78 – 3.68 (2H + 2H, m, CH2 2’’’ **a** and **b**), 3.60 (1H + 1H, dd, *J = 6.0, 14.0 Hz*, C*H*H 5’ **a** and **b**), 3.47 (1H + 1H, dd, *J = 4.0, 14.0 Hz*, CH*H* 5’ **a** and **b**), 1.90 (3H + 3H, s, 2’’-C*H*3 **a** and **b**); 13C NMR (CD3OD, 125 MHz)  172.7, 172.6, 172.1, 171.7, 168.8, 168.4, 168.3, 167.0, 164.6, 164.7, 150.9, 141.8, 141.0, 137.1, 136.9, 128.6, 128.5, 126.6, 126.3, 120.7, 120.4, 101.7, 101.7, 90.7, 89.6, 83.0, 82.2, 73.4, 73.2, 70.8, 70.7, 68.8, 67.5, 67.3, 63.4, 53.8, 46.7, 46.6, 44.0, 41.0, 40.6, 39.3, 20.3, 20.1; m/z (ESI, +ve ion) 655.1 (MK)+, 639.2 (MNa)+, 617.2 (MH)+; *vmax* 3295, 1662, 1183, 1130 cm-1; HRMS (micrOTOF, +ve ion) Calculated for C27H32N6O11 (M+H)+ 617.2207. Found 617.2208.

Data for **14a**. White solid (16 mg, coupling 31%, deprotection 74%) isolated as a 1:1 ratio of diastereoisomers **a** and **b**). HPLC retention time 19.77 min. 1H NMR(D2O, 400 MHz)  7.70 (1H + 1H, d, *J = 8.0 Hz*, H6 **a** and **b**), 7.55 – 7.53 (3H + 3H, m, Ar-H **a** and **b**), 7.47 – 7.45 (2H + 2H, m, Ar-H **a** and **b**), 5.91 (1H + 1H, d, *J = 8.0 Hz*, H5 **a** and **b**), 5.82 (1H + 1H, d, *J = 4.0 Hz*, H1’ **a** and **b**), 5.48 (1H + 1H, d, *J = 10.0 Hz*, H5’’**a** and **b**), 4.46 (1H + 1H, dd, *J = 10.0, 10.0 Hz*, H4’’ **a** and **b**), 4.39 (1H + 1H, dd, *J = 4.0, 4.0 Hz*, H2’ **a** and **b**), 4.27 (1H + 1H, d, *J = 10.0 Hz*, H3’’ **a** and **b**), 4.21 – 4.16 (3H + 3H, m, NHC*H*H of glycine linker **a** and **b**, H3’ **a** and **b**, H4’ **a** and **b**), 4.09 (1H + 1H, d, *J = 17.0 Hz*, NHCH*H*of glycine linker **a** and **b**), 3.92 (3.5H + 3.5H, s, CO2C*H*3 **a** and **b**, C*H*H 5’ **a** and **b**), 3.88 – 3.87 (0.5H + 0.5H, m, C*H*H 5’ **a** and **b**), 3.56 (1H + 1H, dd, *J = 7.5, 14.5 Hz*, CH*H* 5’ **a** and **b**), 3.35 (3H + 3H, s, CO2C*H*3 **a** and **b**), 1.84 (3H + 3H, s, 2’’-C*H*3 **a** and **b**); 13C NMR(D2O, 100 MHz)  170.7, 170.7, 169.7, 166.2, 166.2, 151.3, 141.9, 132.1, 129.9, 129.2, 127.2, 101.8, 90.6, 81.9, 73.4, 70.4, 68.8, 61.3, 53.7, 52.6, 52.5, 50.0, 43.4, 40.9, 17.9; m/z 642.1 (MK)+, 626.2 (MNa)+, 604.3 (MH)+; *vmax* 3293, 2980, 1662 cm-1; HRMS Calculated for C27H33N5O11 (M+H)+ 604.2255. Found 604.2242.

Data for **14b**. Coupling and deprotection gave two isomers of **14b** (12 mg, 22mg), which were separable by HPLC, and bicyclic **15b** (17 mg), isolated as a mixture of diastereoisomers. Combined overall yield 23%. **14b isomer 1**. HPLC retention time 17.48 min. 1H NMR(CD3CN/D2O, 500 MHz)  7.56 (1H, d, *J = 8.0 Hz*, H6), 7.42 (1H, ddd, *J = 1.5, 8.5, 8.5 Hz*, Ar-Hc), 7.26 (1H, dd, *J = 1.5, 7.5 Hz*, Ar-Ha), 7.02 – 6.99 (2H, m, Ar-H b and d), 5.77 (1H, d, *J = 8.0 Hz*, H5), 5.66 (1H, d, *J = 4.0 Hz*, H1’), 5.30 (1H, d, *J = 9.5 Hz*, H5’’), 4.20 – 4.13 (3H, m, H2’, H4’’, H3’’), 4.00 – 3.96 (4H, m, NHC*H*2 of glycine linker, H3’, H4’), 3.76 (3H, s, OC*H*­3), 3.74 (3H, s, CO2C*H*3), 3.53 (1H, dd, *J = 2.5, 14.5 Hz*, C*H*H 5’), 3.45 (1H, dd, *J = 6.5, 14.5 Hz*, CH*H* 5’) 3.15 (3H, s, CO2C*H*3), 1.66 (3H, s, 2’’-C*H*3); 13C NMR (CD3CN, 125 MHz)  171.1, 170.7, 170.5, 170.1, 166.3, 157.5, 152.1, 142.9, 133.1, 132.1, 122.2, 119.2, 112.3, 103.1, 91.4, 82.5, 74.1, 71.5, 69.1, 60.8, 56.0, 54.4, 54.3, 53.3, 48.9, 44.1, 41.8, 15.9; m/z (ESI, +ve ion) 672.3 (MK)+, 656.2 (MNa)+, 634.2 (MH)+; *vmax*3300, 1664, 1544, 1133 cm-1; HRMS (LSIMS, +ve ion) Calculated for C28H35N5O12 (M+H)+ 634.2360. Found 634.2359. **14b isomer 2**. HPLC retention time 16.97 min. 1H NMR (CD3CN/D2O, 500 MHz)  7.51 (1H, d, *J = 8.0 Hz*, H6), 7.43 (1H, ddd, *J = 1.5, 8.5, 8.5 Hz*, Ar-Hc), 7.27 (1H, dd, *J = 1.5, 7.5 Hz*, Ar-Ha), 7.04 – 6.99 (2H, m, Ar-H b and d), 5.72 (1H, d, *J = 8.0 Hz*, H5), 5.68 (1H, d, *J = 4.0 Hz*, H1’), 5.34 – 5.29 (1H, m, H5’’), 4.18 – 4.14 (3H, m, H2’, H4’’, H3’’), 3.98 – 3.93 (4H, m, NHC*H*2 of glycine linker, H3’, H4’), 3.80 (3H, s, OC*H*3), 3.76 (3H, s, CO2C*H*3), 3.14 (3H, s, CO2C*H*3), 1.68 (2’’-C*H*3); 13C NMR (CD3CN/D2O, 125 MHz)  170.6, 170.3, 170.3, 169.8, 165.7, 157.7, 151.9, 142.7, 132.9, 132.3, 122.7, 119.6, 112.4, 103.0, 95.2, 83.0**,** 74.3, 71.6, 69.2, 60.7, 56.1, 54.4, 54.3, 53.0, 49.0, 44.1, 41.8, 15.8; m/z (ESI, +ve ion) 656.2 (MNa)+, 634.2 (MH)+; *vmax* 3292, 2960, 1664 cm-1; HRMS(LSIMS, +ve ion) Calculated for C28H35N5O12 (M+H)+ 634.2360. Found 634.2379. Data for **15b**, isolated as a 7:3 ratio of diastereoisomers **c** and **d**). HPLC retention time 18.61 minutes 1H NMR (CD3CN, 500 MHz)  7.49 – 7.47 (1H + 1H, m, H6 **c** and **d**), 7.38 – 7.26 (2H + 2H, m, Ar-H **c** and **d**), 7.01 – 6.90 (2H + 2H, m, Ar-H **c** and **d**), 5.72 – 5.69 (1H + 1H, m, H5 **c** and **d**), 5.66 – 5.65 (1H + 1H, m, H1’ **c** and **d**), 5.09 (1H, d, *J = 9.0 Hz*, H5’’ **d**), 5.02 (1H, d, *J = 9.0 Hz*, H5’’ **c**), 4.15 – 4.11 (3H + 3H, m, NC*H*2 of glycine linker **c** and **d**, H2’ **c** and **d**), 3.93 – 3.92 (2H + 2H, m, H3’ **c** and **d**, H4’ **c** and **d**), 3.73 – 3.63 (8H + 8H, m, OC*H*3 **c** and **d**, CO2C*H*3 **c** and **d**, H3’’ **c** and **d**, C*H*H 5’ **c** and **d**), 3.48 – 3.44 (2H + 2H, m, H4’’ **c** and **d**, CH*H* 5’ **c** and **d**), 1.69, 1.65 (3H + 3H, s, 2’’-C*H*3 **c** and **d**); 13C NMR(CD3CN, 125 MHz)  177.4, 176.2, 172.8, 168.1, 165.6, 158.1, 152.0, 142.7, 131.4, 129.2, 124.7, 121.7, 112.0, 103.1, 91.3, 82.9, 74.2, 71.3, 68.1, 63.9, 56.1, 55.2, 53.6, 52.0, 41.9, 41.5, 20.1; m/z (ESI, +ve ion) 624.1 (MNa)+, 602.2 (MH)+; *vmax*3293, 2960, 1669, 1182 and 1131 cm-1; HRMS(micrOTOF, +ve ion) Calculated for C27H31N5O11 (M+H)+ 602.2093. Found 602.2099.

Data for **15e**. White solid (12 mg, combined coupling & deprotection 25%) isolated as a 1:1 ratio of diastereoisomers **a** and **b**). HPLC retention time 10.80 min. 1H NMR (CD3OD, 500 MHz)  .69 – 7.67 (1H + 1H, m, H6 **a** and **b**), 5.83 – 5.74 (2H + 2H, m, H5 **a** and **b**, H1’ **a** and **b**), 4.47 – 4.15 (3H + 3H, m, NC*H*2 **a** and **b**, H2’ **a** and **b**, H1’’’ **a** and **b**), 4.09 – 3.92 (2H + 2H, m, H3’ **a** and **b**, H4’ **a** and **b**), 3.89 – 3.74 (6H + 6H, m, CO2C*H*3 **a** and **b**, CH2 2’’’ **a** and **b**, H4’’ **a** and **b**), 3.72 – 3.52 (4H + 4H, m, C*H*2 5’ **a** and **b**, H5’’ **a** and **b**, H3’’ **a** and **b**), 1.99, 1.91, 1.85, 1.81, 1.80, 1.77 (3H + 3H, 6 x s, 2’’-C*H*3 **a** and **b**); 13C NMR (CD3OD, 125 MHz)  177.2, 176.3, 173.3, 168.5, 166.3, 152.1, 142.9, 103.1, 91.1, 82.6, 74.1, 71.9, 70.6, 69.2, 66.6, 64.2, 54.3, 54.2, 49.7, 49.4, 42.1, 41.4, 20.9, 20.6; m/z(ESI, +ve ion) 578.2 (MNa)+, 556.2 (MH)+; *vmax* 3300, 2980, 1663, 1183, 1130 cm-1; HRMS (micrOTOF, +ve ion)Calculated for C22H29N5O12 (M+H)+ 556.1885. Found 556.1897.

Data for **16a**. White solid (62 mg, coupling 33%, deprotection 75%) isolated as 1:1 ratio of diastereoisomers **a** and **b**. HPLC retention time 20.21 min. 1H NMR(CD3OD, 500 MHz)  7.65 – 7.63 (1H + 1H, 2 x overlapping d, *J = 7.5 and 7.5 Hz*, H6 **c** and **d**), 7.42 – 7.36 (5H + 5H, m, Ar-H **c** and **d**), 5.79 (1H + 1H, d, *J = 4.5 Hz*, H1’ **c** and **d**), 5.77 (1H, d, *J = 7.5 Hz*, H5 **c** or **d**), 5.73 (1H, d, *J = 8.0 Hz*, H5 **c** or **d**), 5.32 – 5.29 (1H + 1H, 2 x overlapping d, *J = 7.5 and 7.5 Hz*, H5’’ **c** and **d**), 4.33 – 4.17 (3H + 3H, m, NC*H*2 **c** and **d**, H2’ **c** and **d**), 4.05 – 3.99 (2H + 2H, m, H3’ **c** and **d**, H4’ **c** and **d**), 3.94 – 3.90 (1H + 1H, m, H4’’ **c** and **d**), 3.81 (1H, d, *J = 9.5 Hz*, H3’’ **c** or **d**), 3.79 (1H, d, *J = 9.5 Hz*, H3’’ **c** or **d**), 3.64 – 3.56 (2H + 2H, m, C*H*2 5’ **c** and **d**), 3.25 (3H, s, CO2CH3 **c** or **d**), 3.24 (3H, s, CO2C*H*3 **c** or **d**), 1.80 (3H + 3H, s, 2’’-C*H*3 **c** and **d**); 13C NMR (CD3OD, 125 MHz)  176.2, 176.0, 175.4, 175.2, 172.7, 172.6, 168.8, 168.8, 166.2, 152.4, 143.6, 143.3, 134.9, 134.6, 130.2, 130.1, 129.9, 129.8, 128.6, 127.8, 103.2, 103.1, 92.2, 92.1, 83.9, 83.9, 74.7, 74.7, 72.2, 72.1, 68.3, 68.3, 68.0, 54.8, 52.8, 52.7, 49.5, 49.3, 42.5, 42.4, 42.1, 41.9, 21.4, 21.3; m/z (ESI, +ve ion) 610.1 (MK)+, 594.2 (MNa)+, 572.2 (MH)+; *vmax* 3294, 2956, 1665, 1560 cm-1; HRMS(LSIMS, +ve ion) Calculated for C26H29N5O10 (M+H)+ 572.1993. Found 572.1997.

Data for **16b**. White solid (13 mg, coupling 32%, deprotection 91%) isolated as a 1:1 ratio of diastereoisomers **c** and **d**). HPLC retention time 20.76 min. 1H NMR(CD3OD, 500 MHz)  7.67 – 7.65 (1H + 1H, 2 x overlapping dd, *J = 8.0 and 8.0 Hz*, H6 **c** and **d**), 7.40 – 7.27 (2H + 2H, m, Ar-H **c** and **d**), 7.05 – 7.02 (1H + 1H, 2 x overlapping d, *J = 8.0 and 8.0 Hz*, Ar-H **c** and **d**), 6.97 – 6.93 (1H + 1H, dd, *J = 7.5 and 7.5 Hz*, Ar-H **c** and **d**), 5.82 – 5.80 (1H + 1H, m, H1’ **c** and **d**), 5.80 (1H, d, *J = 8.0 Hz*, H5 **c** or **d**), 5.76 (1H, d, *J = 8.0 Hz*, H5 **c** or **d**), 5.36 – 5.33 (1H + 1H, m, H5’’ **c** and **d**), 4.32 (1H, d, *J = 16.5 Hz*, NC*H*H of glycine linker **c** or **d**), 4.24 – 4.20 (1H + 3H, m, NC*HH* **c** or **d**, H2’ **c** and **d**), 4.17 (1H, d, *J = 16.5 Hz*, NHCH*H* of glycine linker **c** or **d**) 4.07 – 4.01 (3H + 3H, m, H4’’ **c** and **d**, H3’ **c** and **d**, H4’ **c** and **d**), 3.78 – 3.74 (1H + 1H, 2 x overlapping d, *J = 9.5 and 9.5 Hz*, H3’’ **c** and **d**), 3.66 – 3.58 (2H + 2H, m, C*H*2 5’ **c** and **d**), 3.15 (3H, s, CO2C*H*3 **c** or **d**), 3.13 (3H, s, CO2C*H*3 **c**/**d**), 1.77, 1.76 (3H + 3H, s, 2’’-C*H*3 **c** and **d**); 13C NMR (CD3OD, 125 MHz)  (quarternary C were not seen) 141.8, 129.5, 125.5, 120.0, 110.1, 101.8, 101.7, 90.7, 90.4, 82.5, 82.4, 73.7, 73.2, 70.7, 70.6, 61.6, 54.7, 53.5, 51.0, 49.3, 41.0, 40.9, 40.6, 40.4, 20.2; m/z (ESI, +ve ion) 624.2 (MNa)+, 602.2 (MH)+; *vmax* 3274, 2952, 1670 cm-1; HRMS (micrOTOF, +ve ion) Calculated for C27H31N5O11 (M+H)+ 602.2098. Found 602.2116.

Data for **16c**. White solid (10 mg, combined coupling and deprotection, 19%) isolated in 1:1 ratio of diasterioisomers **c** and **d**). HPLC retention time 20.49 min. 1H NMR (CD3OD, 500 MHz)  7.65 (1H, d, *J = 8.0 Hz*, H6 **c**/**d**), 7.62 (1H, d, *J = 8.0 Hz*, H6 **c**/**d**), 7.06 – 7.04 (2H + 2H, m, Ar-H **c** and **d**), 7.00 – 6.97 (1H + 1H, m, Ar-H **c** and **d**), 5.79 (1H, d, *J = 4.5 Hz*, H1’ **c** or **d**), 5.78 (1H, d, *J = 4.5 Hz*, H1’ **c** or **d**), 5.76 (1H, d, *J = 8.0 Hz*, H5 **c** or **d**), 5.73 (1H, d, *J = 8.0 Hz*, H5 **c** or **d**), 5.43 (1H, d, *J = 6.5 Hz*, H5’’**c** or **d**), 5.41 (1H, d, *J = 6.5 Hz*, H5’’ **c** or **d**), 4.30 (1H, d, *J = 16.5 Hz*, NC*H*H of glycine linker **c** or**d**), 4.25 – 4.17 (2H + 3H, m, NCH*H* of glycine linker **c** or **d**, NC*HH* of glycine linker **c** or **d**, H2’ **c** and **d**), 4.05 – 3.95 (3H + 3H, m, H3’ **c** and **d**, H4’ **c** and **d**, H4’’ **c** and **d**), 3.95 (3H + 3H, s, OC*H*3 **c** and **d**), 3.87 (3H + 3H, s, OC*H*3 **c** and **d**), 3.83 (1H + 1H, d, *J = 9.5 Hz*, H3’’ **c** and **d**), 3.61 – 3.59 (2H + 2H, m, C*H*2 5’ **c** and **d**), 3.22 (3H, s, CO2C*H*3 **c** or **d**), 3.21 (3H, s, CO2C*H*3 **c**/**d**), 1.79 (3H + 3H, s, 2’’-C*H*3 **c** and **d**); 13C NMR (CD3OD, 176 MHz)  175.7, 175.4, 172.6, 172.4, 168.9, 168.9, 166.1, 153.9, 152.4, 148.0, 143.3, 125.1, 125.1, 121.5, 118.7, 118.6, 114.8, 114.7, 103.2, 103.2, 92.2, 92.0, 83.9, 83.9, 74.7, 74.7, 72.2, 72.1, 68.0, 67.9, 63.1, 61.4, 61.4, 56.4, 54.8, 52.7, 51.4, 42.5, 42.4, 42.1, 41.9, 21.4, 21.3; m/z (ESI, +ve ion) 670.1 (MK)+, 654.3 (MNa)+, 632.2 (MH)+; HRMS (micrOTOF, +ve ion) Calculated for C28H33N5O12 (M+H)+ 632.2198. Found 632.2214.

Data for **16d**. White solid (9 mg, combined coupling and deprotection, 9%) isolated as a 1:1 ratio of diastereoisomers **c** and **d**. HPLC retention time 17.31 min. 1H NMR(CD3OD, 500 MHz)  7.65 – 7.63 (1H + 1H, 2 x d, *J = 8.0 and 8.0 Hz*, H6 **c** and **d**), 7.05 (1H + 1H, s, Ar-H **c** and **d**), 6.98 – 6.94 (2H + 2H, m, Ar-H **c** and **d**), 5.79 – 5.76 (1H + 2H, m, H5 **c** or **d**, H1’ **c** and **d**), 5.73 (1H, d, *J = 8.0 Hz*, H5 **c** or **d**) 5.24 (1H, d, *J = 6.5 Hz*, H5’’ **c** or **d**), 5.22 (1H, d, *J = 6.5 Hz*, H5’’ **c** or **d**), 4.33 – 4.17 (3H + 3H, m, NC*H*2 **c** and **d**, H2’ **c** and **d**), 4.05 – 3.99 (2H + 2H, m, H3’ **c** and **d**, H4’ **c** and **d**), 3.88 – 3.76 (8H + 8H, m, H4’’ **c** and **d**, H3’’ **c** and **d**, OC*H*3 **c** and **d**, OC*H*3 **c** and **d**), 3.64 – 3.55 (2H + 2H, m, C*H*2 5’ **c** and **d**), 3.35 (3H + 3H, s, CO2C*H*3 **c** and **d**), 1.79 (3H + 3H, s, 2’’-C*H*3 **c** and **d**); 13C NMR (CD3OD, 125 MHz)  175.4, 175.2, 172.8, 172.7, 168.8, 166.1, 152.4, 151.2, 150.7, 143.3, 120.9, 112.8, 111.6, 103.2, 103.1, 92.2, 83.9, 74.7, 72.1, 68.1, 67.9, 56.6, 56.5, 54.7, 52.9, 52.7, 52.6, 49.5, 49.3, 42.4, 42.1, 42.0, 21.4, 21.3; m/z (ESI, +ve ion) 670.1 (MK)+, 654.3 (MNa)+, 632.2 (MH)+; HRMS(micrOTOF, +ve ion) Calculated for C28H33N5O12 (M+Na)+654.2018. Found 654.2033.

Data for **18**. 1H NMR (CD3OD, 400 MHz)  7.61 – 6.91 (9H, m, Ar-H), 4.69 – 4.61 (1H, m, H5’’), 4.24 – 4.06 (1H, m, H2’’), 3.88 – 3.67 (7H, m, OC*H*3, H4’’, H3’’, C*H*2 of sarcosine linker), 3.34 (3H, s, NC*H*3 of sarcosine linker), 2.18 (3H, s, N1’’-C*H*3); 13C NMR (CD3OD, 100 MHz)  not all quarternary C’s were seen) 177.1, 176.4, 159.3, 133.6, 130.3, 130.1, 129.9, 129.6, 129.6, 129.5, 127.5, 111.4, 69.4, 66.6, 56.0, 51.1, 46.9, 40.3, 36.6; m/z (ESI, +ve ion) 496.1 (MNa2 – H)+, 490.1 (MK)+, 474.2 (MNa)+, 452.2 (MH)+.

Data for **19**. HPLC retention time 20.43 min. A mixture of *cis* and *trans* N-methyl amide rotamers was observed by NMR spectroscopy. 1H NMR(CD3OD, 500 MHz)  7.72 – 7.30 (7.5H, m, Ar-H, H6), 7.21 – 7.13 (2H, m, Ar-H), 7.00 (0.5H, m, Ar-H), 5.83 – 5.73 (1.5H, m, H5, H1’), 5.41 – 5.38 (0.5H, m, H1’, H5’’), 5.27 – 5.19 (1H, m, H5’’), 4.66 – 3.49 (13H, m, H2’, H3’, H4’, C*H*2 5’, H2’’, H5’’, H3’’, OC*H*3, N(CH3)C*H*2), 3.40 - 3.38 (2H, m, NC*H*3 of sarcosine linker), 3.15 (1H, s, NC*H*3 of sarcosine linker), 2.94 – 2.93 (2H, m, N1’’-C*H*3), 2.27 – 2.26 (1H, m, N1’’-C*H*3); 13C NMR (CD3OD, 125 MHz)  171.3, 165.4, 164.7, 157.9, 157.1, 150.8, 132.4, 131.6, 128.8, 128.7, 128.3, 128.0, 127.5, 126.6, 126.1, 125.9, 122.1, 111.5, 110.0, 101.9, 101.6, 101.5, 91.6, 90.8, 90.4, 89.5, 82.9, 82.6, 82.5, 82.1, 81.9, 73.6, 73.3, 72.9, 72.1, 71.6, 71.1, 70.9, 70.8, 70.5, 68.1, 65.1, 55.6, 55.4, 54.6, 51.6, 51.5, 51.3, 45.7, 44.6, 41.2, 40.7, 40.4, 40.3, 38.9, 38.6, 35.6, 35.3, 34.9, 34.6; m/z(ESI, +ve ion) 715.1 (MK)+, 699.2 (MNa)+, 677.3 (MH)+; *vmax* 3350, 1713, 1661, 1167, 1130 cm-1. HRMS (MicrOTOF, +ve ion) Calculated for C33H37N6O10 (M+H)+ 677.2566. Found 677.2580.

*Data for* ***20a***. The product was purified by HPLC using a water/methanol gradient and **20a** was eluted at 17.89 minutes and concentrated to yield a white solid (25 mg; L-Pro coupling 91%, deprotection 100%, uridine coupling/deprotection 15%) isolated as a 1:1 ratio of diastereoisomers **a** and **b**. 1H NMR (CD3OD, 500 MHz)  7.66 (1H + 1H, d, *J = 8.0 Hz*, H6 **a** and **b**), 7.50 – 7.39 (5H + 5H, m, Ar-H **a** and **b**), 7.25 – 7.23 (2H + 2H, m, Ar-H **a** and **b**), 7.13 – 7.07 (2H + 2H, m, Ar-H **a** and **b**), 5.76 – 5.70 (2H + 2H, m, H1’ **a** and **b**, H5 **a** and **b**), 5.53 (1H + 1H, d, *J = 10.0 Hz*, H5’’ **a** and **b**), 4.44 (1H + 1H, dd, *J = 7.5, 9.5 Hz*, NC*H* of proline linker **a** and **b**), 4.29 – 4.16 (3H + 3H, m, H2’ **a** and **b**, H3’ **a** and **b**, H4’’ **a** and **b**), 4.15 – 3.95 (3H + 3H, m, NC*H*2 of proline linker **a** and **b**, H4’ **a** and **b**), 3.77 (1H + 1H, m, H3’’ **a** and **b**), 3.74 (3H + 3H, s, OC*H*3 **a** and **b**), 3.60 – 3.50 (2H + 2H, m, C*H*2 5’ **a** and **b**), 2.43 – 2.38 (1H + 1H, m, C*H*2 of proline linker **a** and **b** ­), 2.17 – 2.12 (2H + 2H, m, C*H*2 of proline linker **a** and **b**), 2.10 (3H + 3H, s, 2’’-C*H*3 **a** and **b**), 1.94 – 1.86 (1H + 1H, m, C*H*2 of proline linker **a** and **b**); 13C NMR (CD3OD, 125 MHz)  174.4, 174.3, 173.3, 173.1, 168.7, 166.1, 158.8, 152.4, 152.3, 143.6, 143.4, 133.2, 133.0, 132.7, 130.1, 129.9, 127.3, 123.1, 117.9, 112.8, 103.1, 103.0, 92.8, 83.9, 74.6, 72.4, 72.3, 64.7, 62.0, 56.9, 53.6, 51.6, 42.4, 30.7, 26.9, 21.3; m/z (ESI, +ve ion) 725.3 (MNa)+, 703.3 (MH)+; HRMS (ESI, +ve ion) Calculated for C35H38N6O10 (M+H)+ 703.2722. Found 703.2741.

Data for **20b**. The product was purified by HPLC using a water/methanol gradient and **20b** was eluted at 17.72 minutes and concentrated to yield a white solid (5 mg; L-Pro coupling 50%, deprotection 100%, uridine coupling/deprotection 5%), isolated as a 1:1 ratio of diastereoisomers **a** and **b**. 1H NMR (CD3OD, 500 MHz)  7.68 (1H + 1H, d, *J = 8.0 Hz*, H6 **a** and **b**), 7.47 (1H + 1H, dd, *J = 8.0, 8.0 Hz*, Ar-Hb **a** and **b**), 7.39 (1H + 1H, d, *J = 7.5 Hz*, Ar-Hd **a** and **b**), 7.11 (1H + 1H, d, *J = 8.0 Hz*, Ar-Ha **a** and **b**), 7.07 (1H + 1H, dd, *J = 8.0, 7.5 Hz*, Ar-Hc **a** and **b**), 5.74 – 5.72 (2H + 2H, m, H1’ **a** and **b**, H5 **a** and **b**), 5.47 (1H + 1H, d, *J = 8.0 Hz*, H5’’ **a** and **b**), 4.60 (1H + 1H, br t, *J = 6.5 Hz*, NC*H* of proline linker **a** and **b**), 4.25 (1H + 1H, dd, *J = 4.5, 4.5 Hz*, H2’ **a** and **b**), 4.18 (1H + 1H, d, *J = 2.0 Hz*, H3’’ **a** and **b**), 4.08 – 4.02 (3H + 3H, m, H3’ **a** and **b**, H4’ **a** and **b**, NC*H*H of proline linker **a** and **b**), 3.92 – 3.87 (4H + 4H, m, H4’’ **a** and **b**, OC*H*3 **a** and **b**), 3.82 (1H + 1H, m, NCH*H* of proline linker **a** and **b**), 3.76 (3H + 3H, s, CO2C*H*3 **a** and **b**), 3.74 (3H + 3H, s, CO2C*H*3 **a** and **b**), 3.61 – 3.44 (2H + 2H, m, C*H*2 5’ **a** and **b**), 2.36 – 1.93 (4H + 4H, m, C*H*2 of proline linker **a** and **b**), 1.80 (3H + 3H, s, 2’’-C*H*3 **a** and **b**); 13C NMR (CD3OD, 125 MHz)  (C=O and aryl quarternary C’s were not seen) 143.5, 132.8, 131.2, 122.5, 118.3, 103.0, 92.7, 83.9, 74.7, 72.4, 71.6, 64.4, 56.2, 53.6, 53.4, 53.4, 52.8, 52.5, 50.3, 42.2, 30.2, 26.9, 16.2; m/z (ESI, +ve ion) 712.2 (MK)+, 696.3 (MNa)+, 674.3 (MH)+; HRMS (ESI, +ve ion) Calculated for C31H39N5O12 (M+H)+ 674.2668. Found 674.2685.

Data for **20c**. The product was purified by HPLC using a water/methanol gradient and **20c** was eluted at 18.74 minutes and concentrated to yield a white solid (17 mg, L-Pro coupling 57%, deprotection 92%, uridine coupling/deprotection 17%), isolated as a 1:1 ratio of diastereoisomers **a** and **b**. 1H NMR (CD3OD, 500 MHz)  7.70 (1H, d, *J = 8.5 Hz*, H6 **a** or **b**), 7.68 (1H, d, *J = 8.5 Hz*, H6 **a** or **b**), 7.47 – 7.38 (2H + 2H, m, Ar-Hb/c and Ar-Ha/d **a** and **b**), 7.11 – 7.08 (1H + 1H, m, Ar-Ha/d **a** and **b**), 7.04 (1H + 1H, ddd, *J = 3.0, 8.0, 8.0 Hz*, Ar-Hb/c **a**  and **b**) 5.78 (1H, d, *J = 4.5 Hz*, H1’ **a** or **b**), 5.74 – 5.71 (1H + 2H, m, H1’ **a** or **b**, H5 **a** and **b**), 5.60 (1H, d, *J = 11.0 Hz*, H5’’ **a** or **b**), 5.48 (1H, d, *J = 10.0 Hz*, H5’’ **a** or **b**), 4.50 – 4.42 (1H + 2H, m, NC*H* of proline linker **a** and **b**, H4’’ **a** or **b**), 4.35 (1H, dd, *J = 10.0, 10.0 Hz*, H4’’ **a** or **b**), 4.25 (1H + 1H, dd, *J = 4.5, 9.5 Hz*, H2’ **a** and **b**), 4.09 – 3.99 (3H + 3H, m, H3’ **a** and **b**, H4’ **a** and **b**, H3’’ **a** and **b**), 3.89 (3H, s, OC*H*3 **a** or **b**), 3.88 (3H, s, OC*H*3 **a** or **b**), 3.87 (3H, s, CO2C*H*3 **a** or **b**), 3.83 – 3.78 (1H, m, NC*H*2 **a** or **b**), 3.75 - 3.70 (3H + 1H, m, CO2C*H*3 **a** or **b**, NC*H*2 **a** or **b**), 3.67 – 3.47 (3H + 3H, m, NC*H*2 **a** and/or **b**, C*H*2 5’ **a** and **b**), 3.22 (3H, s, CO2C*H*3 **a** or **b**), 3.17 (3H, s, CO2C*H*3 **a** or **b**), 2.32 – 2.27 (1H, m, C*H*2 of proline linker **a** or **b**), 2.24 – 2.19 (1H, m, C*H*2 of proline linker **a** or **b**), 2.12 – 2.08 (1H + 1H, m, C*H*2 of proline linker **a** and **b**), 2.03 – 1.98 (1H + 1H, m, C*H*2 of proline linker **a** and **b**), 1.94 – 1.89 (1H + 4H, m, C*H*2 of proline linker **a** and **b** and 2’’-C*H*3 **a** or **b**), 1.85 (3H, s, 2’’-C*H*3 **a** or **b**); 13C NMR (CD3OD, 125 MHz)  174.5, 173.6, 173.5, 172.0, 170.5, 169.8, 168.6, 166.1, 158.9, 158.7, 152.4, 143.5, 143.4, 132.7, 132.4, 130.0, 122.2, 120.3, 120.1, 112.7, 112.7, 103.0, 92.8, 92.4, 83.9, 83.9, 74.7, 74.6, 73.3, 72.5, 72.3, 71.9, 63.7, 63.5, 57.1, 56.5, 56.5, 55.4, 54.2, 53.6, 53.5, 52.7, 52.7, 52.3, 51.2, 50.8, 50.2, 42.4, 42.3, 30.3, 29.8, 26.8, 26.5, 21.0, 19.3; m/z (ESI, +ve ion) 712.2 (MK)+, 696.3 (MNa)+, 674.3 (MH)+; HRMS (ESI, +ve ion) Calculated for C31H39N5O12 (M+H)+ 674.2668. Found 674.2689.

Compound **20d** was prepared from a cycloaddition reaction of imine **1e** with N-ethyl maleimide (32% yield). The cycloadduct was debenzylated by hydrogenation (92% yield), coupled with L-proline benzyl ester (57% yield), debenzylated by hydrogenation (100% yield), coupled with 5’-amino-uridine derivative **12** and fully deprotected. The product was purified by HPLC using a water/methanol gradient and **20d** was eluted at 13.18 minutes and concentrated to yield a white solid (24 mg, 23%), isolated as 5:2 ratio of diastereomers **a** and **b**. 1H NMR (CD3OD, 500 MHz)  7.73 (1H, d, *J = 8.0 Hz*, H6 **b**), 7.68 (1H, d, *J = 8.0 Hz*, H6 **a**), 5.83 (1H, d, *J = 4.5 Hz*, H1’ **a**), 5.78 – 5.74 (1H + 2H, m, H1’ **b**, H5 **a** and **b**), 4.73 (1H, m, NC*H* of proline linker **b**), 4.52 (1H, dd, *J = 1.9, 9.0 Hz*, NC*H* of proline linker **a**), 4.40 (1H, dd, *J = 3.0, 10.0 Hz*, H1’’’ **b**), 4.36 (1H, dd, *J = 4.0, 10.0 Hz*, H1’’’ **a**), 4.30 (1H, dd, *J = 5.0, 5.0 Hz*, H2’ **b**), 4.23 (1H, dd, *J = 5.0, 5.0 Hz*, H2’ **a**), 4.18 – 4.14 (1H + 2H, m, H5’’ **a** and **b**, H4’’ **b**), 4.08 – 4.03 (2H + 2H, m, C*H* 3’ **a** and **b**, C*H* 4’ **a** and **b**), 4.02 – 3.43 (10H + 9H, m, C*H*2 2’’’ **a** and **b**, NC*H*2 of proline linker **a** and **b**, C*H*2 5’ **a** and **b**, C*H*2CH3 **a** and **b**, H4’’ **a**, H3’’ **a** and **b**), 2.56 – 2.47 (1H, m, C*H*H of proline linker **b**), 2.33 – 2.20 (1H, m, C*H*H of proline linker **a**), 2.15 – 2.11 (2H + 2H, m, C*H*2 of proline linker **a** and **b**), 2.10 – 1.90 (1H + 1H, m, CH*H* of proline linker **a** and **b**), 1.87 (3H, s, 2’’-C*H*3 **a**), 1.73 (3H, s, 2’’-C*H*3 **b**), 1.17 (3H, t, *J = 8.0 Hz*, CH2C*H*3 **a**), 1.13 (3H, t, *J = 8.0 Hz*, CH2C*H*3 **b**); 13C NMR (CD3OD, 125 MHz)  176.3, 174.9, 174.2, 167.9, 166.1, 152.2, 143.5, 142.9, 103.1, 103.0, 92.8, 91.9, 83.5, 83.4, 74.9, 74.6, 72.9, 72.7, 72.1, 71.8, 67.7, 66.9, 64.9, 64.8, 64.5, 63.6, 59.7, 58.9, 53.4, 52.7, 50.8, 46.5, 46.3, 42.8, 42.7, 35.9, 35.4, 33.4, 30.4, 25.9, 22.3, 21.6, 20.7, 12.6, 12.5; m/z(ESI, +ve ion) 631.3 (MNa)+, 609.3 (MH)+; *vmax* 3354, 2949, 1635, 1136 cm-1; HRMS (ESI, +ve ion) Calculated for C26H36N6O11 (M+H)+ 609.2515. Found 609.2573.

*Data for*  **22**. Melting point 104 – 107 ˚C. 1H NMR (CDCl3, 300 MHz)  7.78 (1H, d, *J = 7.5 Hz*, Ar-H), 7.53 – 7.50 (2H, m, Ar-H), 7.33 – 7.23 (3H, m, Ar-H), 7.16 – 7.11 (1H, m, Ar-H), 7.06 – 7.01 (1H, m, Ar-H), 6.86 (1H, d, *J = 8.5 Hz*, Ar-H), 5.04 (1H, dd, *J = 2.0, 3.5 Hz*), 4.90 (1H, d, *J = 12.0 Hz*), 4.67 (1H, br d, *J = 9.5 Hz*), 4.49 (1H, br d, *J = 12.0 Hz*), 3.81 (3H, s, OC*H*3), 3.72 (1H, dd, *J = 3.5, 5.5 Hz*), 3.01 (1H, dd, *J = 2.0, 5.5 Hz*), 1.92 (2H, br s, N*H*2), 1.39 (3H, s, 2’’-C*H*3); 13C NMR (CDCl3, 75 MHz)  171.9, 156.2, 137.2, 128.9, 128.7, 127.9, 127.0, 125.4, 120.9, 120.5, 109.2, 95.1, 88.1, 65.1, 57.1, 56.5, 55.2, 50.4, 19.5; m/z (ESI, +ve ion) 405.1 (MK)+, 389.1(MNa)+, 367.1 (MH)+, 349.1 (M – OH)+; *vmax* 3343, 2924, 1708, 1045, 753 cm-1. HRMS (LSIMS, +ve ion) Calculated for C21H22N2O4 (M+H)+ 367.1645. Found 367.1658.

*Data for* ***25****,* isolated as a 1:1 ratio of diastereoisomers **a** and **b**. 1H NMR (CD3OD, 400 MHz)  9.97 (1H + 1H, s, NH3 **a** and **b**), 7.70 (1H, d, *J = 8.0 Hz*, H6 **a** or **b**), 7.60 (1H, d, *J = 8.0 Hz*, H6 **a** or **b**), 7.40 – 7.34 (2H + 2H, m, Ar-H **a** and **b**), 7.22 – 7.12 (5H + 5H, m, Ar-H **a** and **b**), 6.99 – 6.94 (1H + 1H, m, Ar-H **a** and **b**), 6.72 – 6.69 (1H + 1H, m, Ar-H **a** and **b**), 5.98 (1H, d, *J = 10.5 Hz*, H5’’ **a** or **b**), 5.96 (1H, d, *J = 10.5 Hz*, H5’’ **a** or **b**), 5.89 – 5.87 (1H + 1H, m, H1’ **a** and **b**), 5.76 (1H, d, *J = 8.0 Hz*, H5 **a** or **b**), 5.66 (1H, d, *J = 8.0 Hz*, H5 **a** or **b**), 4.78 (1H + 1H, d, *J = 4.0 Hz*), 4.36 – 4.06 (5H + 5H, m, H2’ **a** and **b**, H3’ **a** and **b**, H4’ **a** and **b**, CH2 5’ **a** and **b**), 4.02 (3H, s, OC*H*3 **a** or **b**), 4.00 (3H, s, OC*H*3 **a** or **b**), 3.70 (1H + 1H, d, *J = 7.5 Hz*, H3’’ **a** and **b**), 3.65 (1H + 1H, ddd, *J = 3.0, 7.5, 10.5 Hz*, H4’’ **a** and **b**), 3.25 (1H + 1H, dd, *J = 3.0, 16.0 Hz*, C*H*HOCO **a** and **b**), 3.02 (1H + 1H, dd, *J = 3.0, 16.0 Hz*, CH*H*OCO **a** and **b**), 1.71 (3H, s, 2’’-C*H*3 **a** or **b**), 1.69 (3H, s, 2’’-C*H*3 **a** or **b**); 13C NMR (CD3OD, 125 MHz)  199.3, 199.2, 171.5, 169.0, 167.7, 166.0, 157.8, 152.3, 142.6, 142.3, 137.4, 131.1, 129.6, 128.5, 128.1, 126.1, 125.8, 124.7, 122.7, 112.3, 111.7, 103.2, 91.2, 91.1, 86.7, 86.7, 83.0, 82.9, 75.1, 75.0, 74.9, 72.1, 71.1, 65.6, 65.5, 59.2, 59.0, 57.3, 57.3, 56.5, 56.3, 48.9, 20.0; m/z (micrOTOF, +ve ion) 701.2 (MNa)+; *vmax* 3392, 1679, 1399 cm-1; HRMS (micrOTOF, +ve ion) Calculated for C33H34N4O12Na, 701.2065. Found 701.2069.

*Data for* ***26***, isolatedas a 1:1 ratio of diastereoisomers **a** and **b**. 1H NMR(CD3OD, 500 MHz)  7.72 – 7.66 (3H + 3H, m, Ar-H **a** and **b**, H6 **a** and **b**), 7.53 – 7.43 (4H +4H, m, Ar-H **a** and **b**), 7.31 (1H + 1H, dt, *J = 1.5, 7.5 Hz*, Ar-H **a** and **b**), 7.16 (1H + 1H, d, *J = 7.5 Hz*, Ar-H **a** and **b**), 7.12 (1H + 1H, d, *J = 8.5 Hz*, Ar-H **a** and **b**), 6.42 (1H + 1H, s), 6.00 (1H + 1H, dd, *J = 6.5, 6.5 Hz*), 5.86 – 5.85 (1H + 1H, m, H1’ **a** and **b**), 5.79 – 5.77 (1H + 1H, m, H5 **a** and **b**), 5.53 (1H + 1H, d, *J = 6.5 Hz*, H5’’ **a** and **b**), 4.50 – 4.43 (1H + 1H, m, C*H*H 5’ **a** and **b**), 4.36 – 4.31 (1H + 1H, m, CH*H* 5’ **a** and **b**), 4.24 (1H + 1H, dd, *J = 4.5, 4.5 Hz*, H2’ **a** and **b**), 4.18 – 4.14 (2H + 2H, m, H3’ **a** and **b**, H4’ **a** and **b**), 3.87 – 3.80 (2H + 2H, m, H4’’ **a** and **b**, H3’’ **a** and **b**), 3.77 (3H + 3H, s, OC*H*3 **a** and **b**), 2.87 – 2.80 (4H + 4H, m, C*H* 2 1’’’ **a** and **b**, C*H*2 2’’’ **a** and **b**), 1.86 (3H + 3H, s, 2’’-C*H*3 **a** and **b**); 13C NMR (CD3OD, 125 MHz)  172.2, 170.4, 170.4, 170.1, 164.7, 157.9, 150.8, 141.1, 137.2, 131.5, 130.0, 128.7, 126.4, 122.2, 122.1, 121.5, 116.7, 111.5, 101.5, 98.3, 90.8, 90.4, 90.3, 81.5, 73.6, 73.6, 69.8, 64.7, 63.6, 55.3, 50.5, 49.2, 28.4, 28.1, 17.2; m/z (ESI, +ve ion) 731.1 (MK)+, 715.2 (MNa)+, 693.2 (MH)+; *vmax* 2972, 1674, 1130 cm-1; HRMS (ESI, +ve ion) Calculated for C34H36N4O12 (M+H)+ 693.2402. Found 693.2412.
